# Supplementary material for: Design, Synthesis and Assay of Novel Methylxanthine–Alkynylmethylamine Derivatives as Acetylcholinesterase Inhibitors
Source: Molecules. 2022 Dec 11;27(24):8787. doi: 10.3390/molecules27248787 (PMC9788520; doi:10.3390/molecules27248787)
Supplement: Supplementary file 1 [file molecules-27-08787-s001.zip › molecules-2039644-supplementary.pdf]

Supporting Information

Table of content

1. S2-S71. NMR ^{1}H and ^{13}C spectra of compounds **15**, **16**, **18**, **19**, **22**, **23**, **24**, **28-33**, **35**, **39-41**, **51-59**, **60**, **61**, **64-66**, **68-70**. **p. 2-71**

2**. Fig. S5A-E**. Plots dependency of percent of inhibition – concentration for AChE inhibition by compounds **28**, **64**, **65**, **66**, **70.** **p. 72**

3. Fig. S6A-D. Calculated RMSD of ligand atomic coordinates over simulation time, obtained as a result of molecular dynamics simulation for protein-ligand complexes. **p.73**

8-(2-Aminophenyl)-1,3,7-trimethyl-3,7-dihydro-1*H*-purine-2,6-dione (**15**) (^{1}H NMR, 400 MHz, $CDCl_3$)

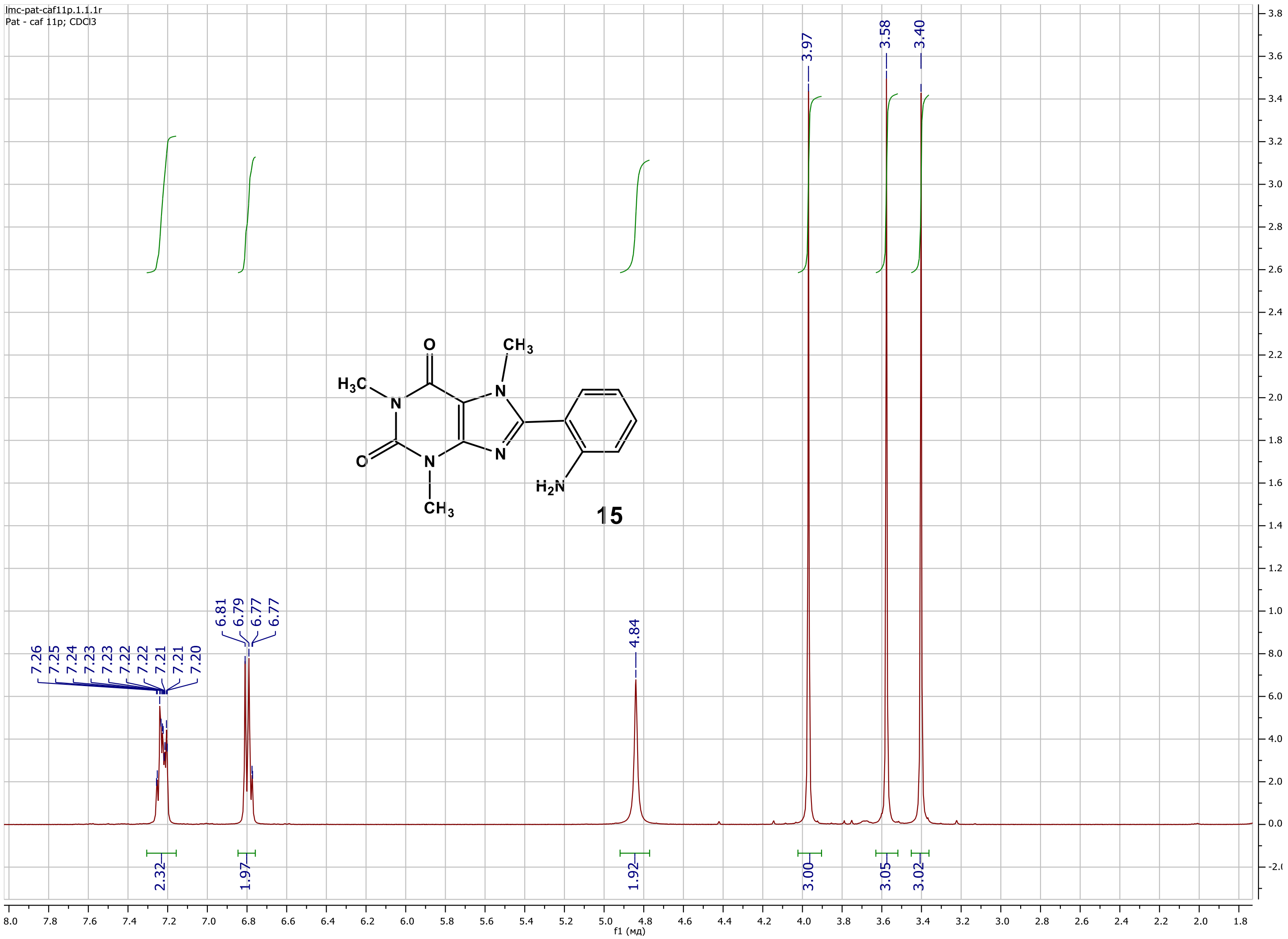

8-(2-Aminophenyl)-1,3,7-trimethyl-3,7-dihydro-1*H*-purine-2,6-dione (**15**) (^{13}C NMR, 101 MHz, $CDCl_3$)

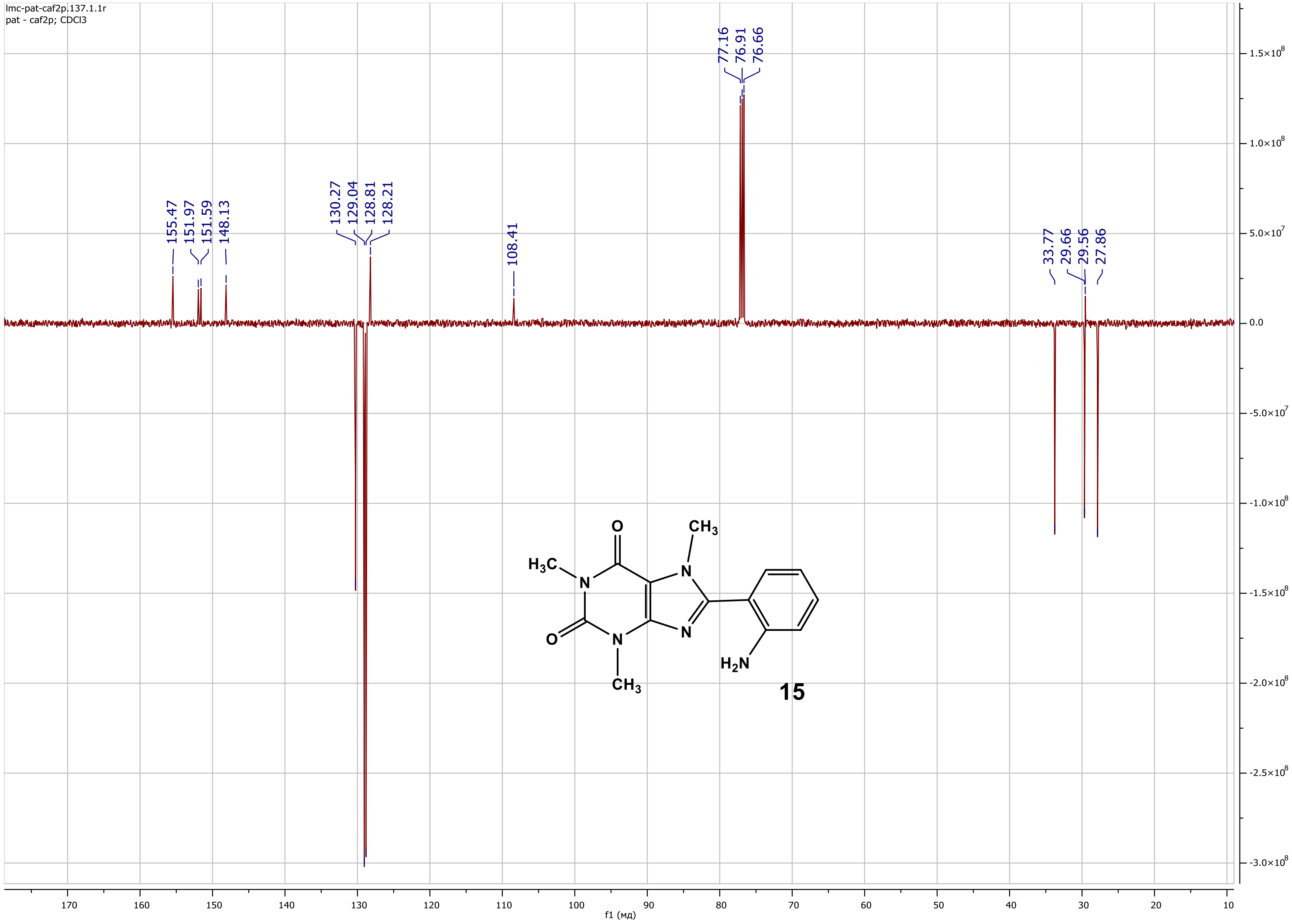

8-(3-Methoxyphenyl)-1,3,7-trimethyl-3,7-dihydro-1*H*-purine-2,6-dione (**16**) (^{1}H NMR, 500 MHz, $CDCl_3$)

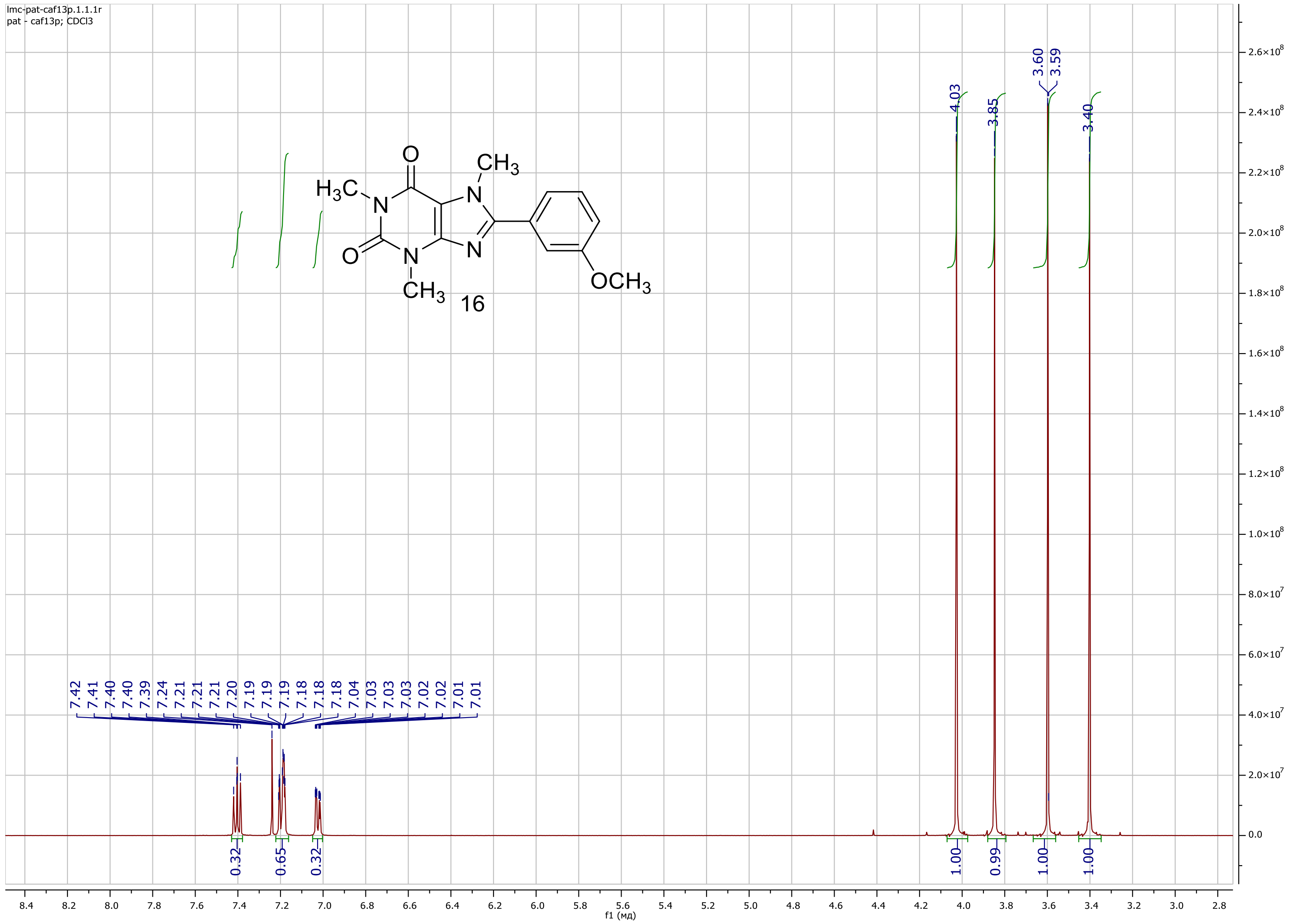

8-(3-Methoxyphenyl)-1,3,7-trimethyl-3,7-dihydro-1*H*-purine-2,6-dione (**16**) (^{13}C NMR, 126 MHz, $CDCl_3$)

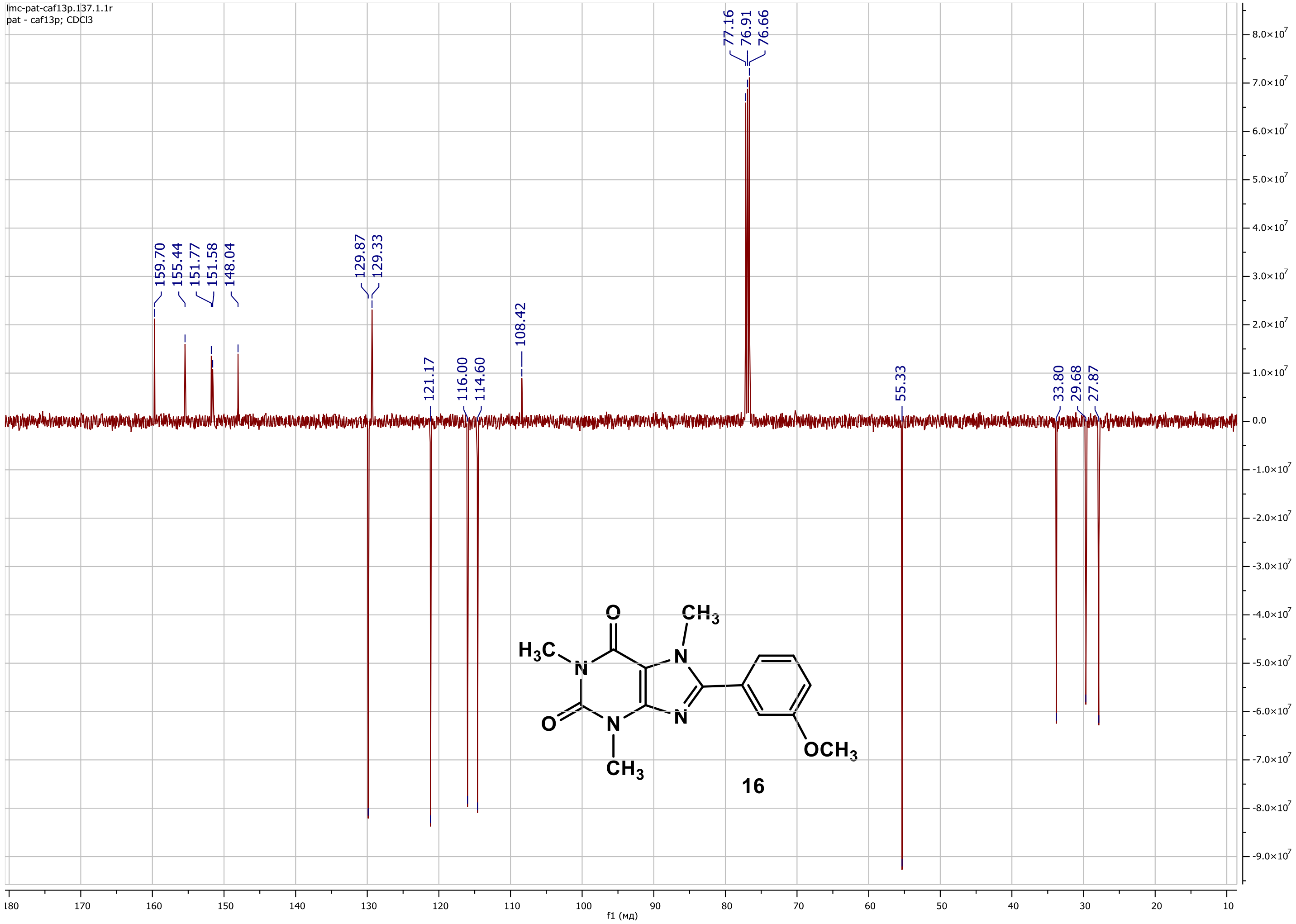

8-(2,3-Dimethoxyphenyl)-1,3,7-trimethyl-3,7-dihydro-1*H*-purine-2,6-dione (**18**) (^{1}H NMR, 400 MHz, $CDCl_3$)

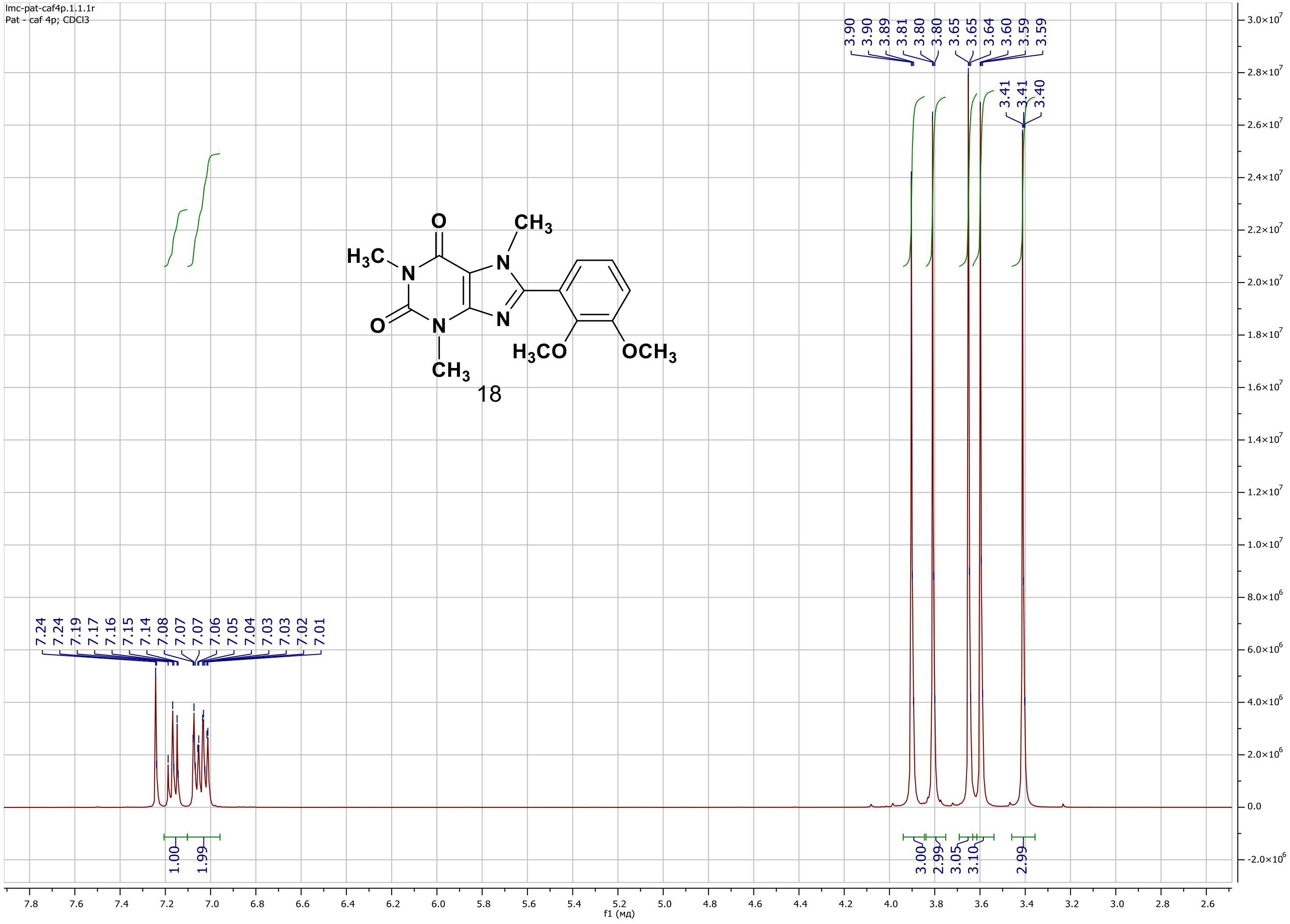

8-(2,3-Dimethoxyphenyl)-1,3,7-trimethyl-3,7-dihydro-1*H*-purine-2,6-dione (**18**) (^{13}C NMR, 101 MHz, $CDCl_3$)

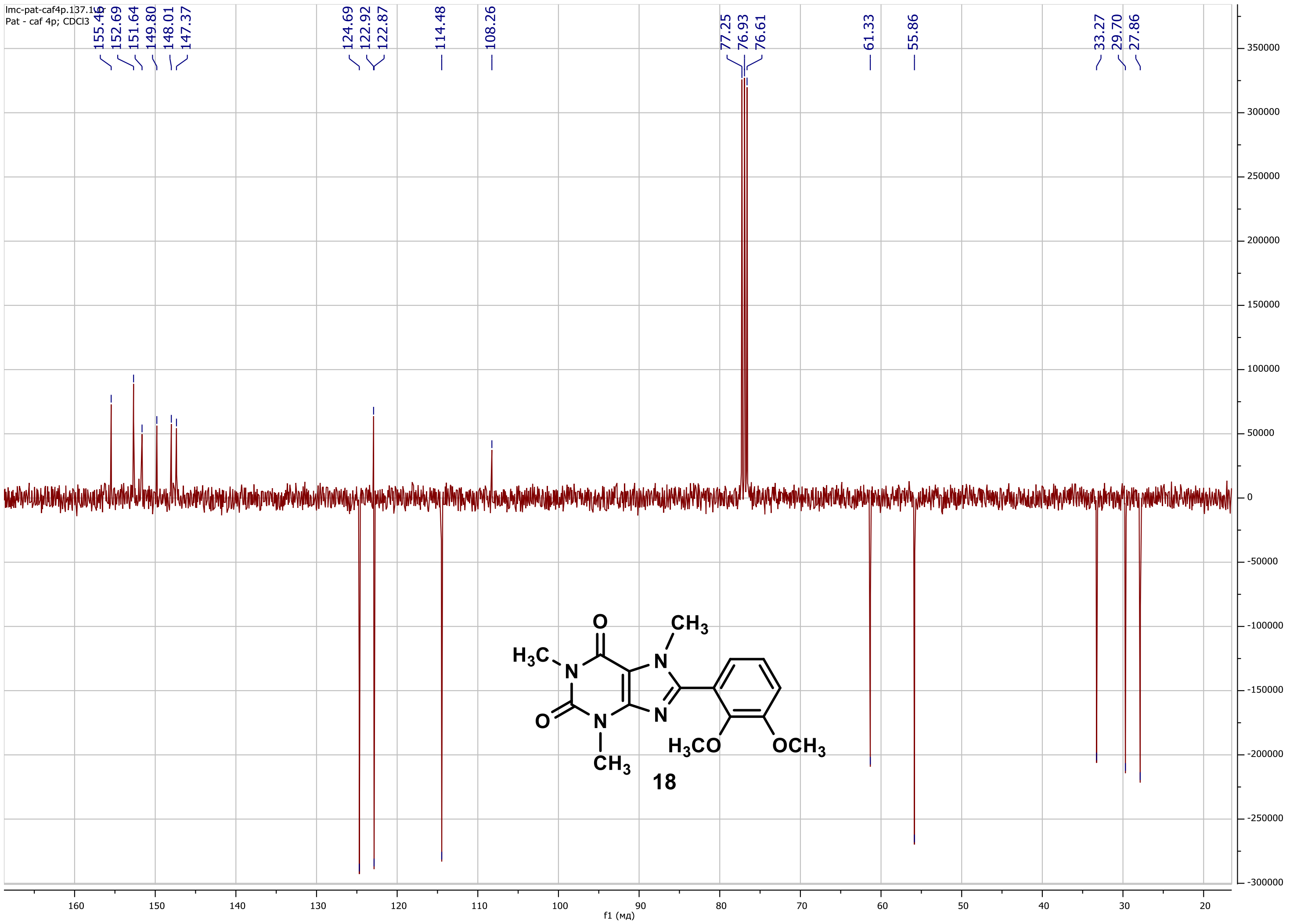

8-(3,4,5-Trimethoxyphenyl)-1,3,7-trimethyl-3,7-dihydro-1*H*-purine-2,6-dione (**19**) (^{1}H NMR, 400 MHz, $CDCl_3$)

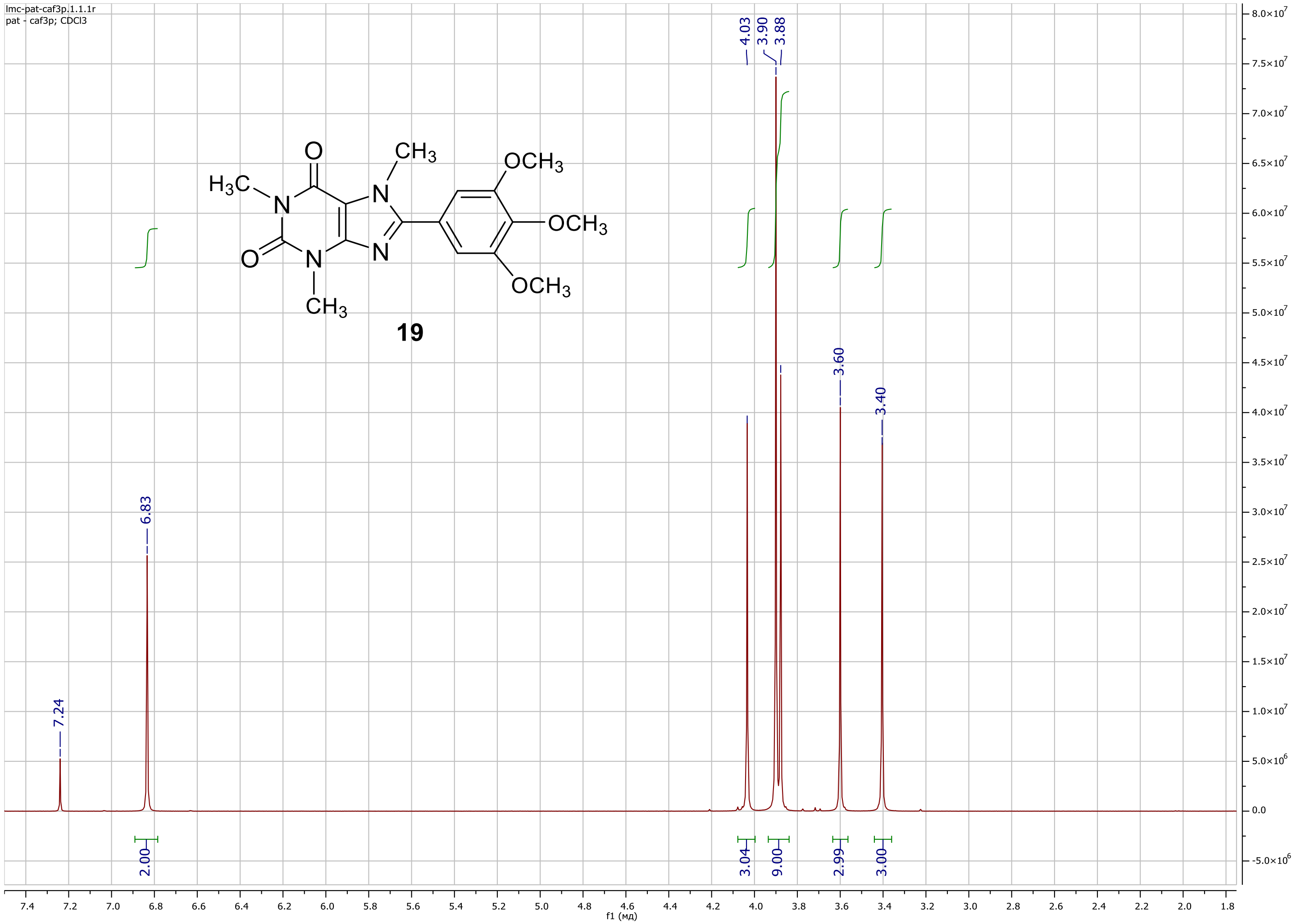

8-(3,4,5-Trimethoxyphenyl)-1,3,7-trimethyl-3,7-dihydro-1*H*-purine-2,6-dione (**19**) (^{13}C NMR, 101 MHz, $CDCl_3$)

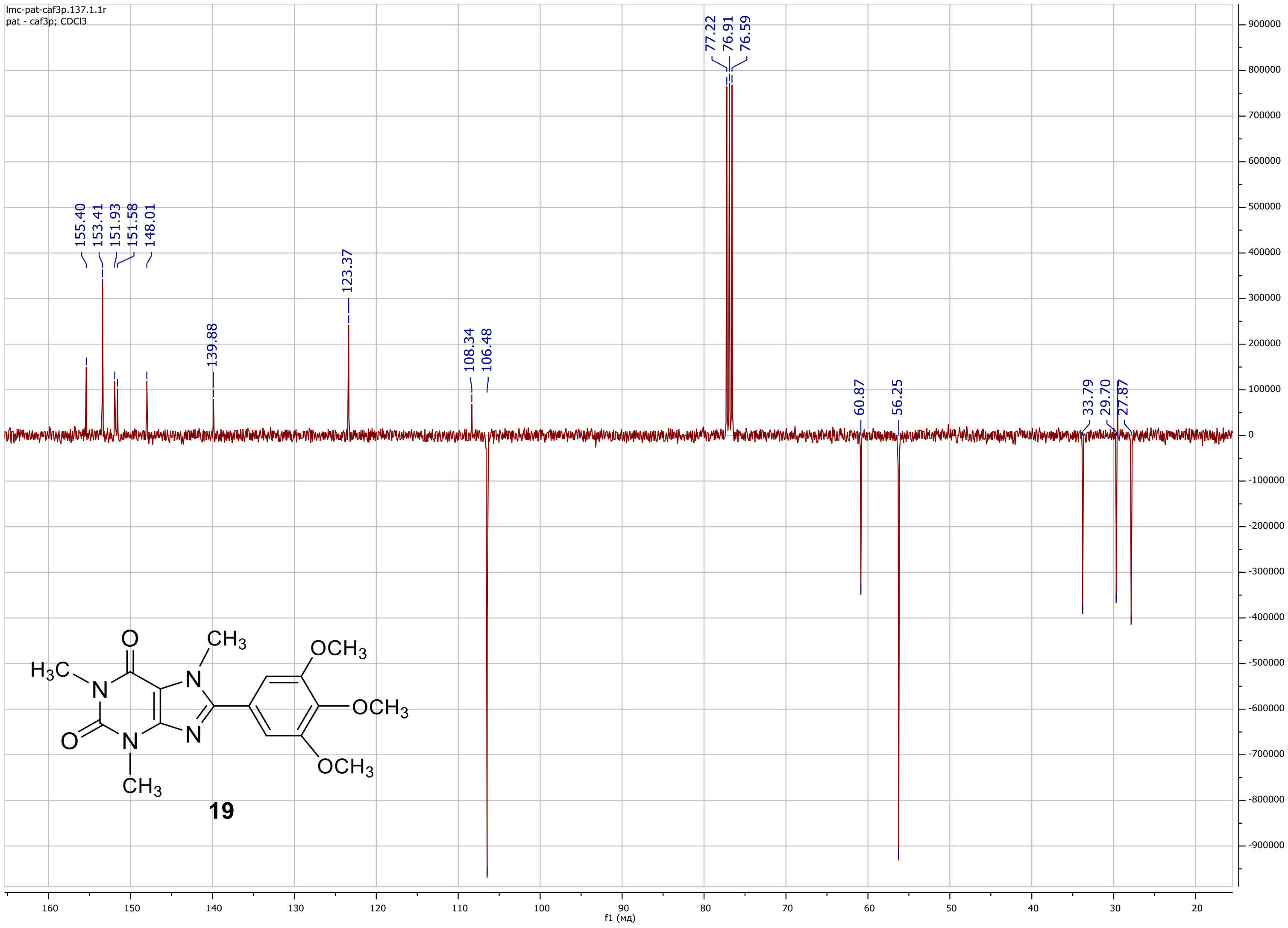

8-(Furan-3-yl)-1,3,7-trimethyl-3,7-dihydro-1*H*-purine-2,6-dione (**22**) (^{1}H NMR, 400 MHz, $CDCl_3$)

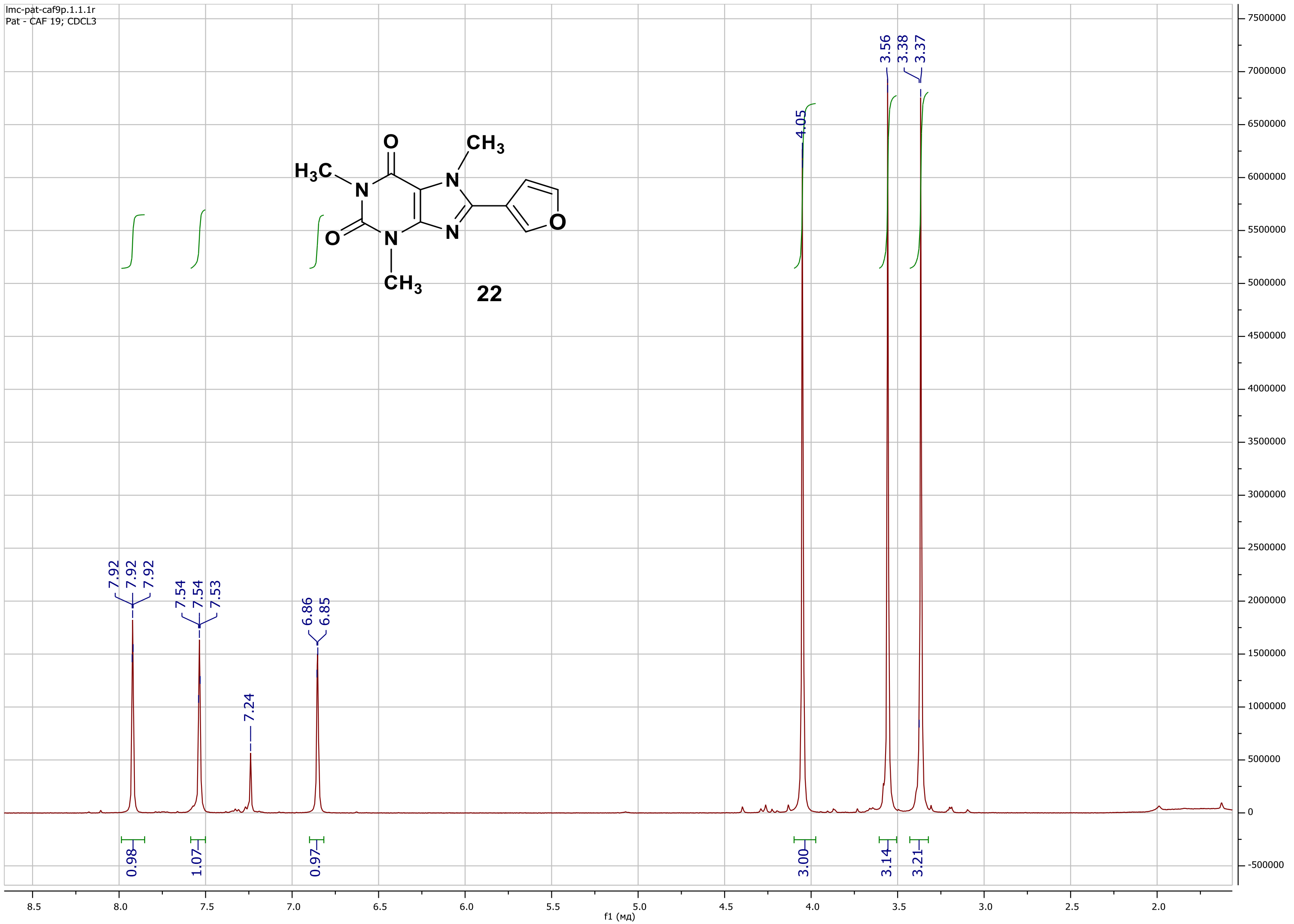

8-(Furan-3-yl)-1,3,7-trimethyl-3,7-dihydro-1*H*-purine-2,6-dione (**22**) (^{13}C NMR, 101 MHz, $CDCl_3$)

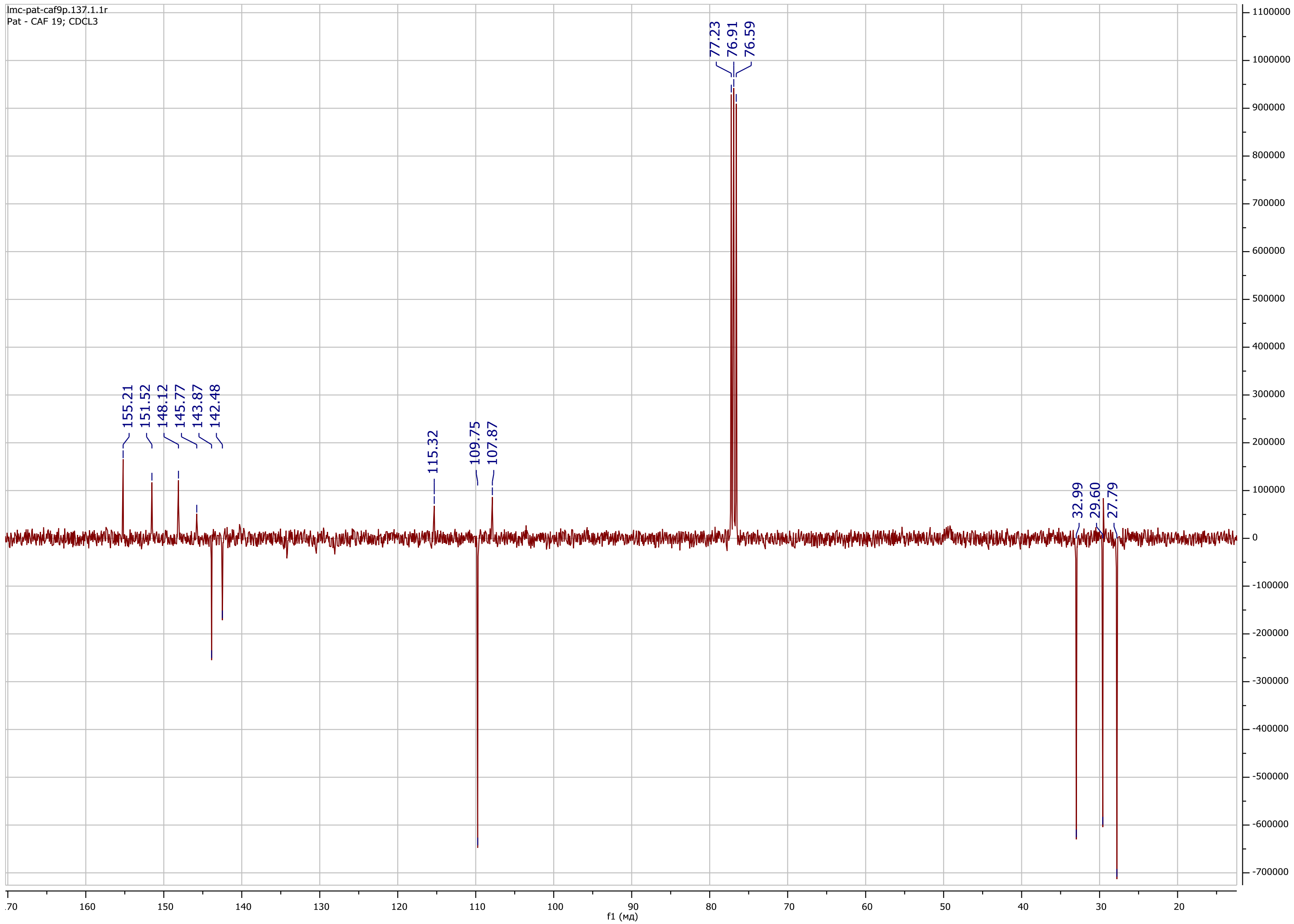

8-(1*H*-Indol-5-yl)-1,3,7-trimethyl-3,7-dihydro-1*H*-purine-2,6-dione (**23**) (^{1}H NMR, 500 MHz, $CDCl_3$)

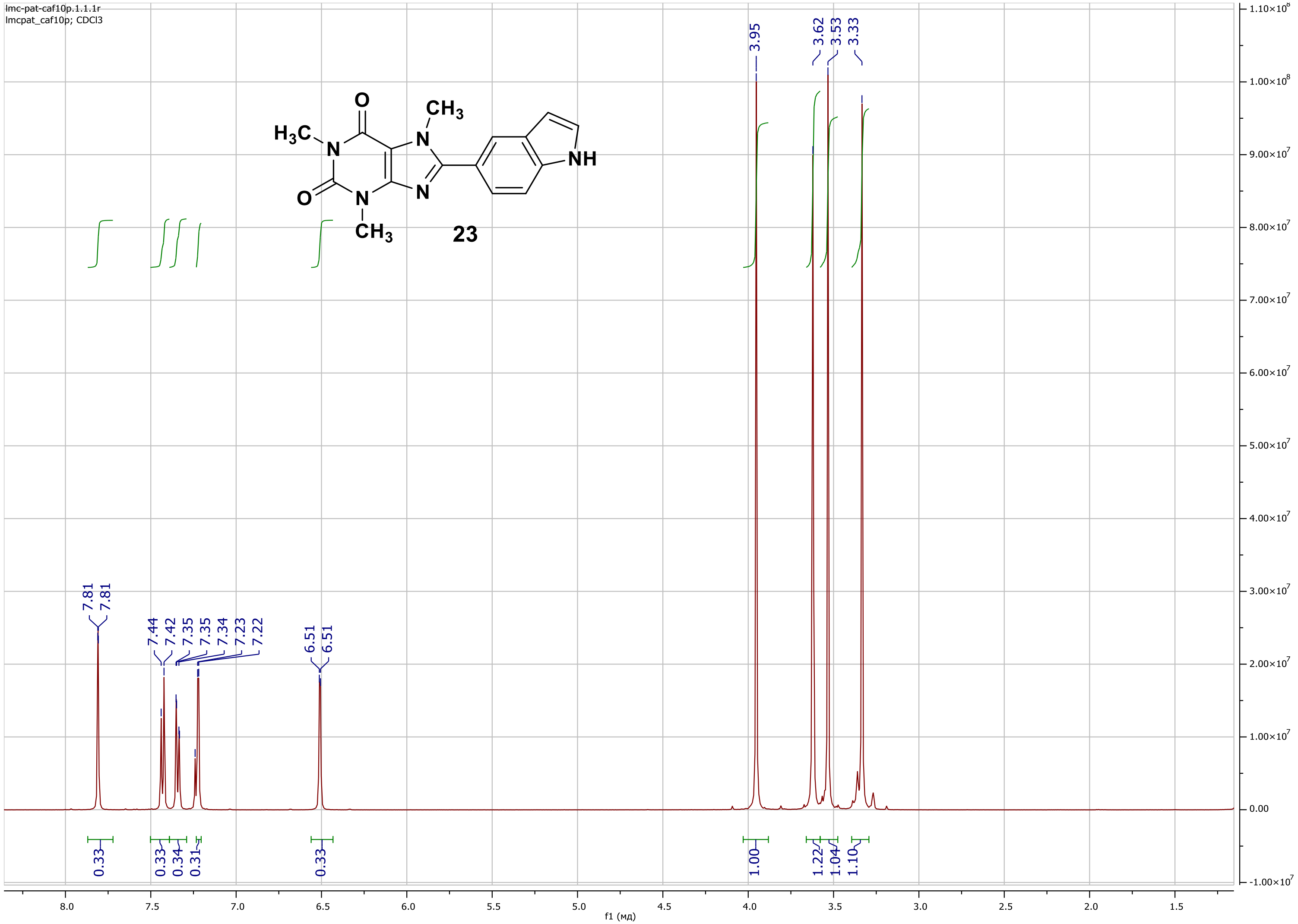

8-(1*H*-Indol-5-yl)-1,3,7-trimethyl-3,7-dihydro-1*H*-purine-2,6-dione (**23**) (^{13}C NMR, 126 MHz, $CDCl_3$+CD_3OD)

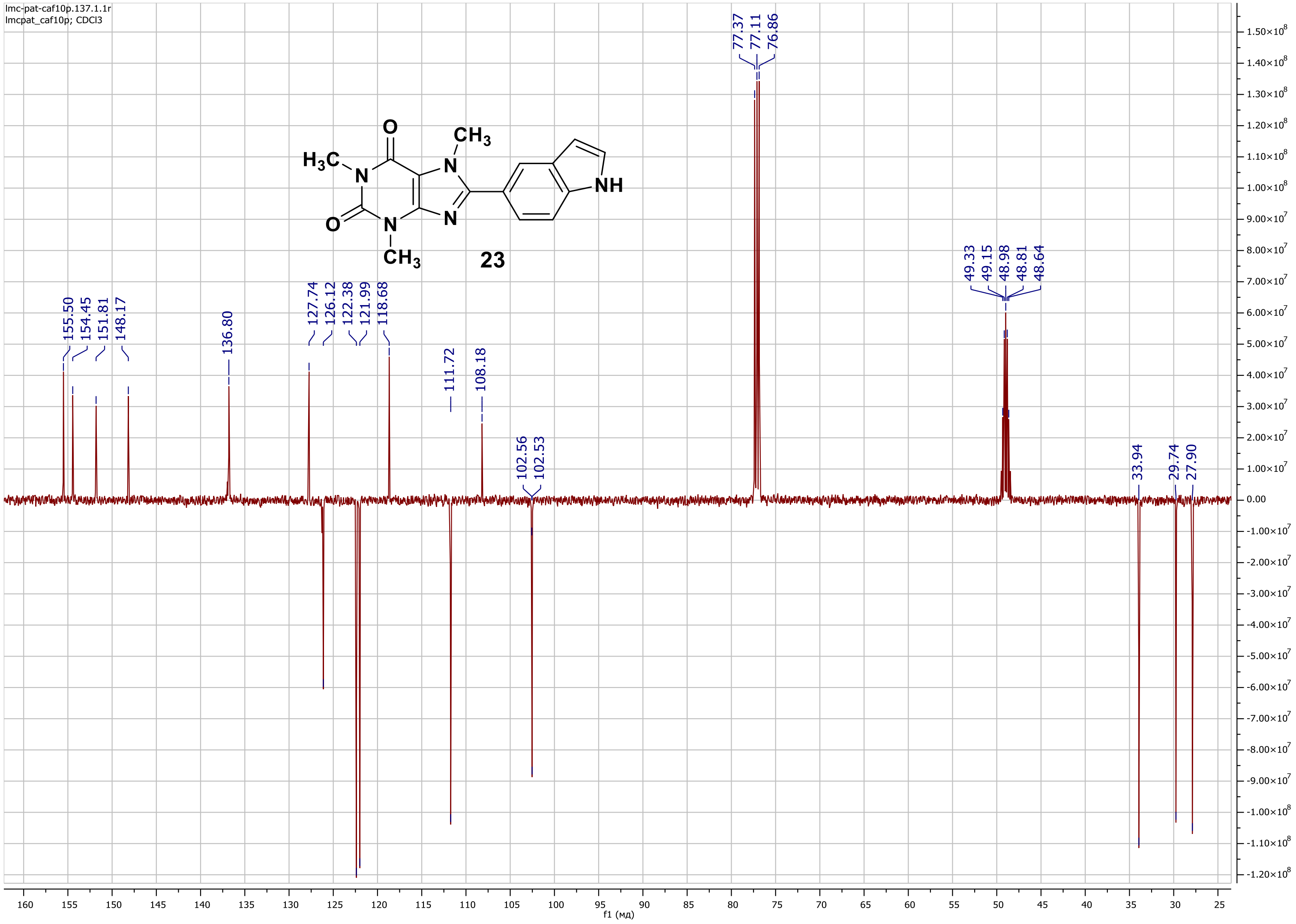

8-Ethynyl-1,3,7-trimethyl-3,7-dihydro-1*H*-purine-2,6-dione (**24**) (^{1}H NMR, 400 MHz, $CDCl_3$)

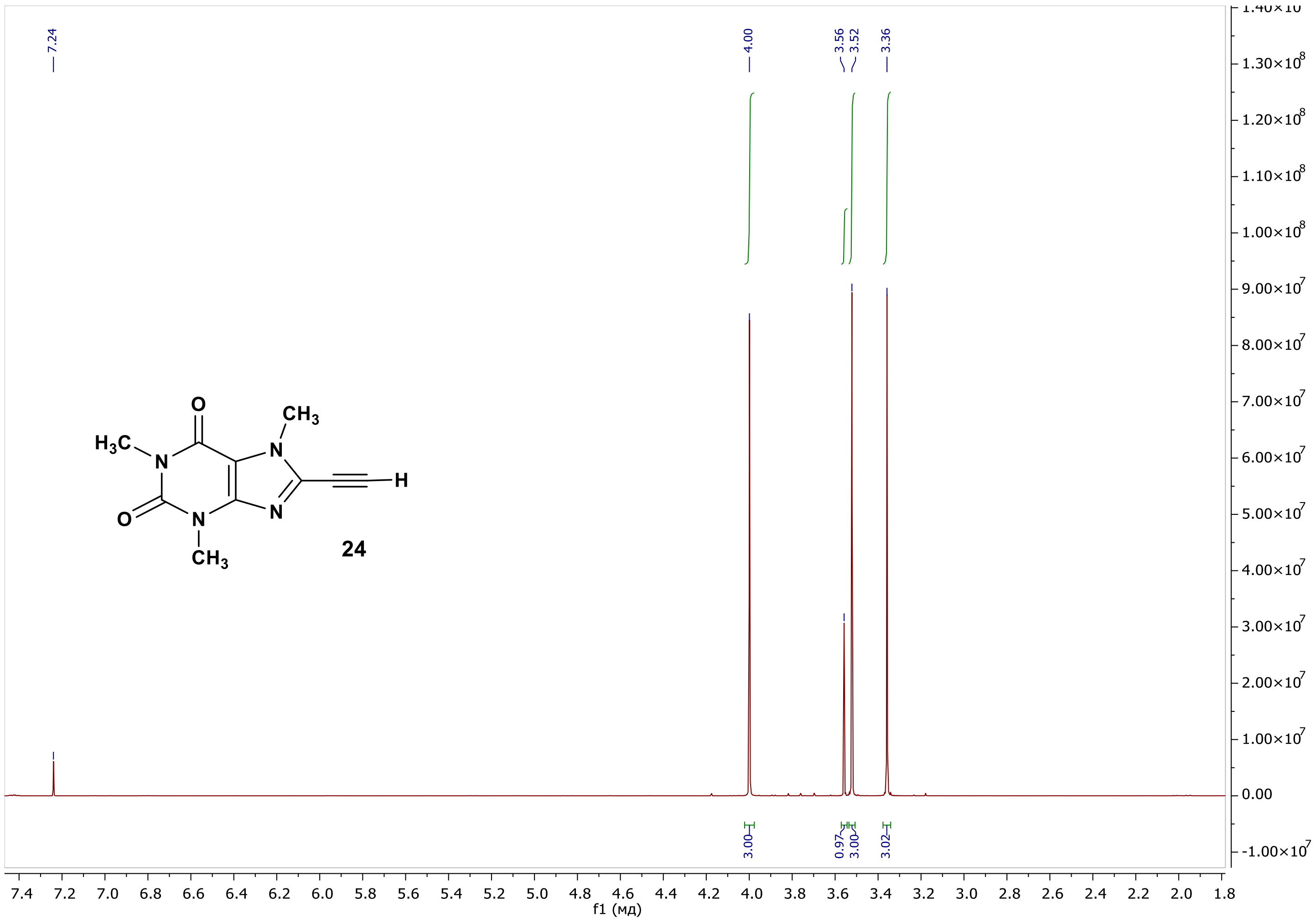

8-Ethynyl-1,3,7-trimethyl-3,7-dihydro-1*H*-purine-2,6-dione (**24**) (^{13}C NMR, 101 MHz, $CDCl_3$)

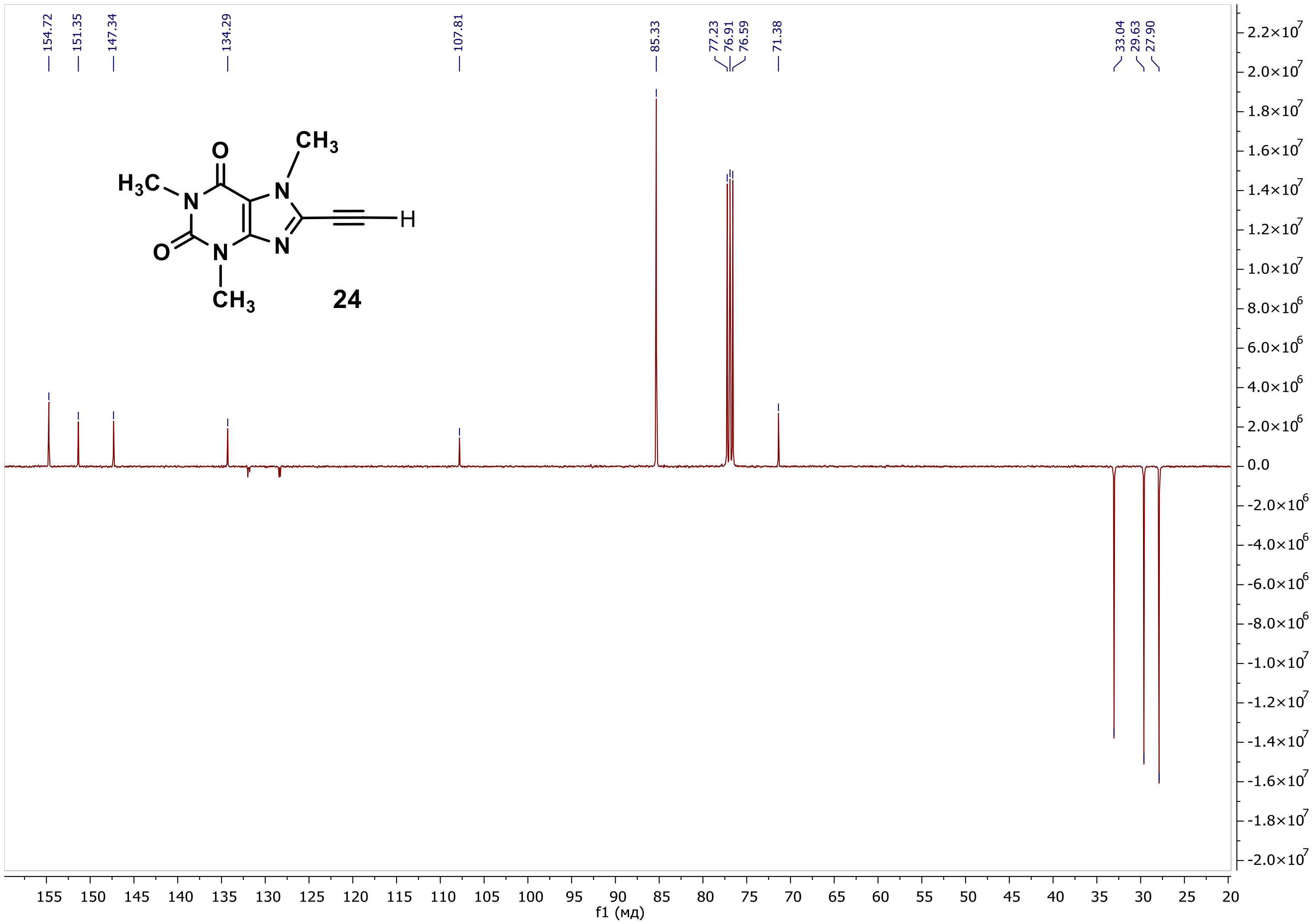

1,3,7-Trimethyl-8-((trimethylsilyl)ethynyl)-3,7-dihydro-1*H*-purine-2,6-dione (**26**) (^{1}H NMR, 300 MHz, $CDCl_3$)

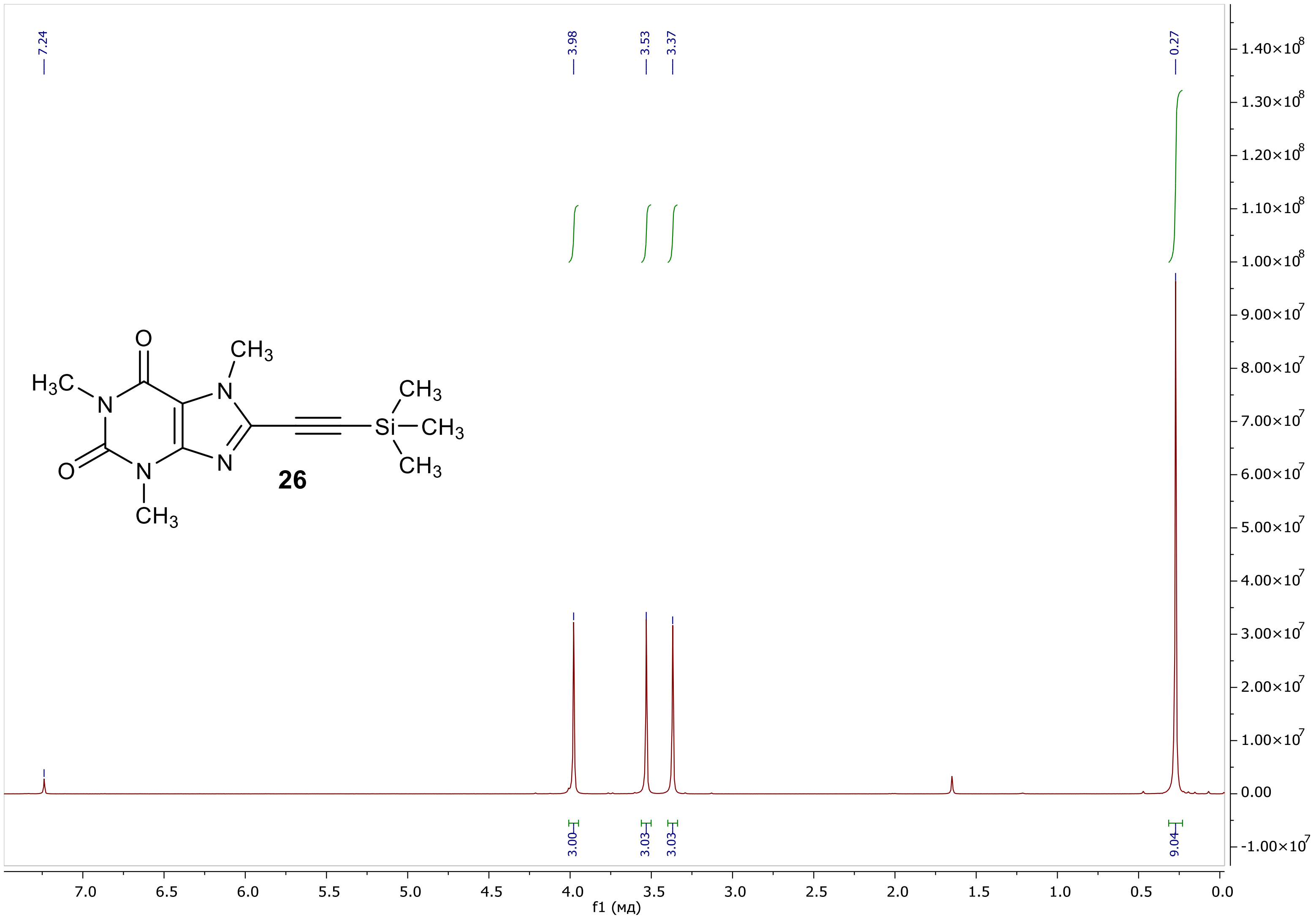

1,3,7-Trimethyl-8-((trimethylsilyl)ethynyl)-3,7-dihydro-1*H*-purine-2,6-dione (**26**) (^{13}C NMR, 75 MHz, $CDCl_3$)

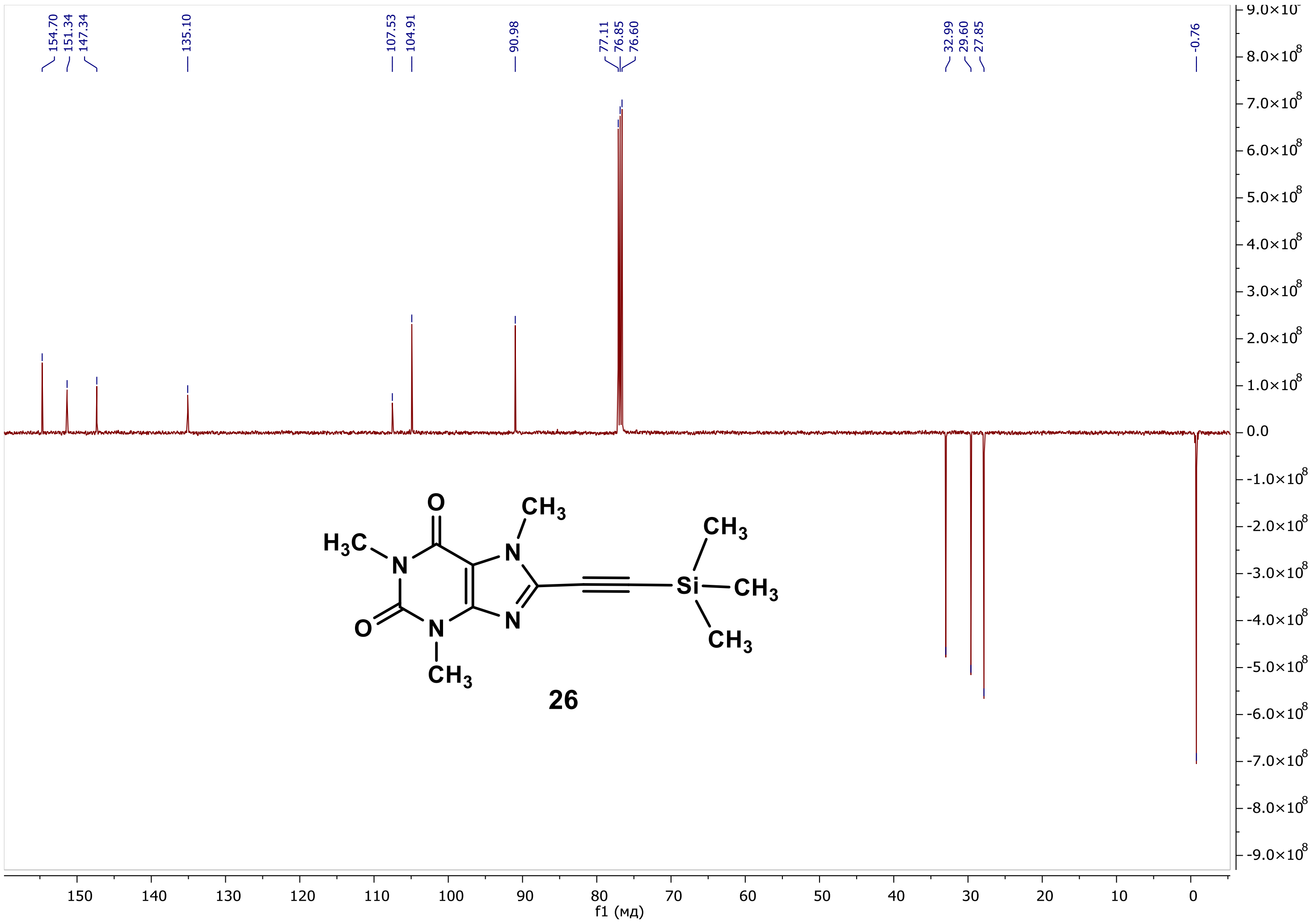

1,3,7-Trimethyl-8-(1-(*p*-tolyl)-1*H*-1,2,3-triazol-4-yl)-3,7-dihydro-1*H*-purine-2,6-dione (**28**) (^{1}H NMR, 400 MHz, $CDCl_3$)

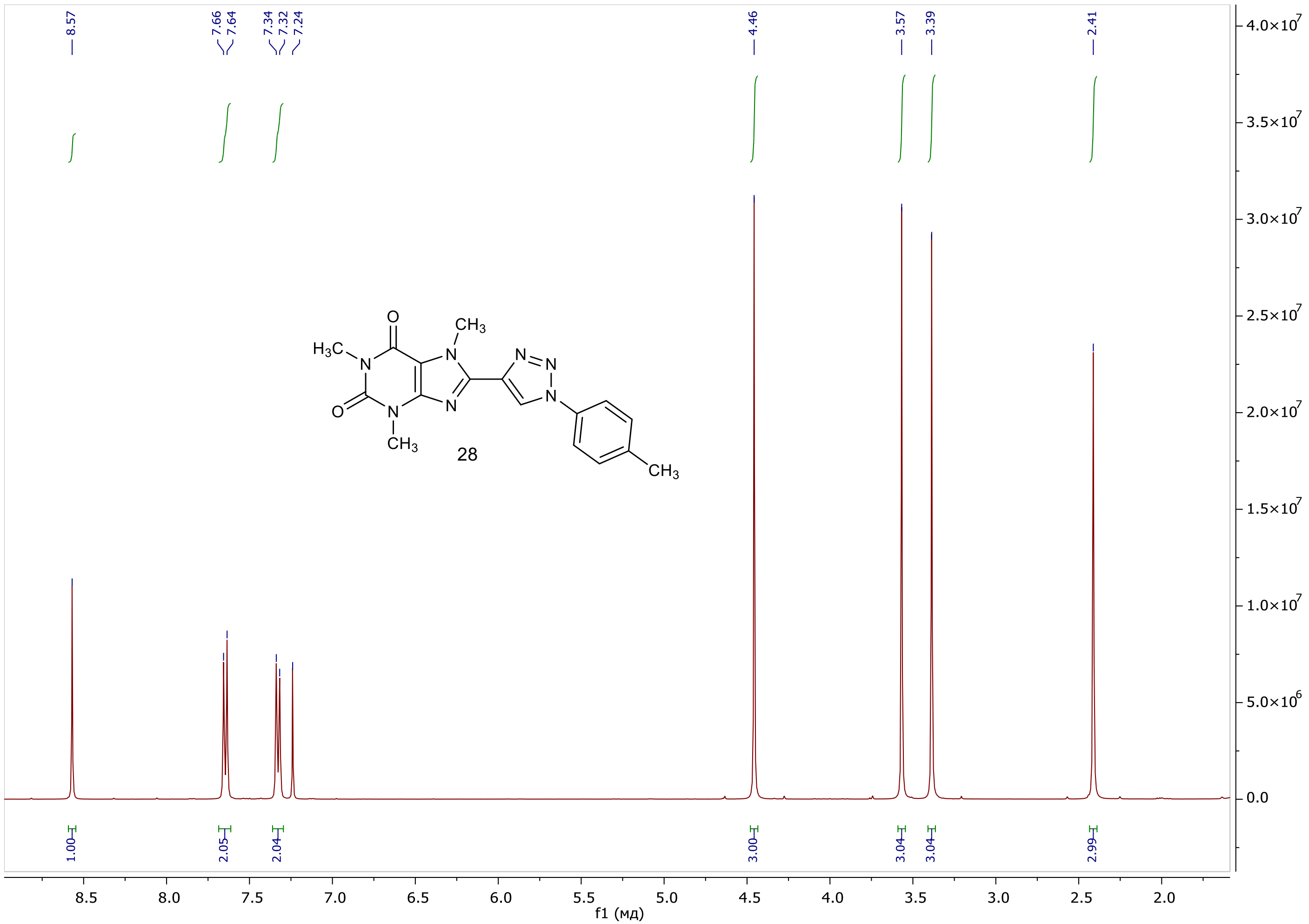

1,3,7-Trimethyl-8-(1-(*p*-tolyl)-1*H*-1,2,3-triazol-4-yl)-3,7-dihydro-1*H*-purine-2,6-dione (**28**) (^{13}C NMR, 126 MHz, $CDCl_3$)

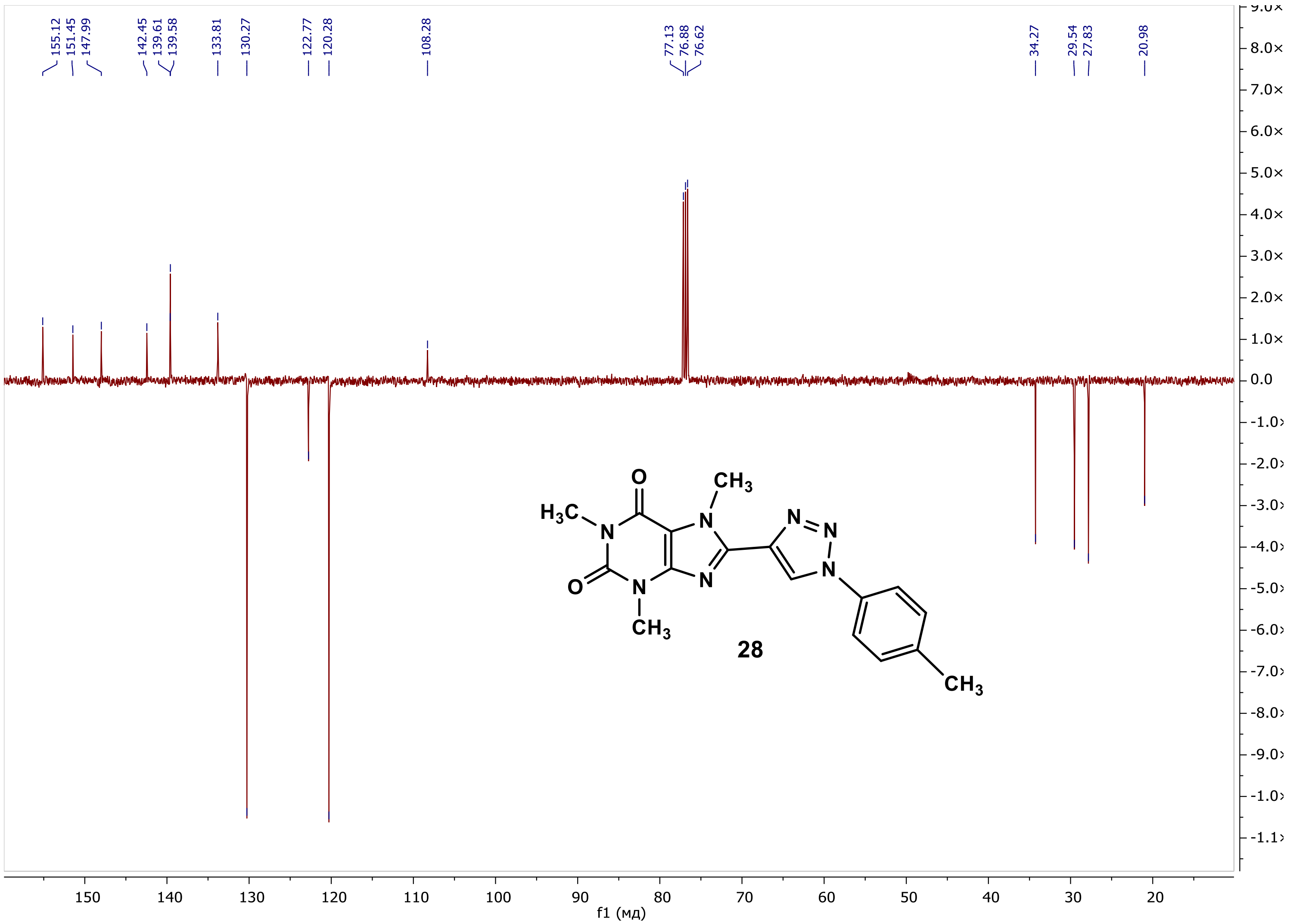

1,3,7-Trimethyl-8-(1-(4-nitrophenyl)-1*H*-1,2,3-triazol-4-yl)-3,7-dihydro-1*H*-purine-2,6-dione (**29**) (^{1}H NMR, 400 MHz, $CDCl_3$)

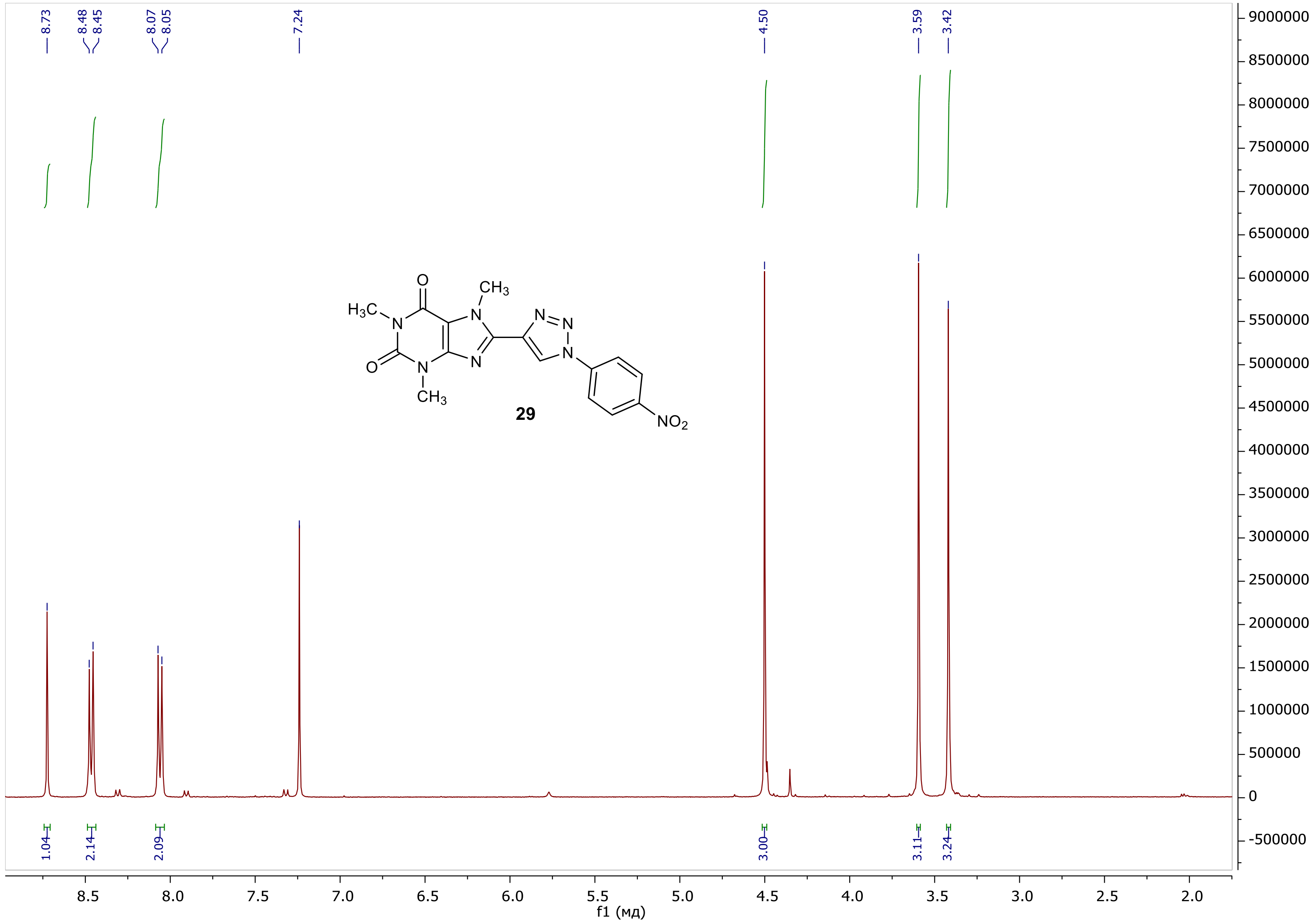

1,3,7-Trimethyl-8-(1-(4-nitrophenyl)-1*H*-1,2,3-triazol-4-yl)-3,7-dihydro-1*H*-purine-2,6-dione (**29**) (^{13}C NMR, 126 MHz, $CDCl_3$)

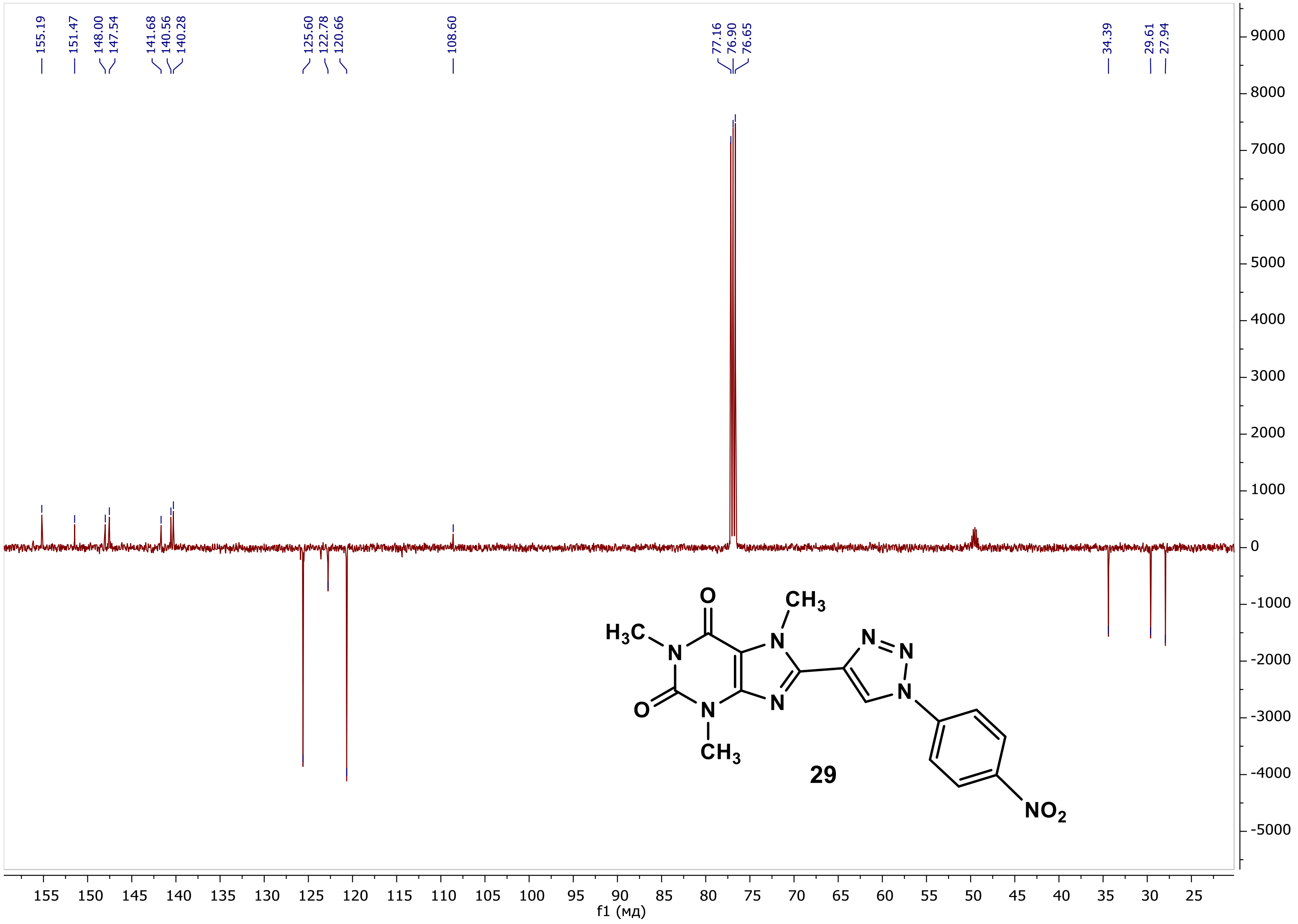

8-(1-Benzyl-1*H*-1,2,3-triazol-4-yl)-1,3,7-trimethyl-3,7-dihydro-1*H*-purine-2,6-dione (**30**) (^{1}H NMR, 300 MHz, $CDCl_3$)

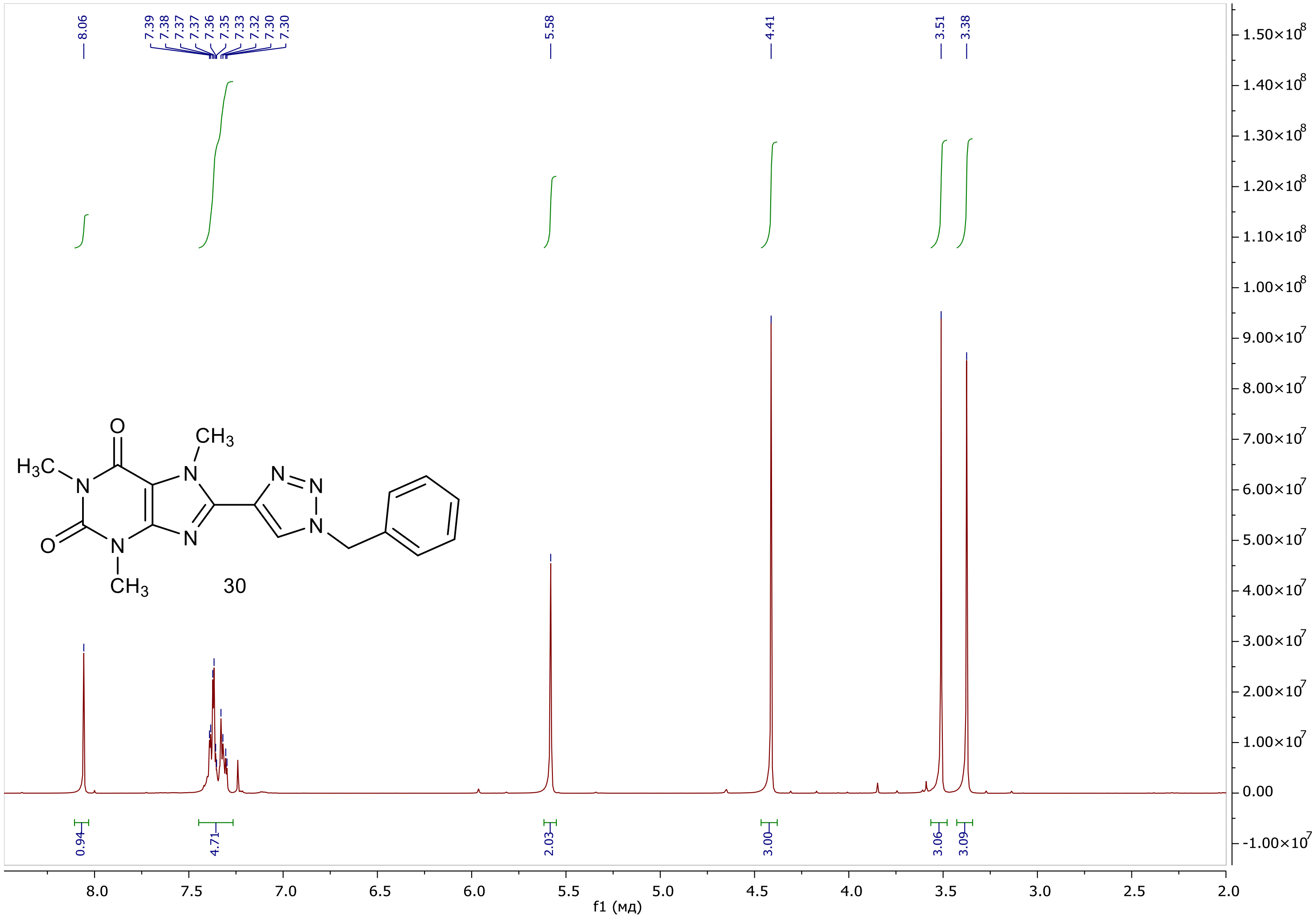

8-(1-Benzyl-1*H*-1,2,3-triazol-4-yl)-1,3,7-trimethyl-3,7-dihydro-1*H*-purine-2,6-dione (**30**) (^{13}C NMR, 75 MHz, $CDCl_3$)

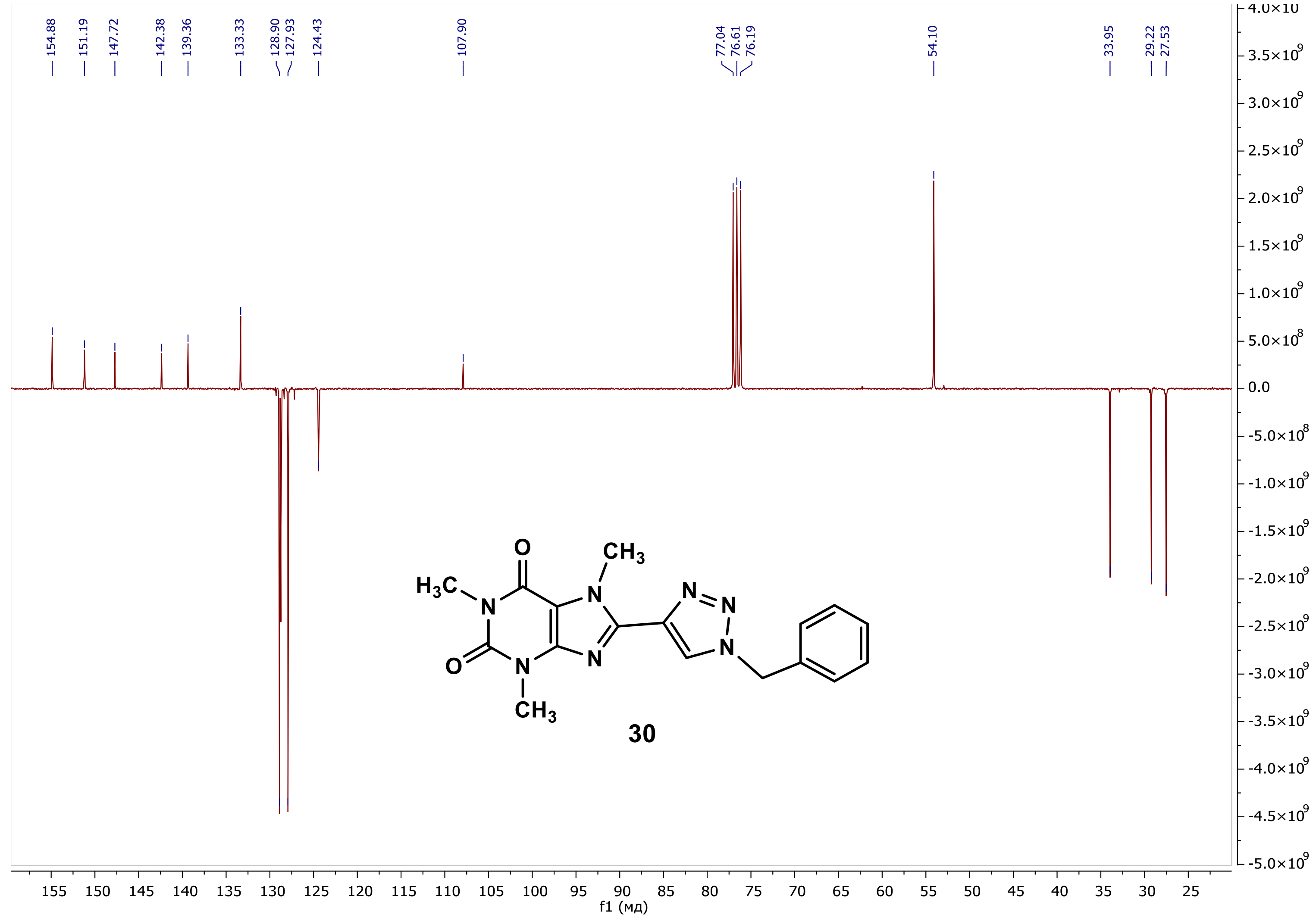

8-(1-Butyl-1*H*-1,2,3-triazol-4-yl)-1,3,7-trimethyl-3,7-dihydro-1*H*-purine-2,6-dione (**31**) (^{1}H NMR, 300 MHz, CDCl$_3$)

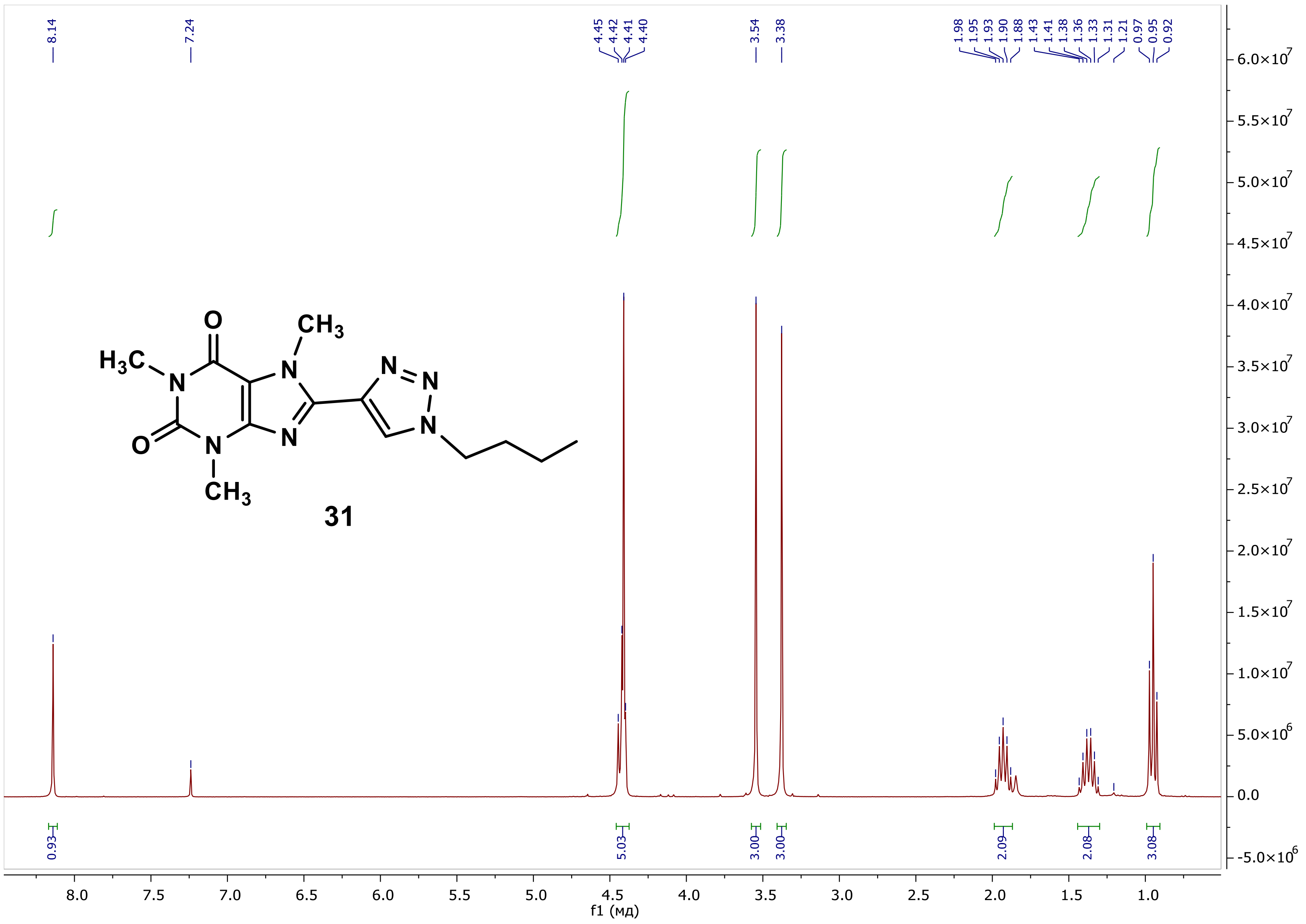

8-(1-Butyl-1*H*-1,2,3-triazol-4-yl)-1,3,7-trimethyl-3,7-dihydro-1*H*-purine-2,6-dione (**31**) (^{13}C NMR, 126 MHz, $CDCl_3$)

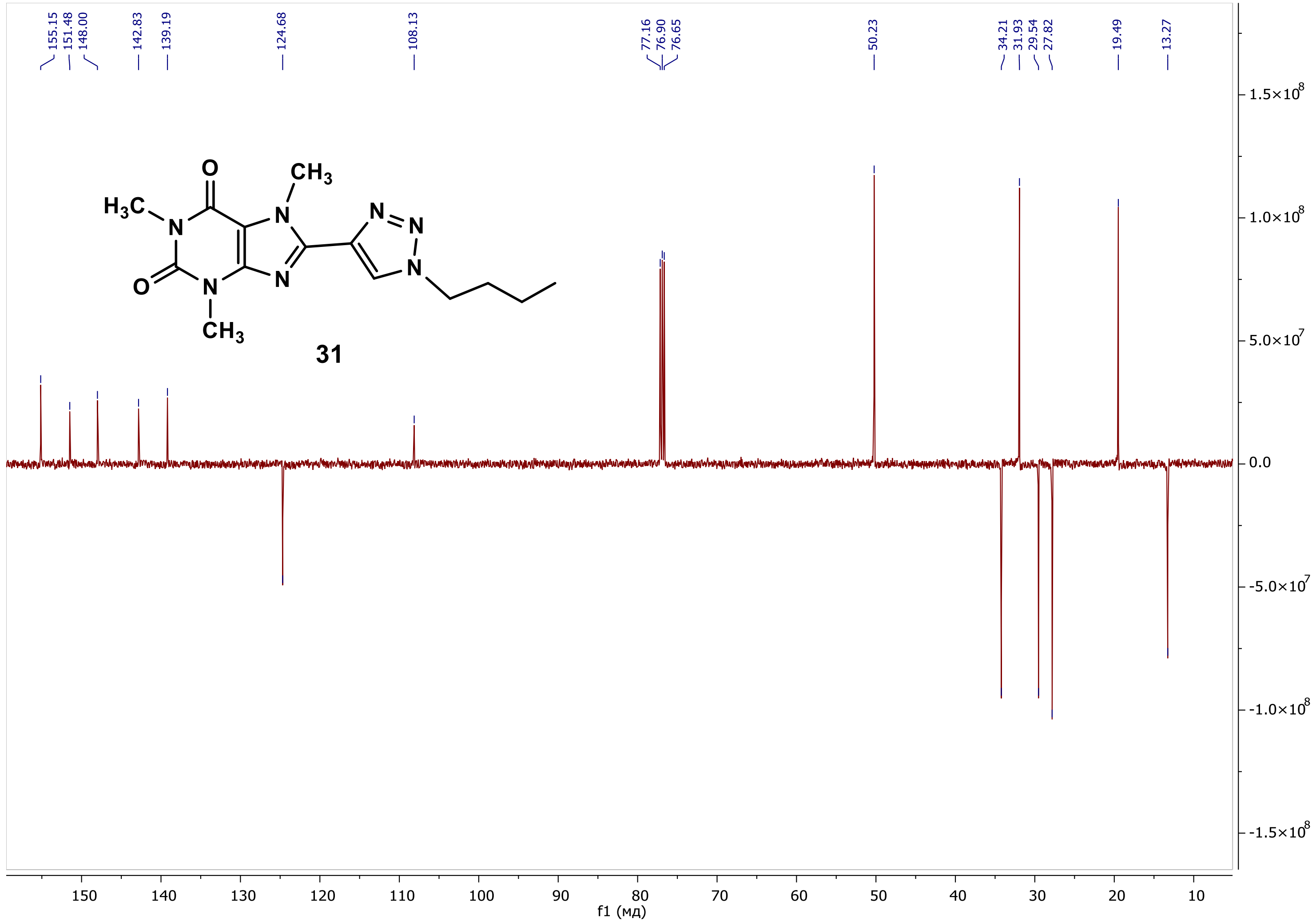

8-(1-(2-Hydroxyethyl)-1*H*-1,2,3-triazol-4-yl)-1,3,7-trimethyl-3,7-dihydro-1*H*-purine-2,6-dione (**32**) (^{1}H NMR, 300 MHz, $CDCl_3$)

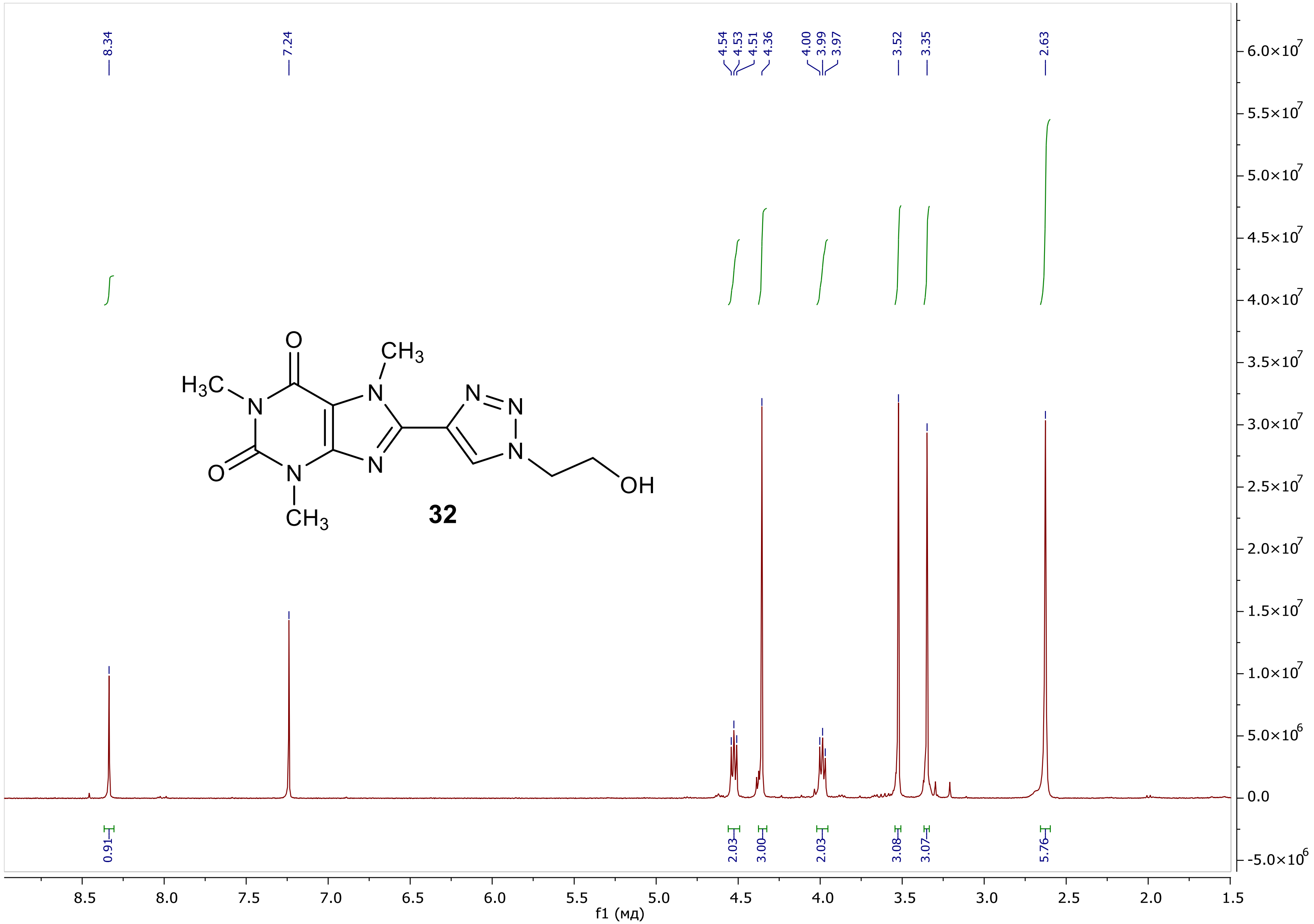

8-(1-(2-Hydroxyethyl)-1*H*-1,2,3-triazol-4-yl)-1,3,7-trimethyl-3,7-dihydro-1*H*-purine-2,6-dione (**32**) (^{13}C NMR, 126 MHz, $CDCl_3$+CD_3OD)

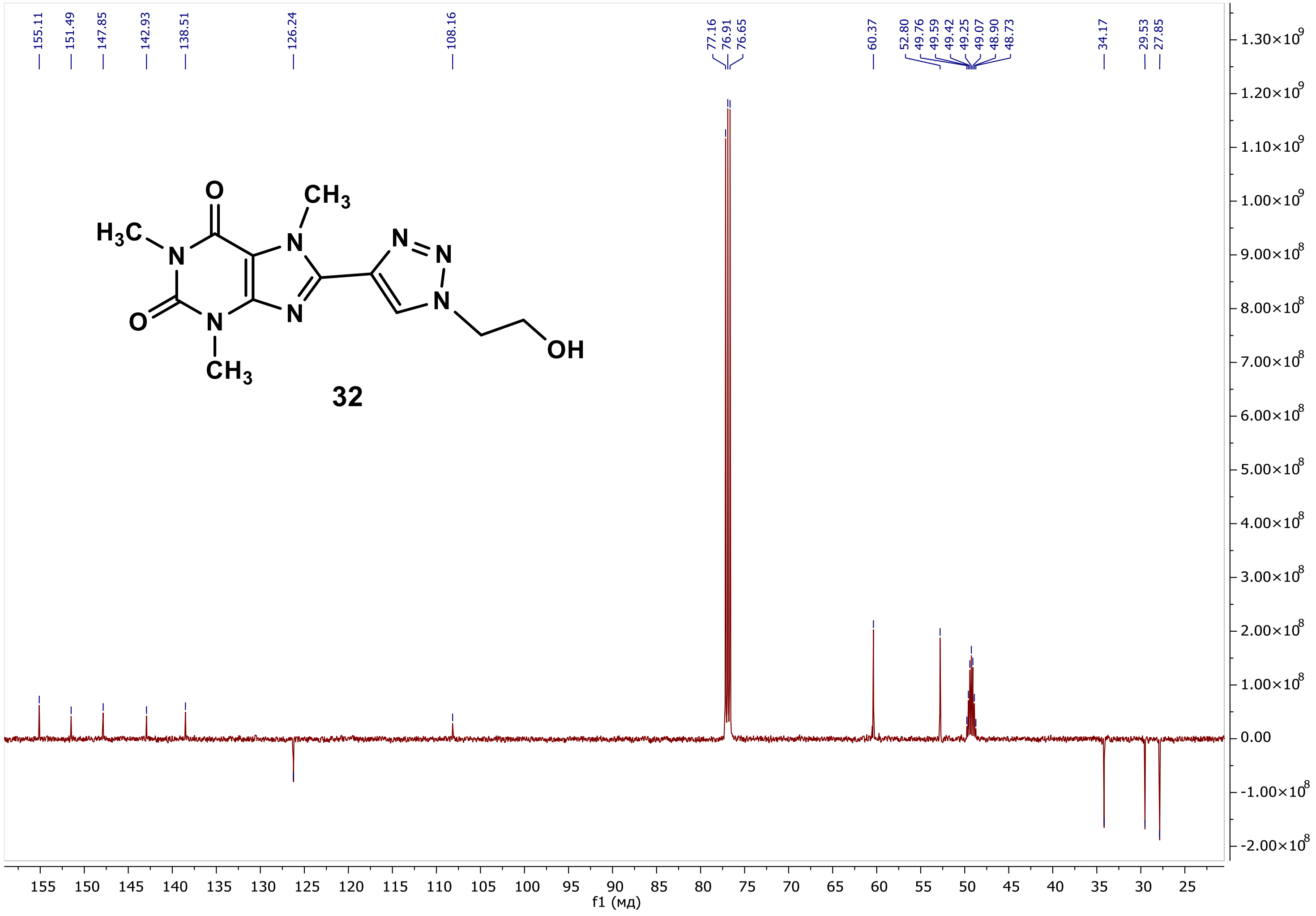

8-(1-(*tert*-Butyl)-1*H*-1,2,3-triazol-4-yl)-1,3,7-trimethyl-3,7-dihydro-1*H*-purine-2,6-dione (**33**) (^{1}H NMR, 400 MHz, $CDCl_3$)

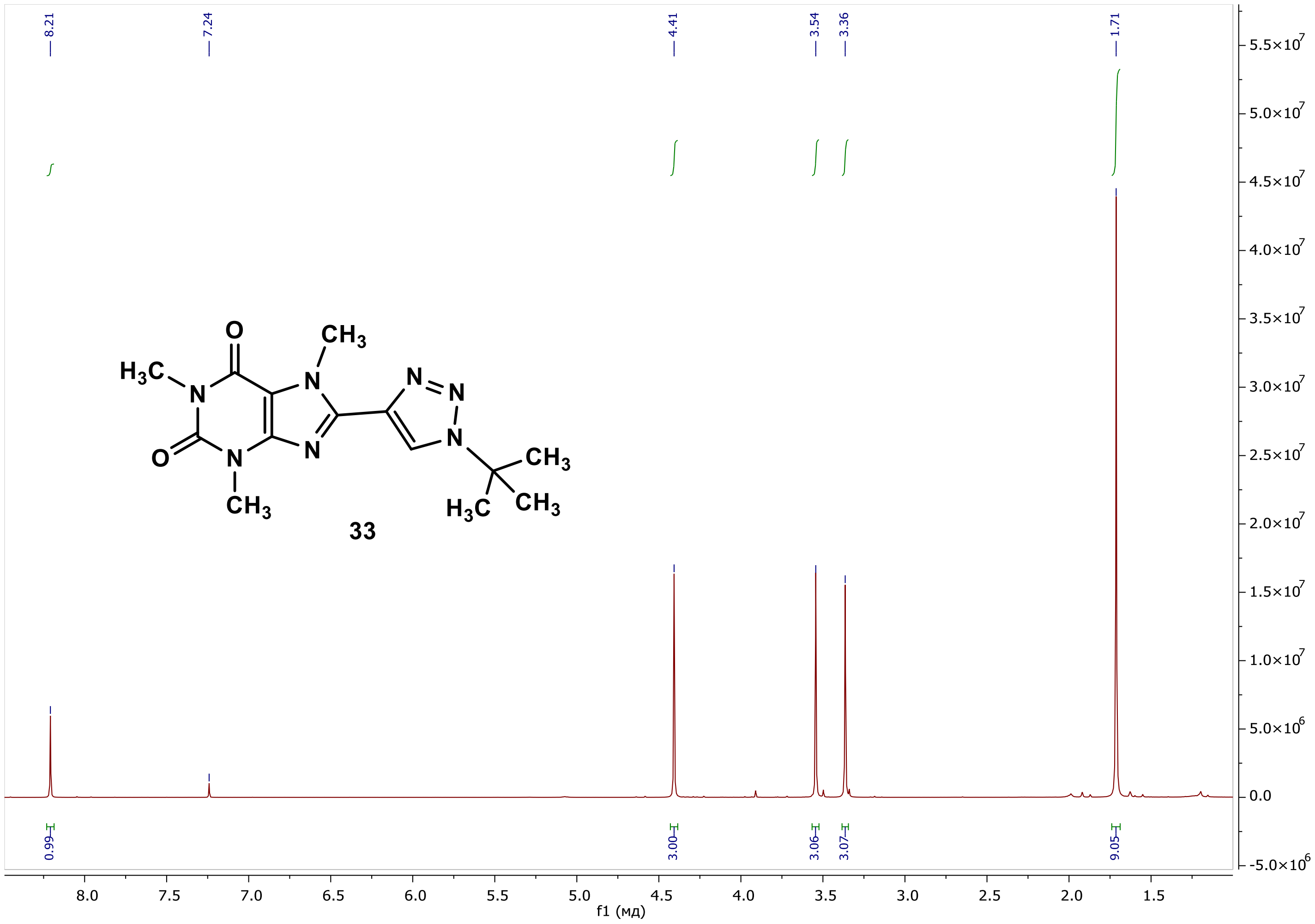

8-(1-(*tert*-Butyl)-1*H*-1,2,3-triazol-4-yl)-1,3,7-trimethyl-3,7-dihydro-1*H*-purine-2,6-dione (**33**) (^{13}C NMR, 101 MHz, $CDCl_3$)

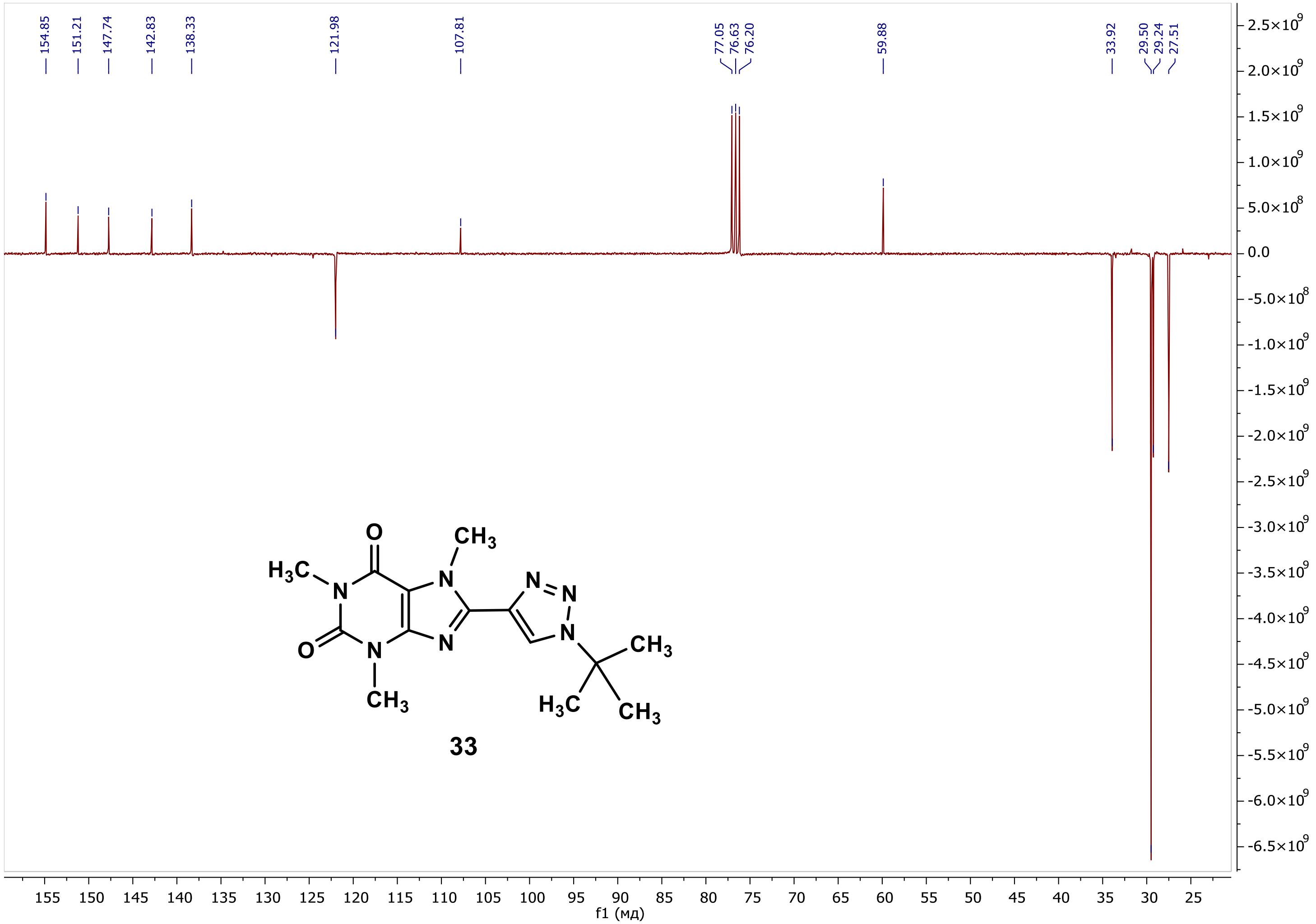

8-(3-(Diethylamino)prop-1-yn-1-yl)-1,3,7-trimethyl-3,7-dihydro-1*H*-purine-2,6-dione (**35**) (^{1}H NMR, 300 MHz, $CDCl_3$)

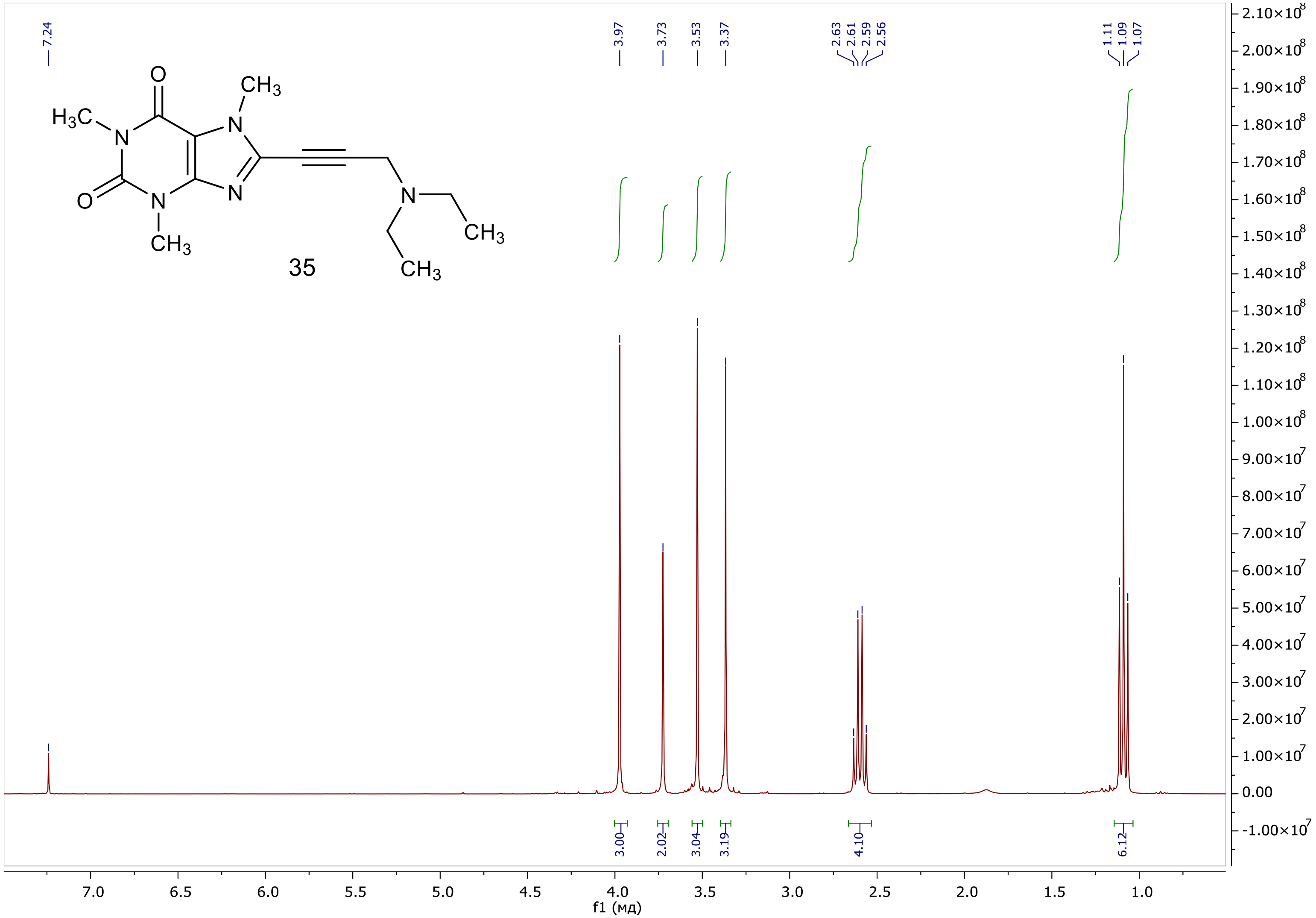

8-(3-(Diethylamino)prop-1-yn-1-yl)-1,3,7-trimethyl-3,7-dihydro-1*H*-purine-2,6- dione (**35**) (^{13}C NMR, 126 MHz, CDCl$_3$)

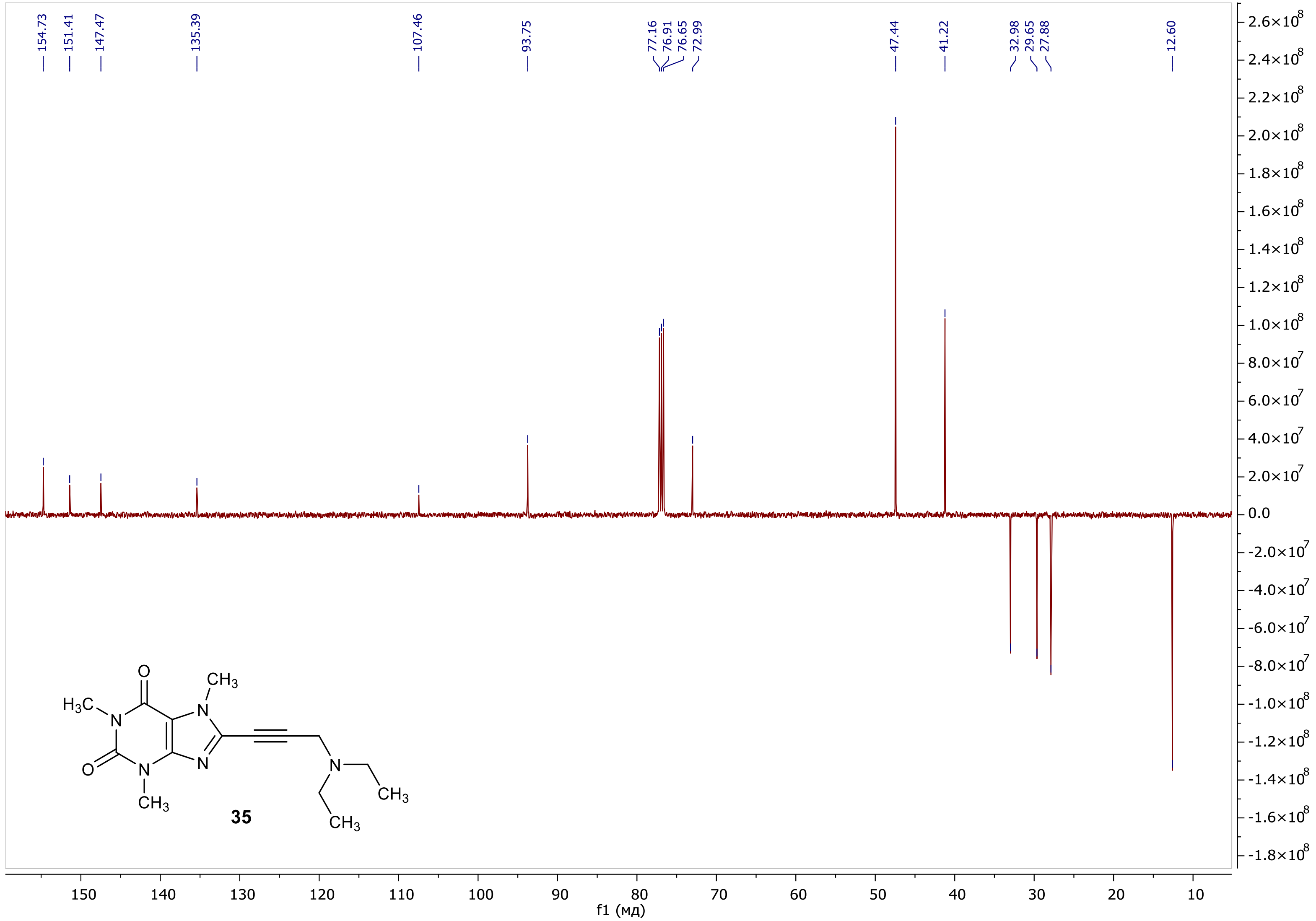

8-(3-(Diisopropylamino)prop-1-yn-1-yl)-1,3,7-trimethyl-3,7-dihydro-1*H*-purine- 2,6-dione (**39**) (^{1}H NMR, 400 MHz, $CDCl_3$)

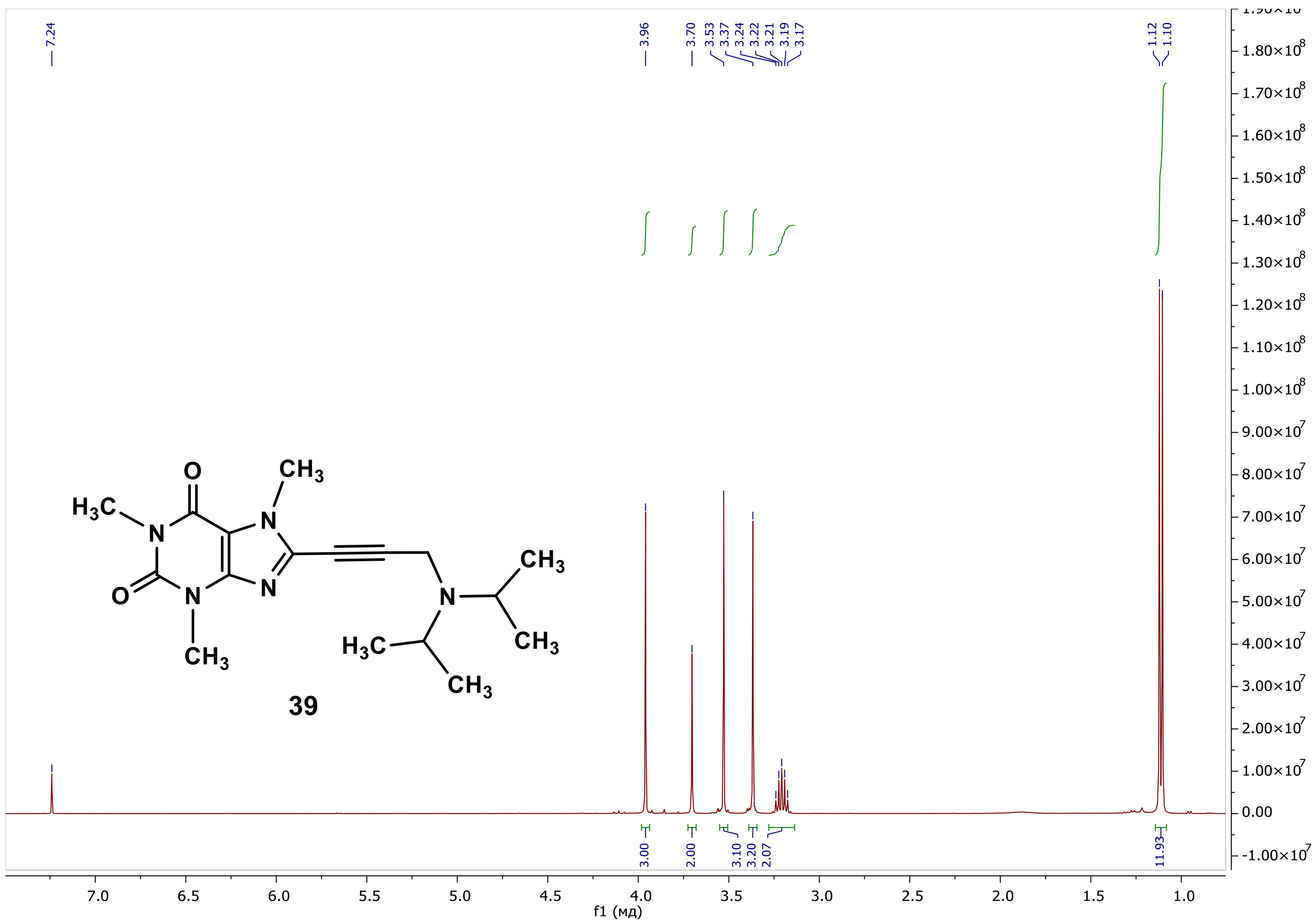

8-(3-(Diisopropylamino)prop-1-yn-1-yl)-1,3,7-trimethyl-3,7-dihydro-1*H*-purine-2,6-dione (**39**) (^{13}C NMR, 101 MHz, $CDCl_3$)

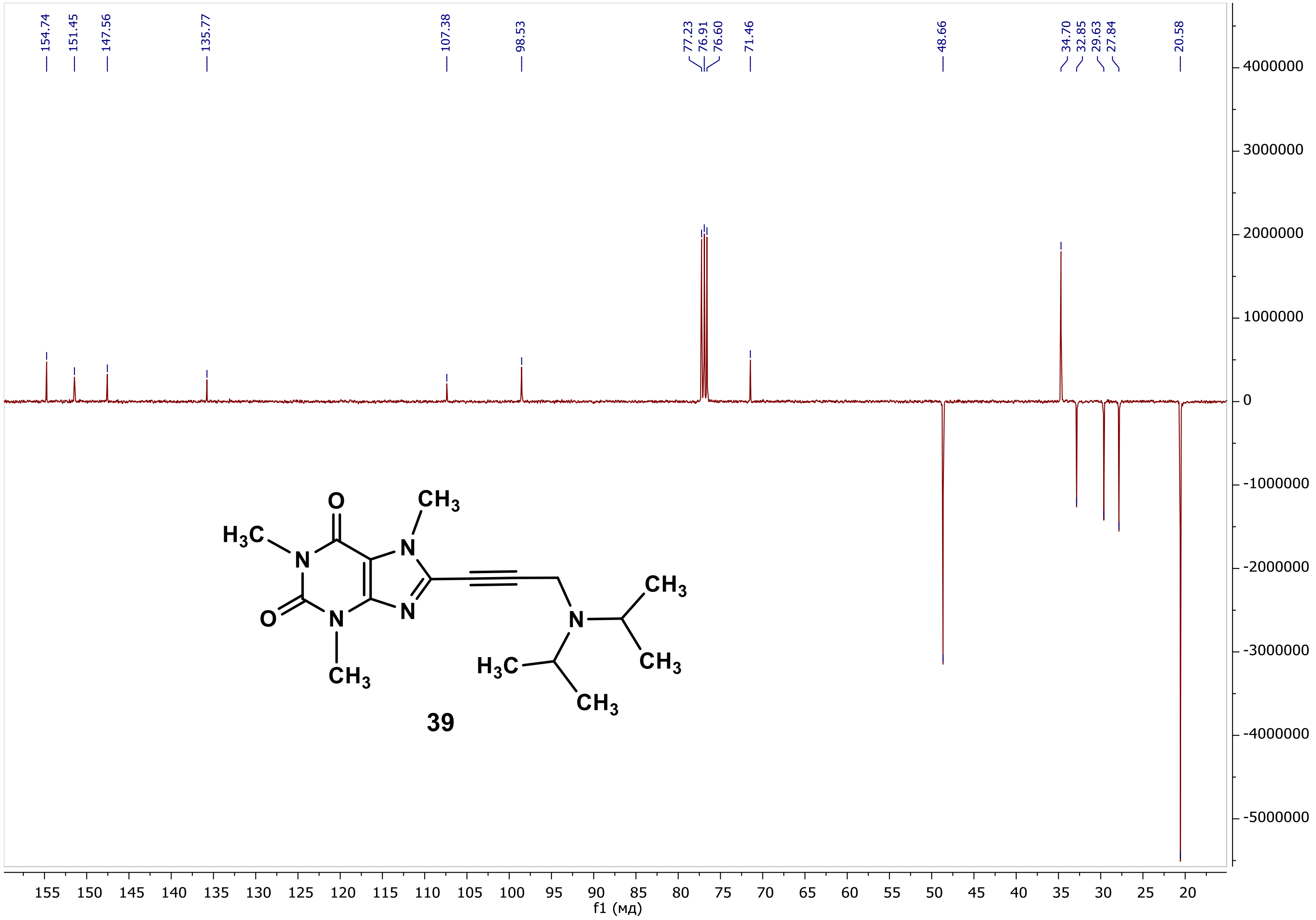

8-(3-(Dibutylamino)prop-1-yn-1-yl)-1,3,7-trimethyl-3,7-dihydro-1*H*-purine-2,6-dione (**40**) (^{1}H NMR, 300 MHz, $CDCl_3$)

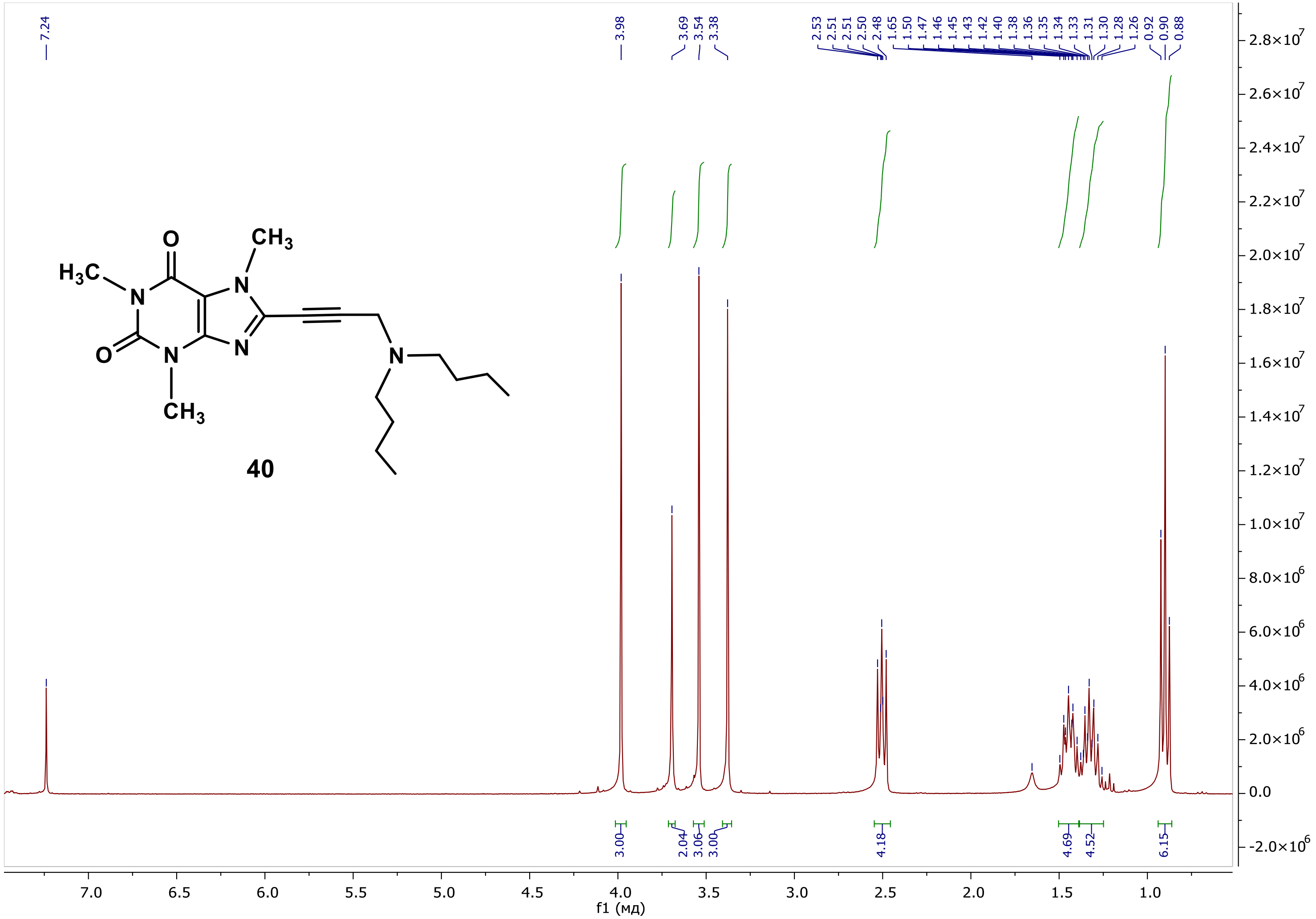

8-(3-(Dibutylamino)prop-1-yn-1-yl)-1,3,7-trimethyl-3,7-dihydro-1*H*-purine-2,6-dione (**40**) (^{13}C NMR, 126 MHz, $CDCl_3$)

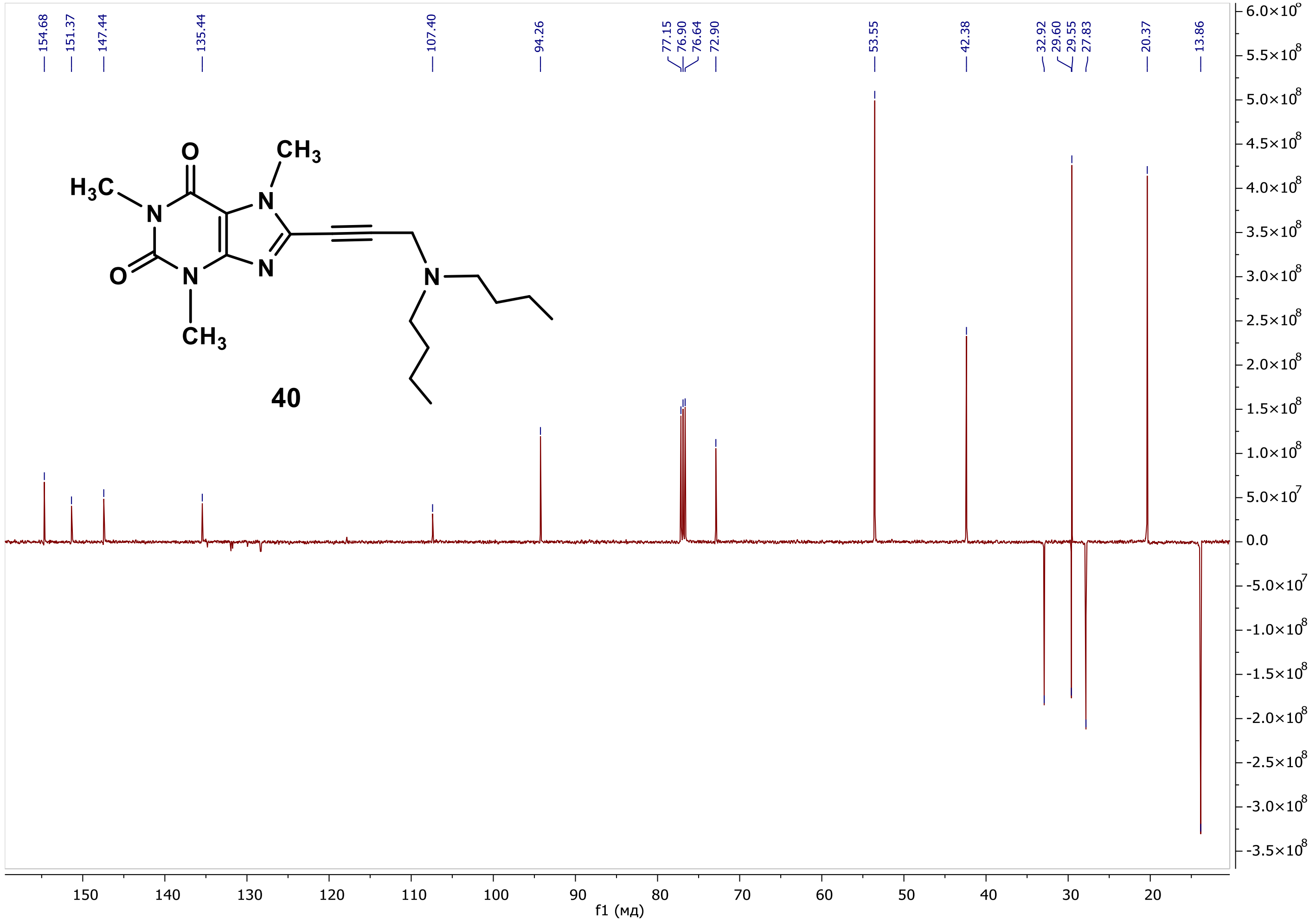

8-(3-(Dicyclohexylamino)prop-1-yn-1-yl)-1,3,7-trimethyl-3,7-dihydro-1*H*-purine-2,6-dione (**41**) (^{1}H NMR, 400 MHz, CDCl₃)

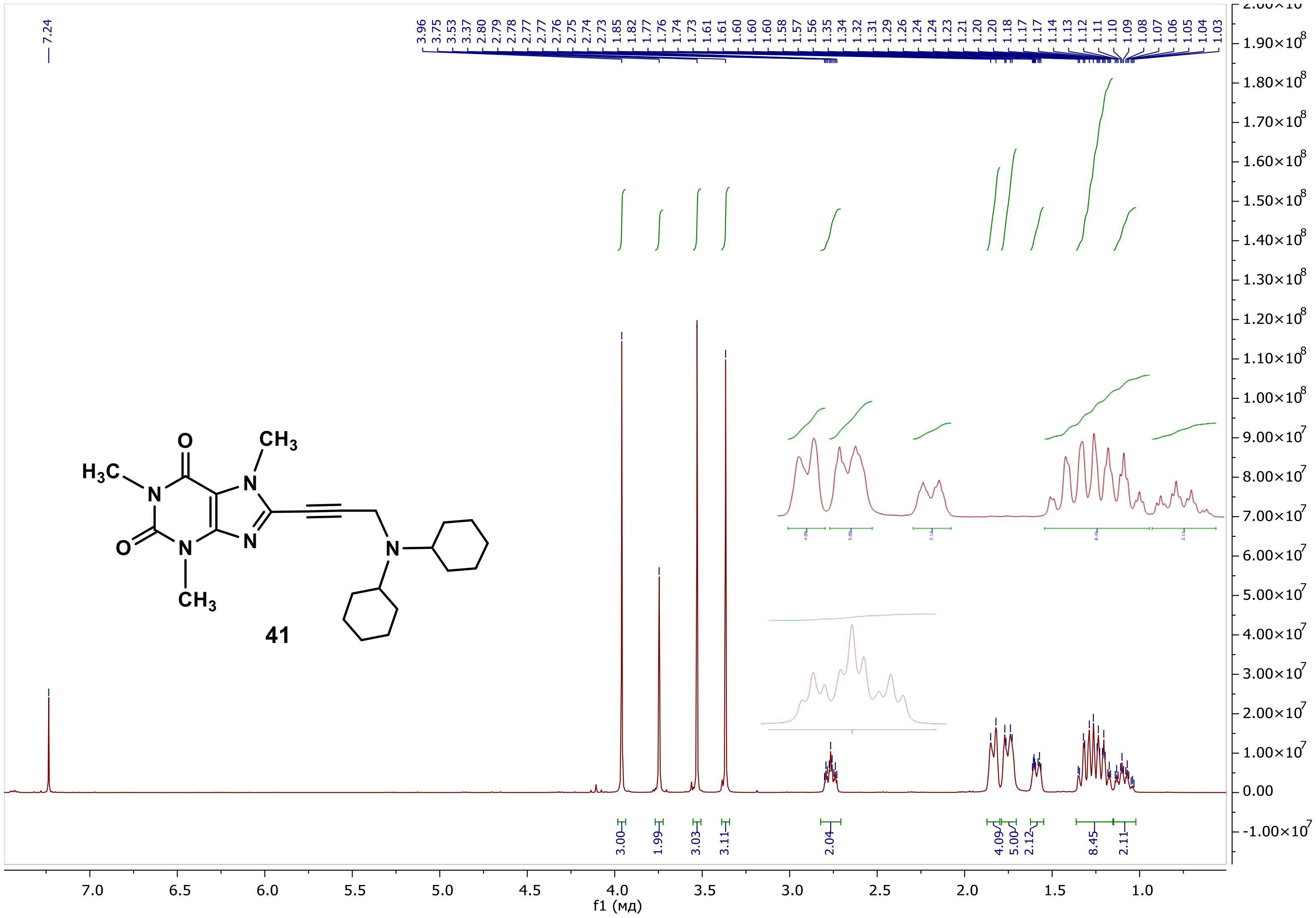

8-(3-(Dicyclohexylamino)prop-1-yn-1-yl)-1,3,7-trimethyl-3,7-dihydro-1*H*-purine-2,6-dione (**41**) (^{13}C NMR, 101 MHz, $CDCl_3$)

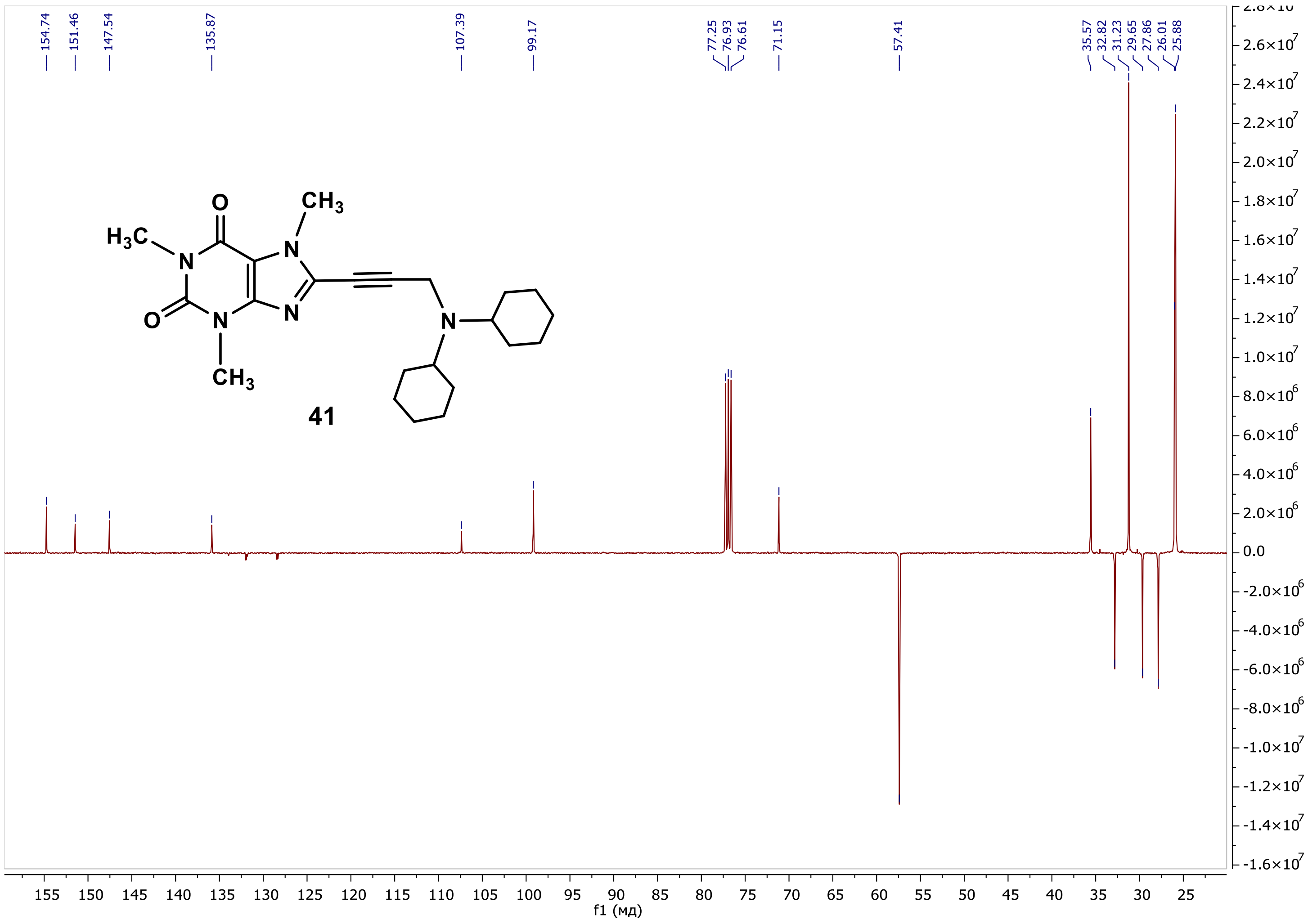

1,3,7-Trimethyl-8-(3-(pyrrolidin-1-yl)prop-1-yn-1-yl)-3,7-dihydro-1*H*-purine-2,6-dione (**51**) (^{1}H NMR, 400 MHz, $CDCl_3$)

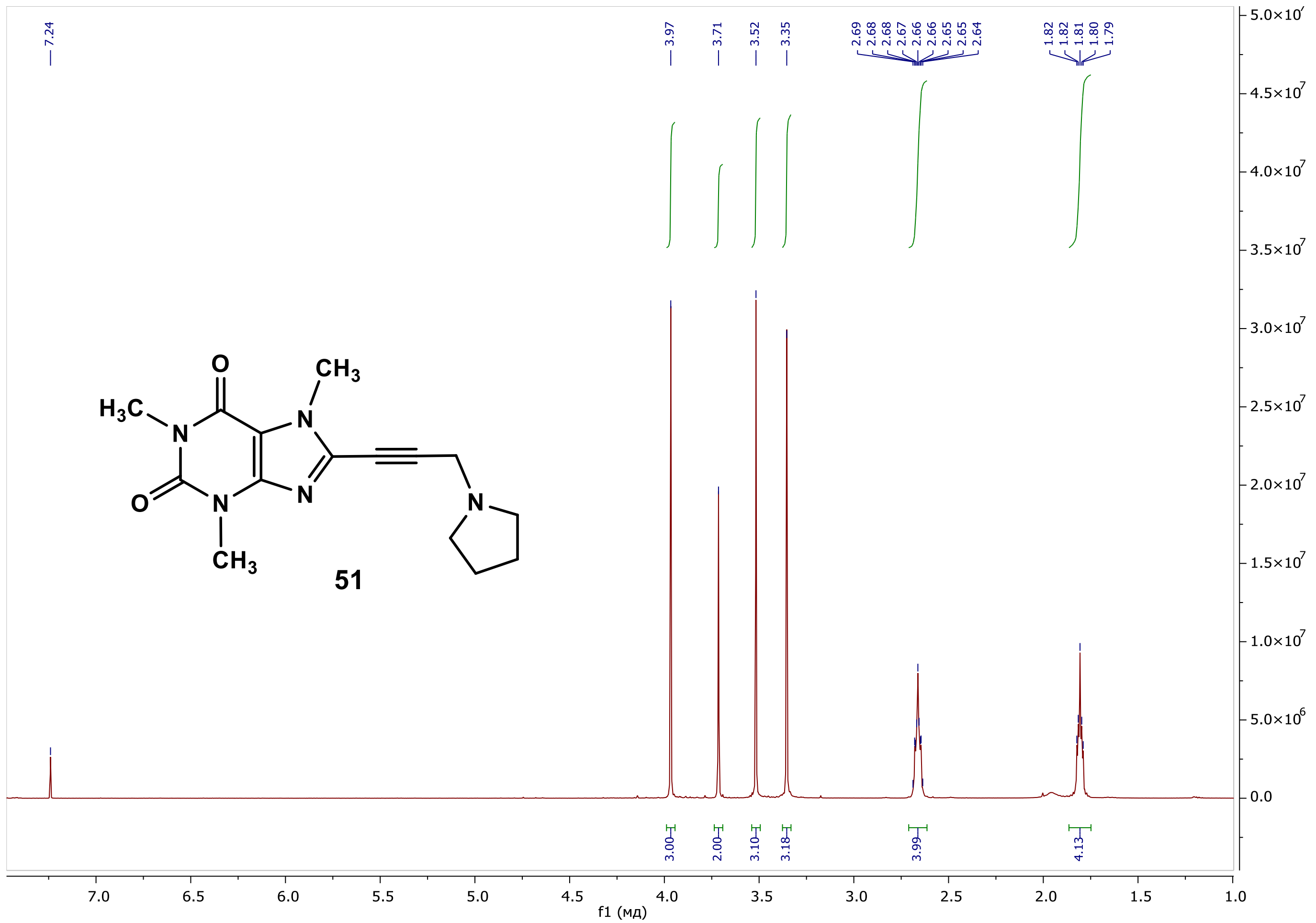

1,3,7-Trimethyl-8-(3-(pyrrolidin-1-yl)prop-1-yn-1-yl)-3,7-dihydro-1*H*-purine-2,6- dione (**51**) (^{13}C NMR, 101 MHz, $CDCl_3$)

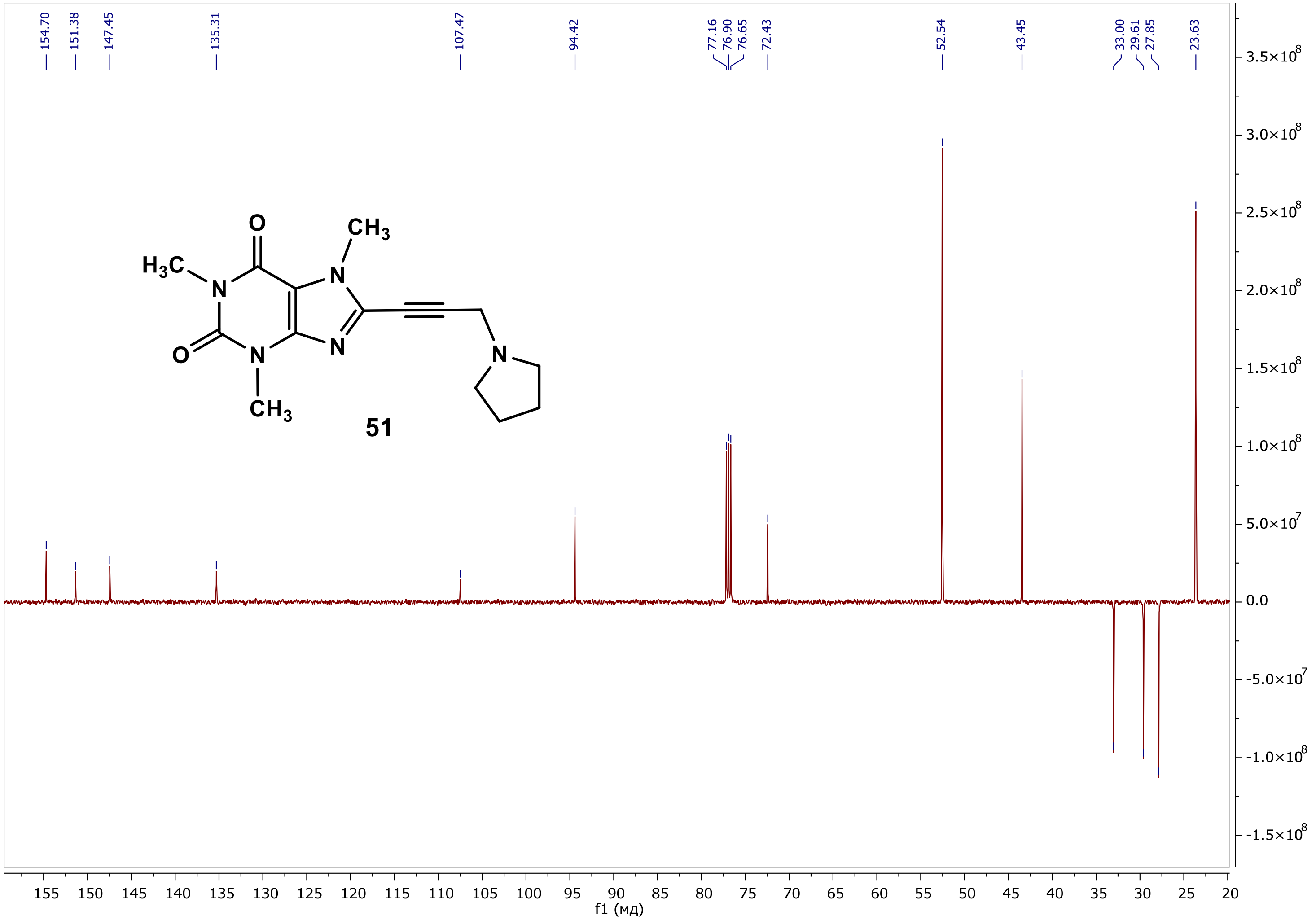

8-(3-(Azepan-1-yl)prop-1-yn-1-yl)-1,3,7-trimethyl-3,7-dihydro-1*H*-purine-2,6-dione (**52**) (^{1}H NMR, 400 MHz, $CDCl_3$)

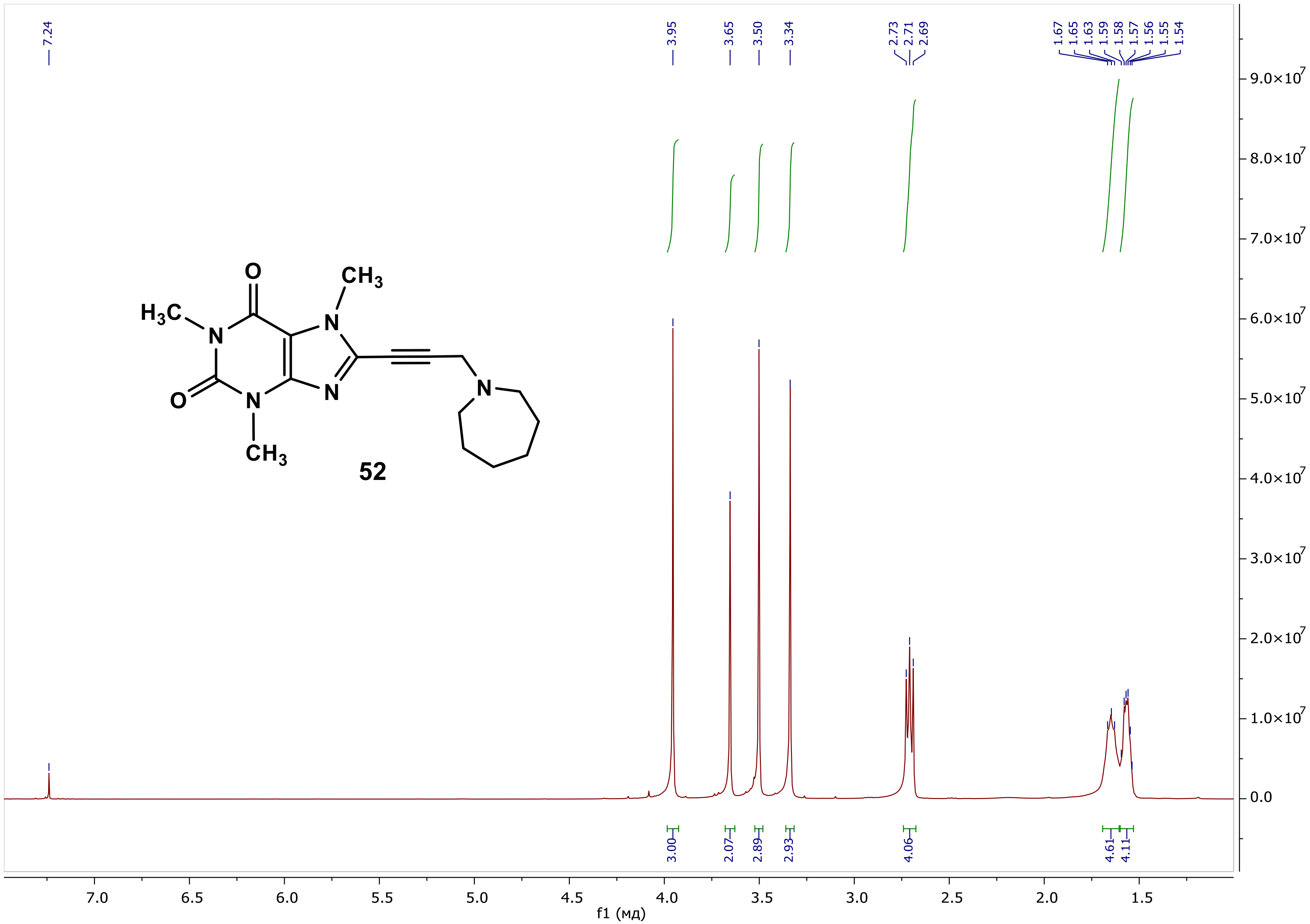

8-(3-(Azepan-1-yl)prop-1-yn-1-yl)-1,3,7-trimethyl-3,7-dihydro-1*H*-purine-2,6-dione (**52**) (^{13}C NMR, 101 MHz, $CDCl_3$)

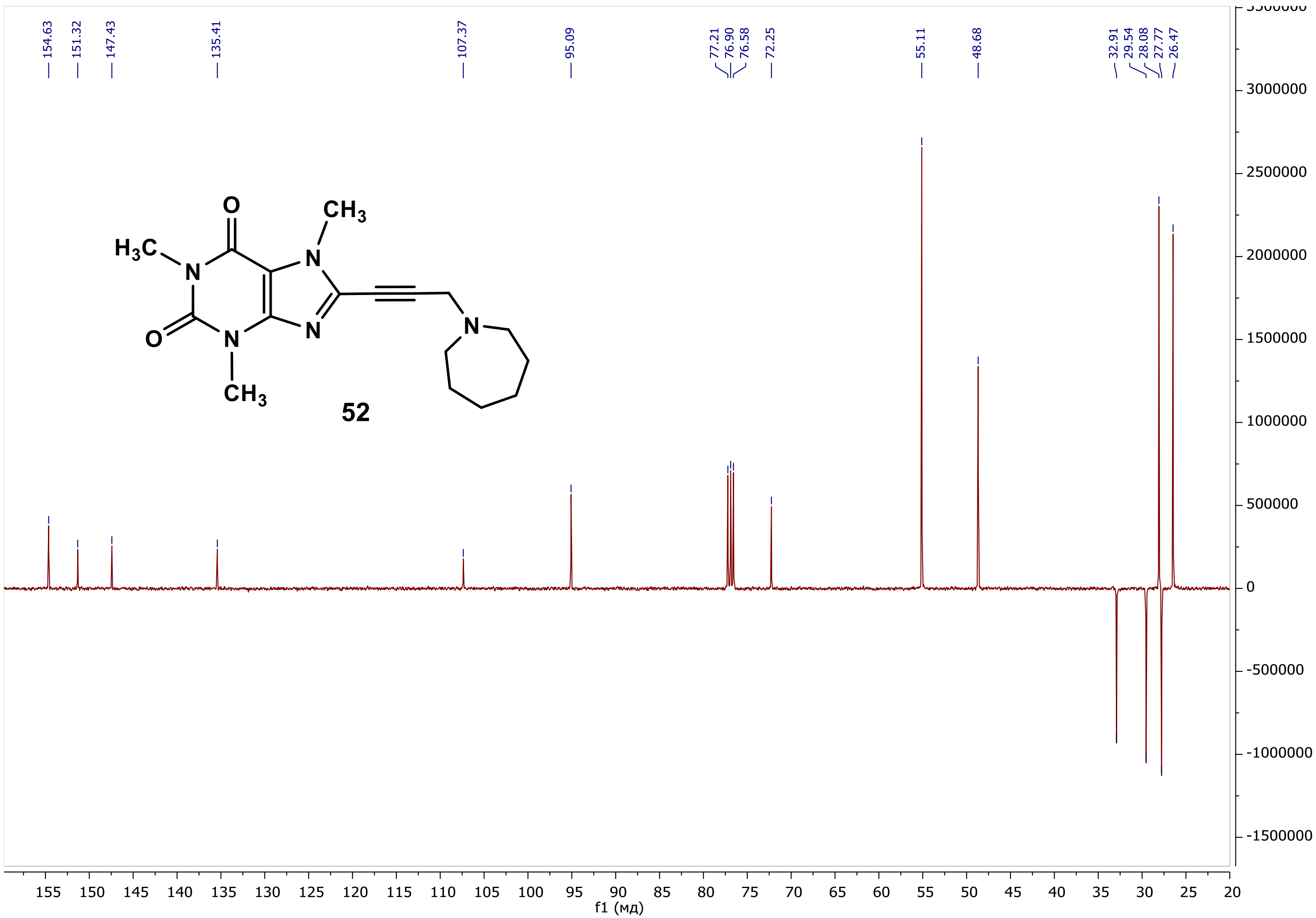

8-(3-(Azocan-1-yl)prop-1-yn-1-yl)-1,3,7-trimethyl-3,7-dihydro-1*H*-purine-2,6-dione (**53**) (^{1}H NMR, 400 MHz, $CDCl_3$)

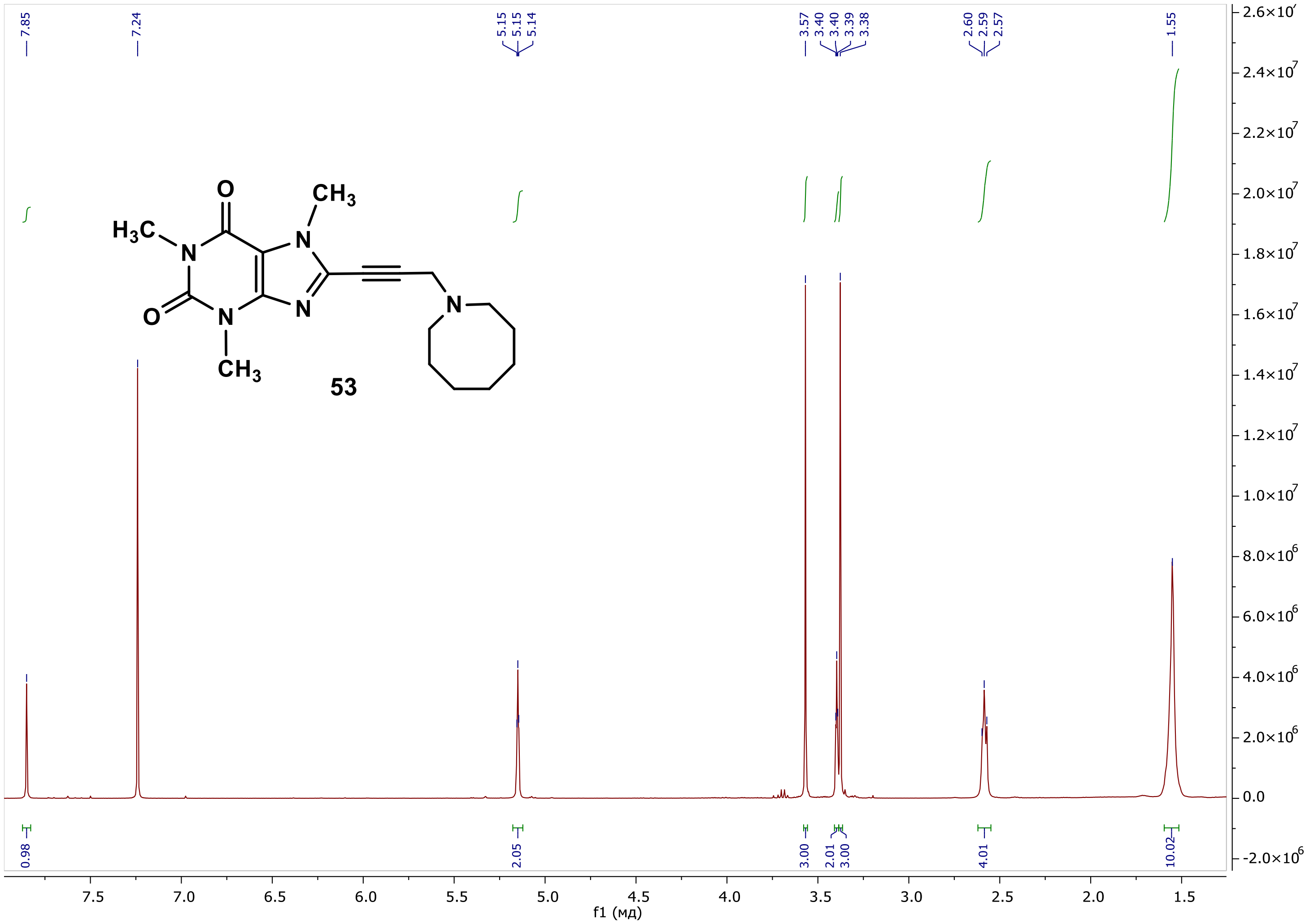

8-(3-(Azocan-1-yl)prop-1-yn-1-yl)-1,3,7-trimethyl-3,7-dihydro-1*H*-purine-2,6-dione (**53**) (^{13}C NMR, 126 MHz, $CDCl_3$)

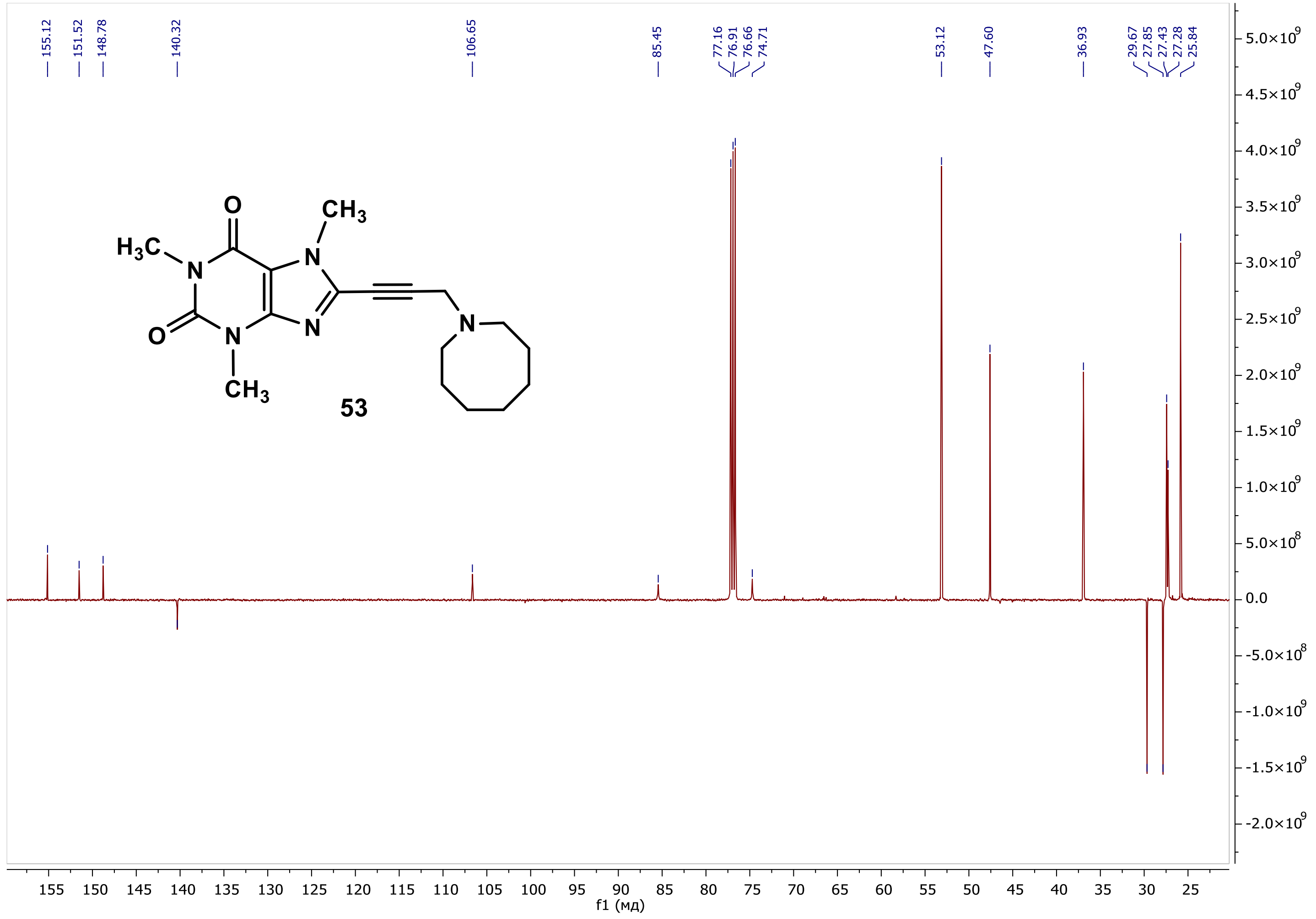

8-(3-(1,4-Oxazepan-4-yl)prop-1-yn-1-yl)-1,3,7-trimethyl-3,7-dihydro-1*H*-purine-2,6-dione (**54**) (^{1}H NMR, 400 MHz, CDCl$_3$)

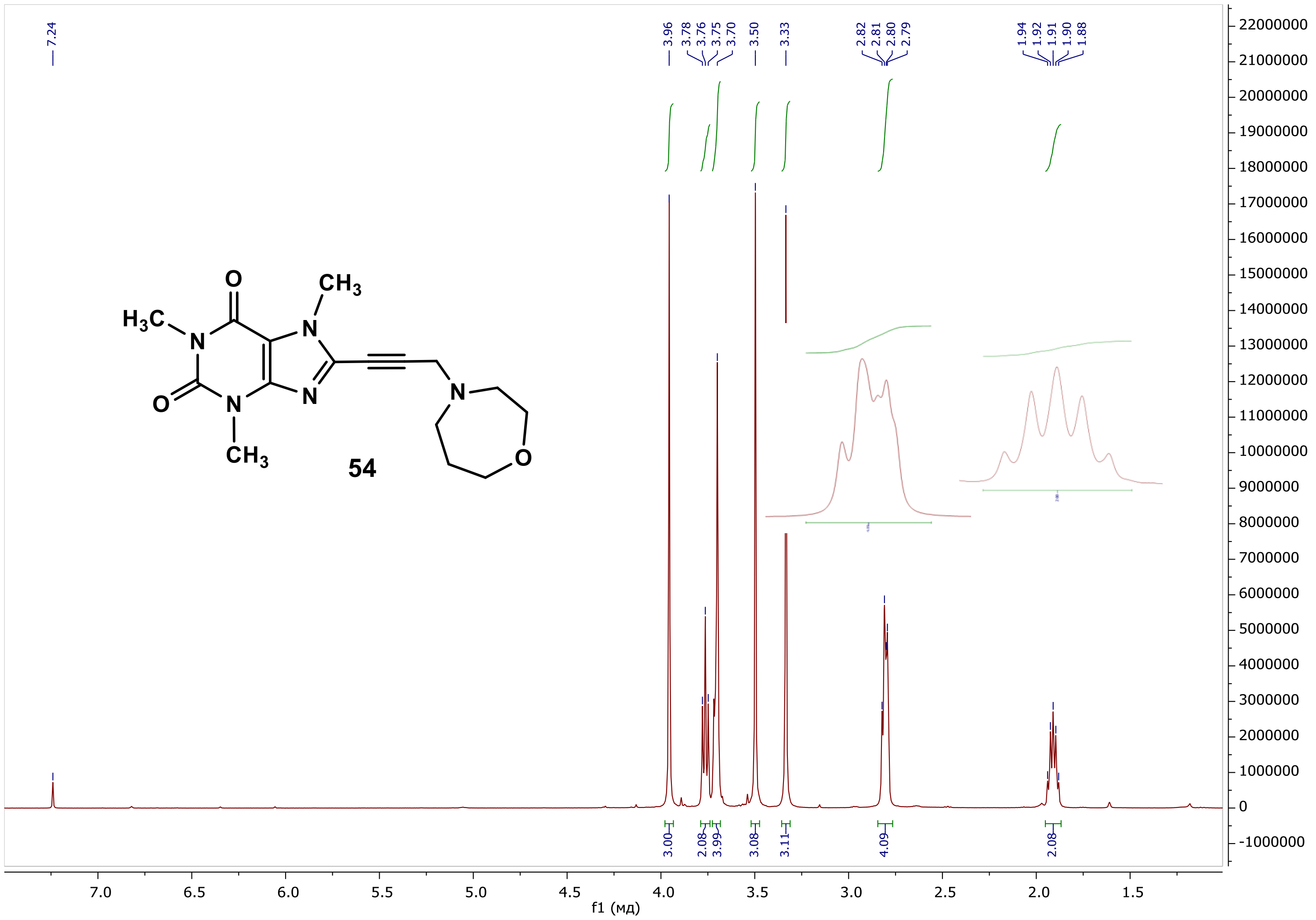

8-(3-(1,4-Oxazepan-4-yl)prop-1-yn-1-yl)-1,3,7-trimethyl-3,7-dihydro-1*H*-purine-2,6-dione (**54**) (^{13}C NMR, 126 MHz, $CDCl_3$)

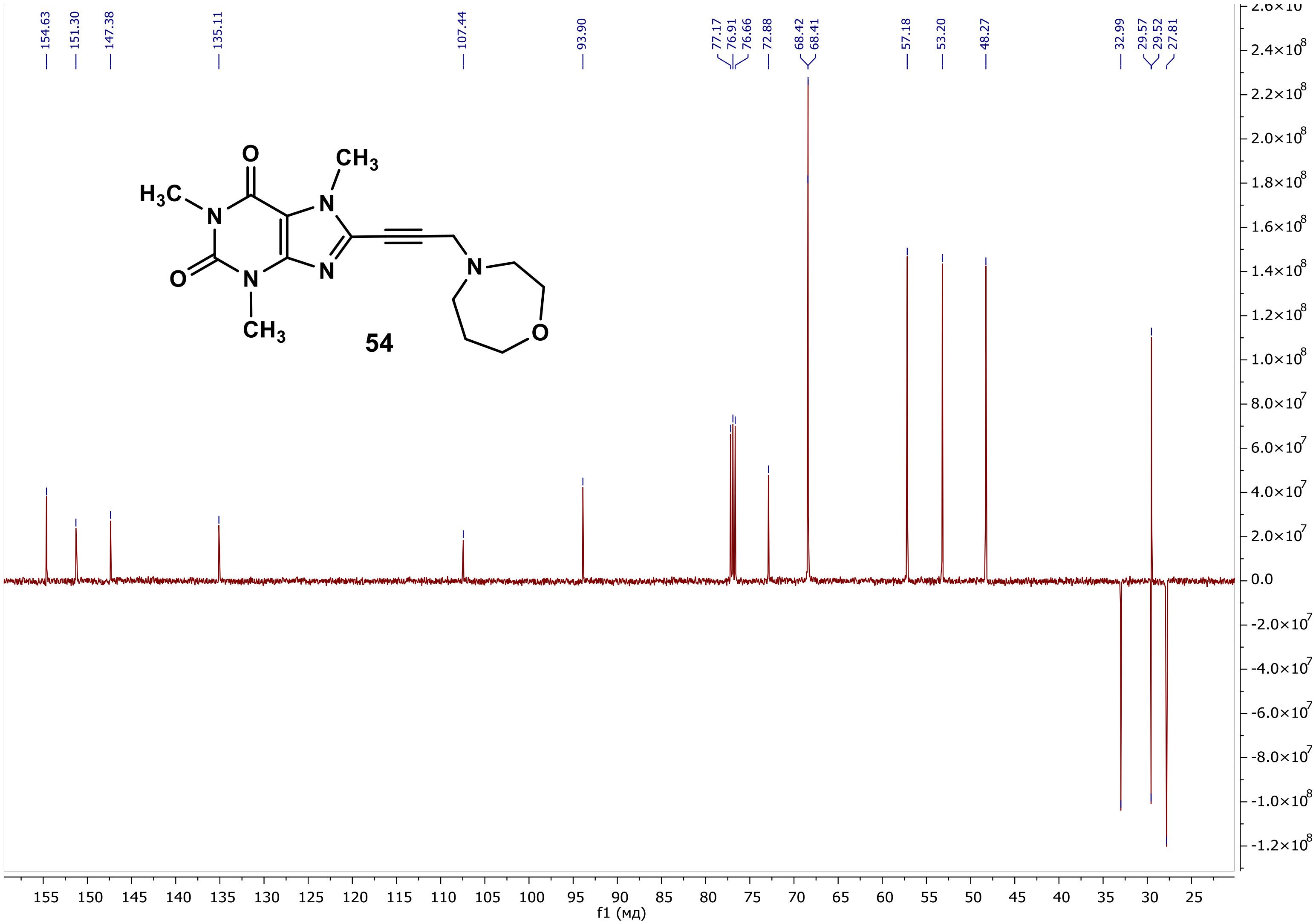

1,3,7-Trimethyl-8-(3-(4-methylpiperidin-1-yl)prop-1-yn-1-yl)-3,7-dihydro-1*H*-purine-2,6-dione (**55**) (^{1}H NMR, 500 MHz, $CDCl_3$)

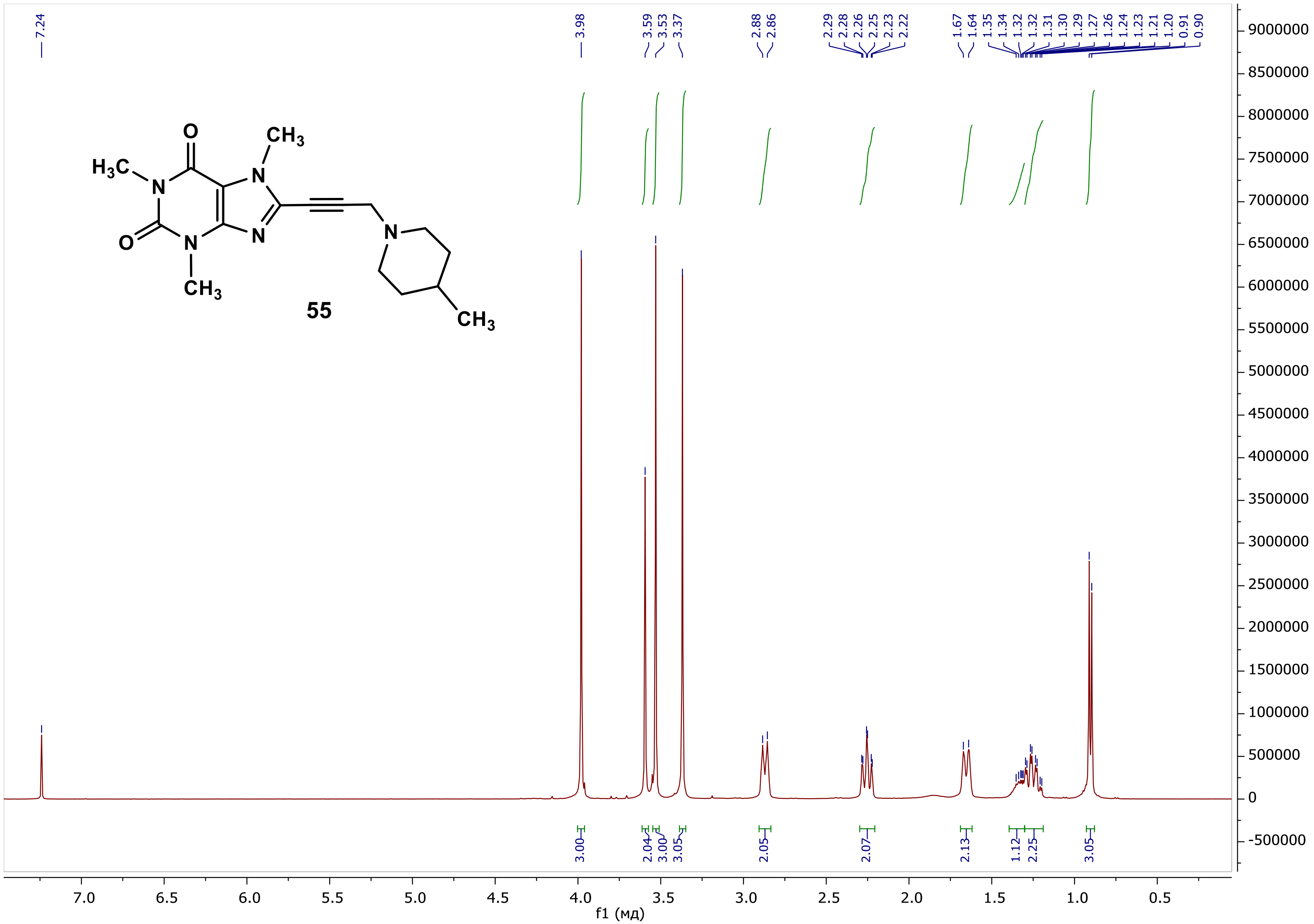

1,3,7-Trimethyl-8-(3-(4-methylpiperidin-1-yl)prop-1-yn-1-yl)-3,7-dihydro-1*H*-purine-2,6-dione (**55**) (^{13}C NMR, 126 MHz, $CDCl_3$)

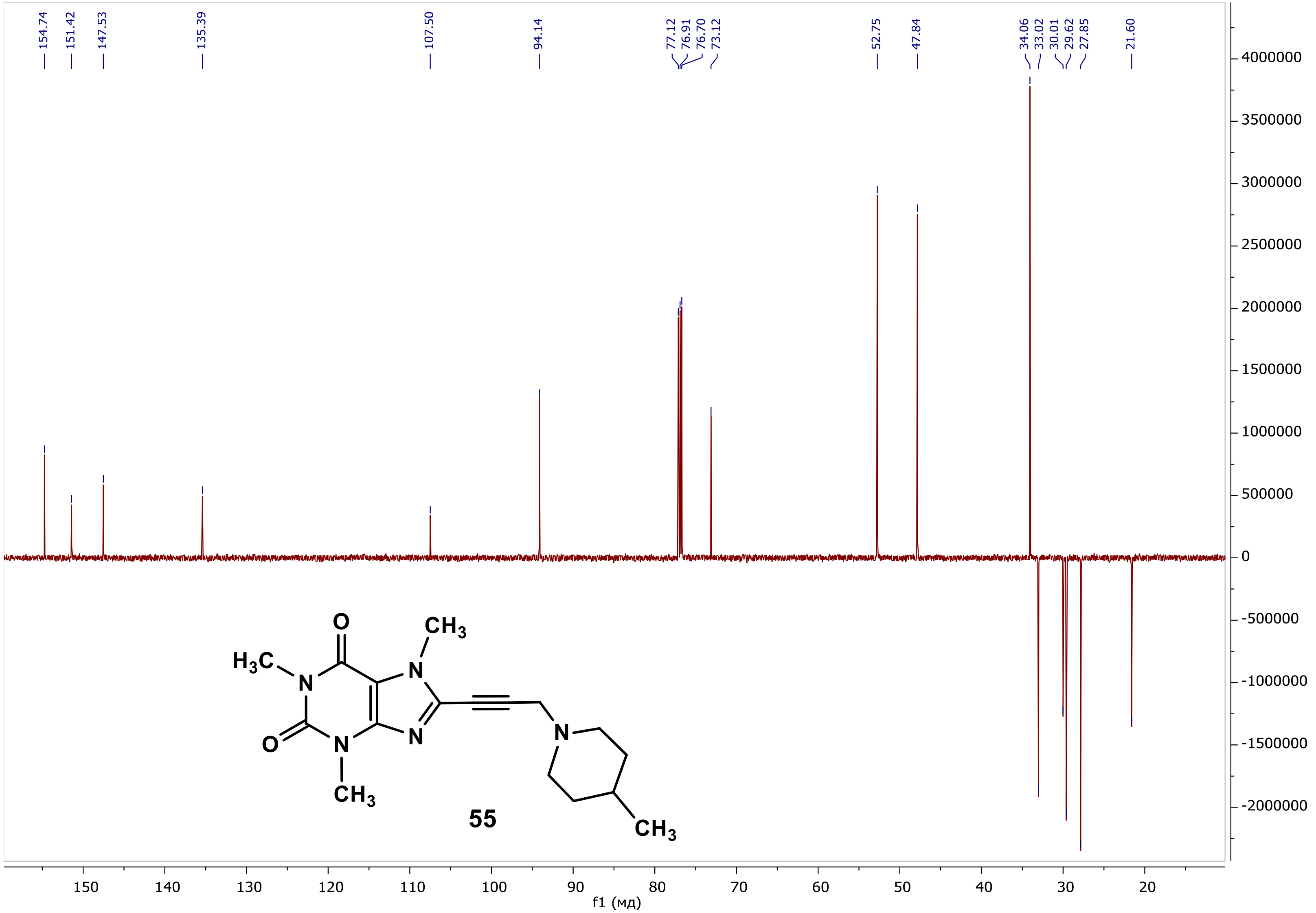

1,3,7-Trimethyl-8-(3-morpholinoprop-1-yn-1-yl)-3,7-dihydro-1*H*-purine-2,6-dione (**56**) (^{1}H NMR, 400 MHz, $CDCl_3$)

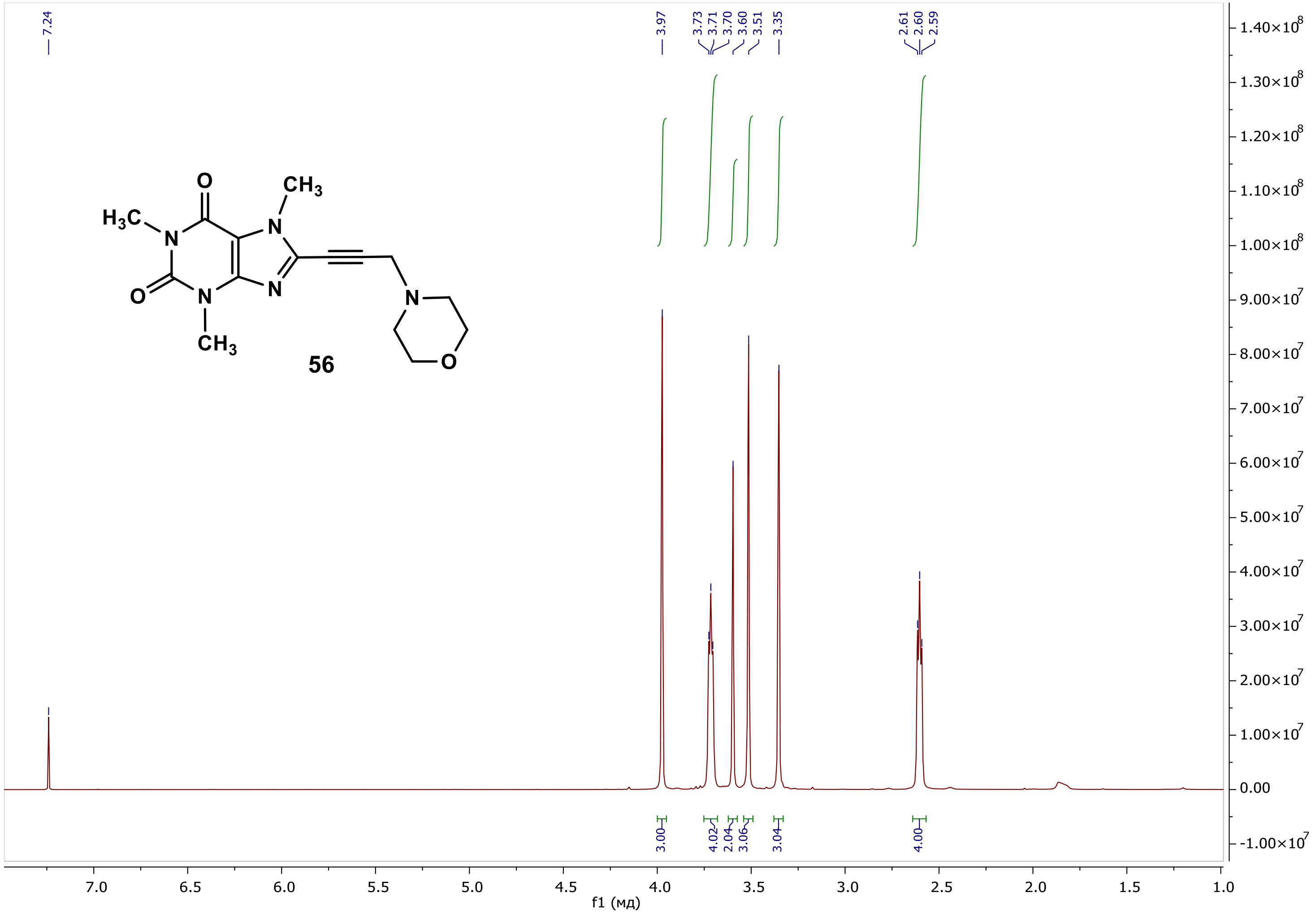

1,3,7-Trimethyl-8-(3-morpholinoprop-1-yn-1-yl)-3,7-dihydro-1*H*-purine-2,6-dione (**56**) (^{13}C NMR, 126 MHz, $CDCl_3$)

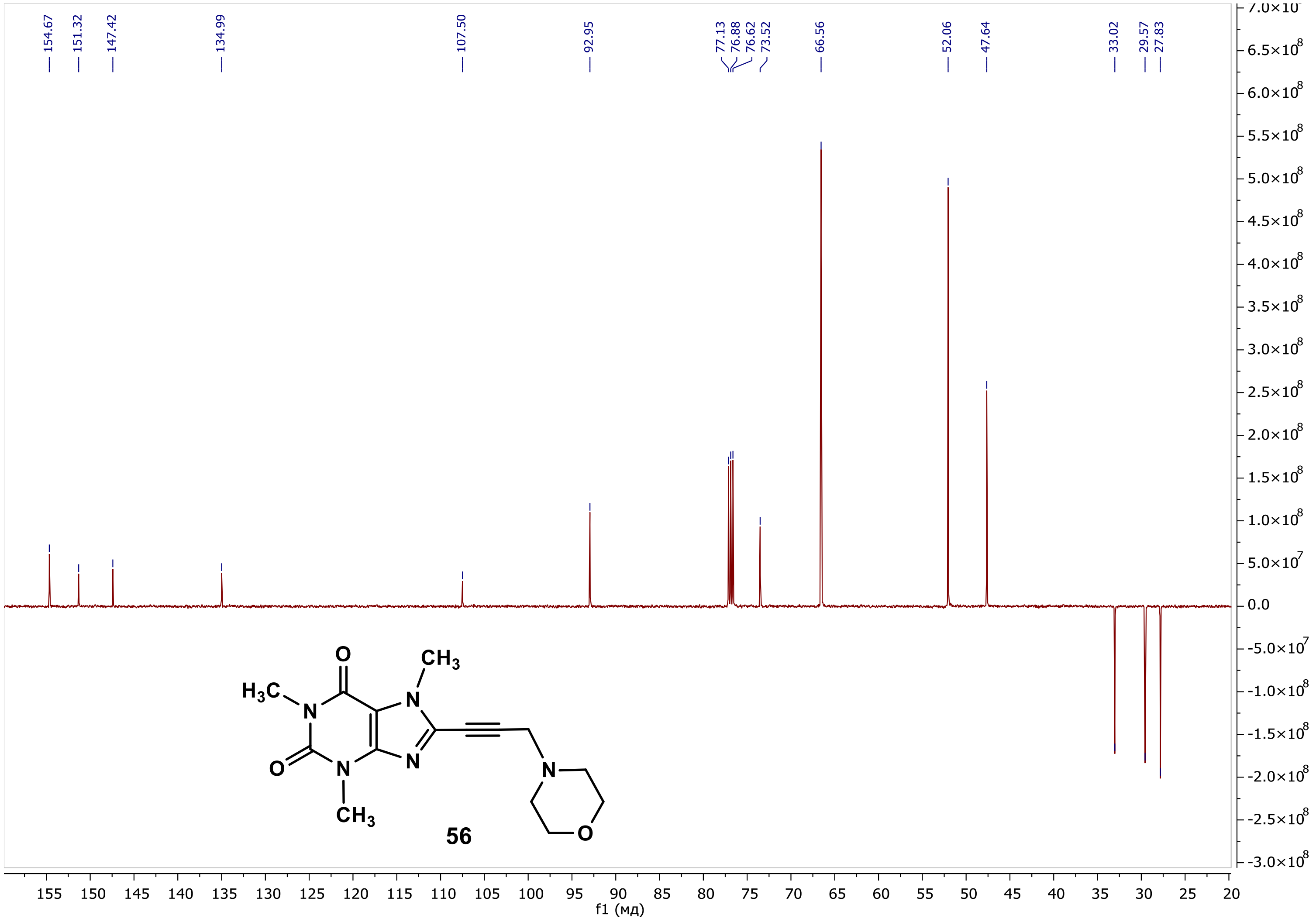

1,3,7-Trimethyl-8-(3-(4-methylpiperazin-1-yl)prop-1-yn-1-yl)-3,7-dihydro-1*H*-purine-2,6-dione (**57**) (^{1}H NMR, 400 MHz, $CDCl_3$)

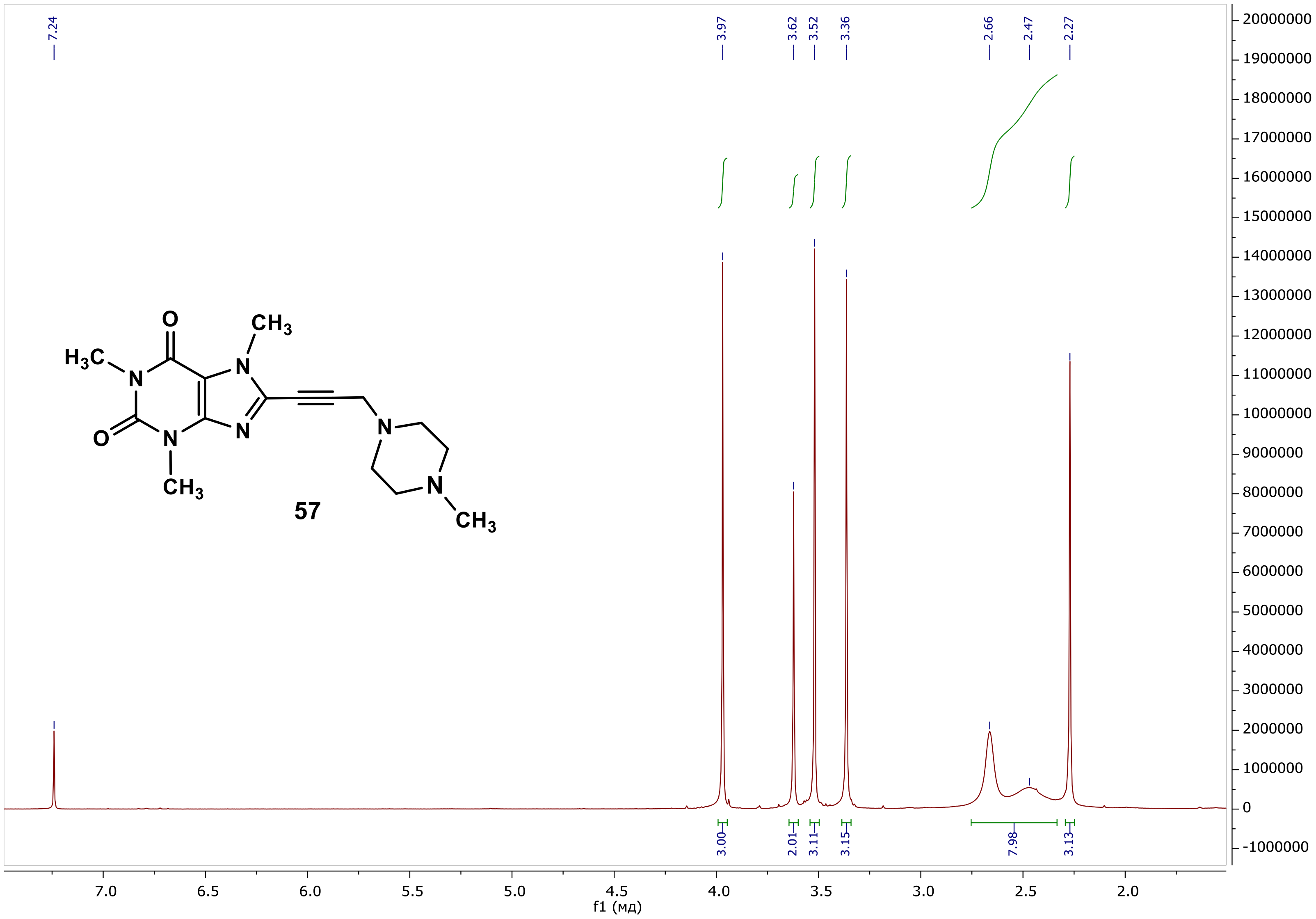

1,3,7-Trimethyl-8-(3-(4-methylpiperazin-1-yl)prop-1-yn-1-yl)-3,7-dihydro-1*H*-purine-2,6-dione (**57**) (^{13}C NMR, 126 MHz, $CDCl_3$)

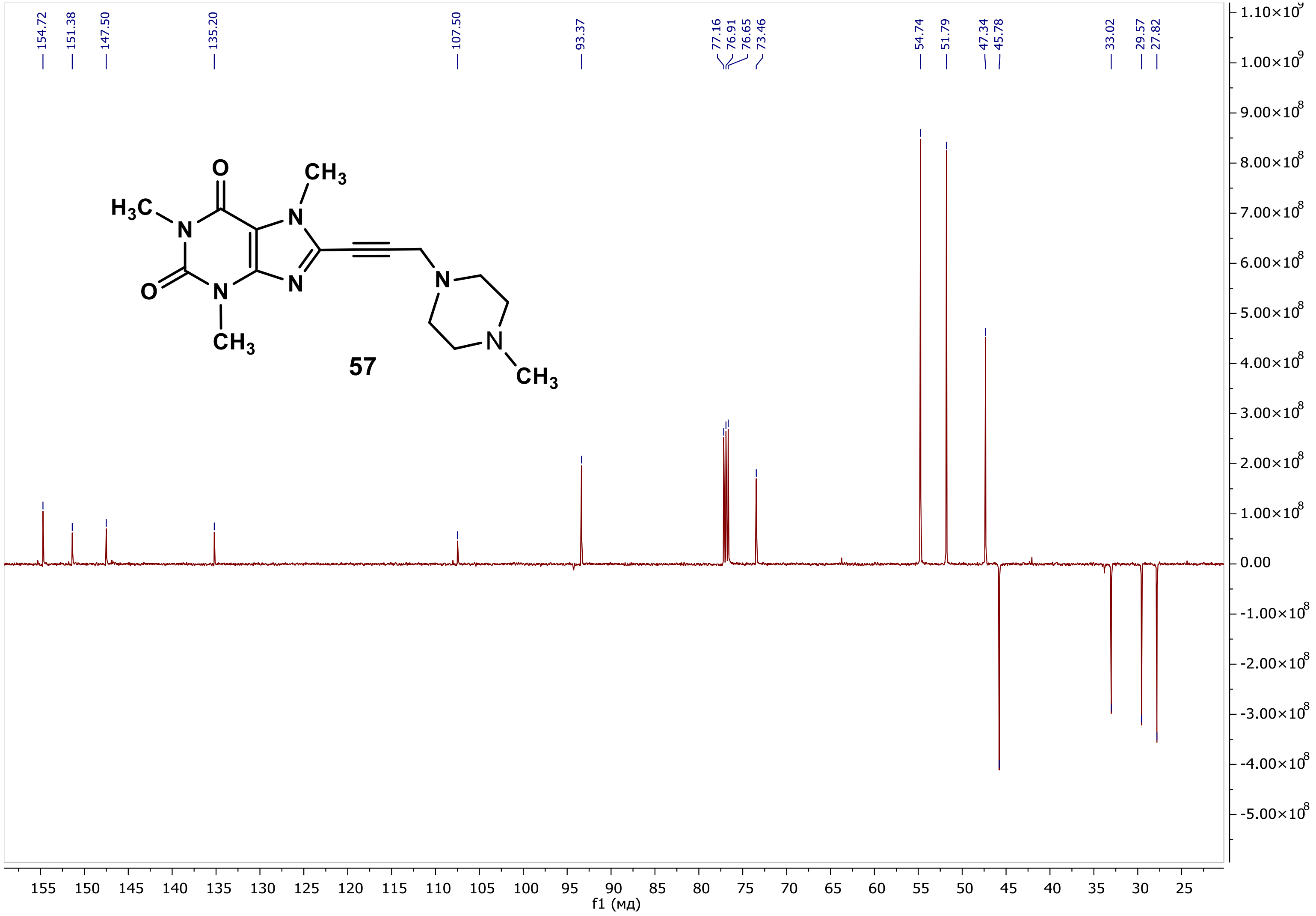

tert-Butyl 4-(3-(1,3,7-trimethyl-2,6-dioxo-3,7-dihydro-1*H*-purin-8-yl)prop-2-yn-1-yl)piperazine-1-carboxylate (**58**) (^{1}H NMR, 400 MHz, $CDCl_3$)

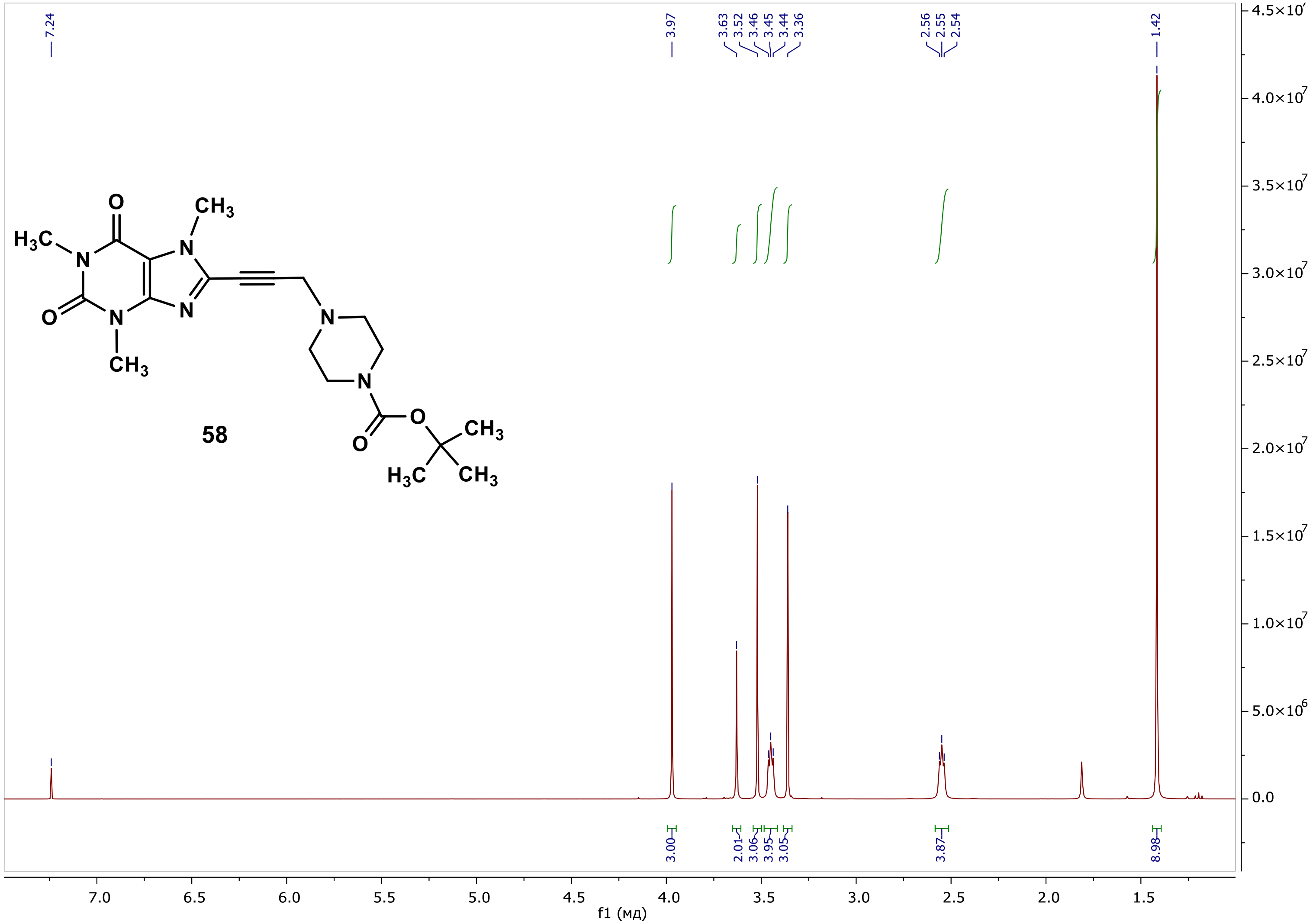

tert-Butyl 4-(3-(1,3,7-trimethyl-2,6-dioxo-3,7-dihydro-1*H*-purin-8-yl)prop-2-yn-1-yl)piperazine-1-carboxylate (**58**) (^{13}C NMR, 101 MHz, $CDCl_3$)

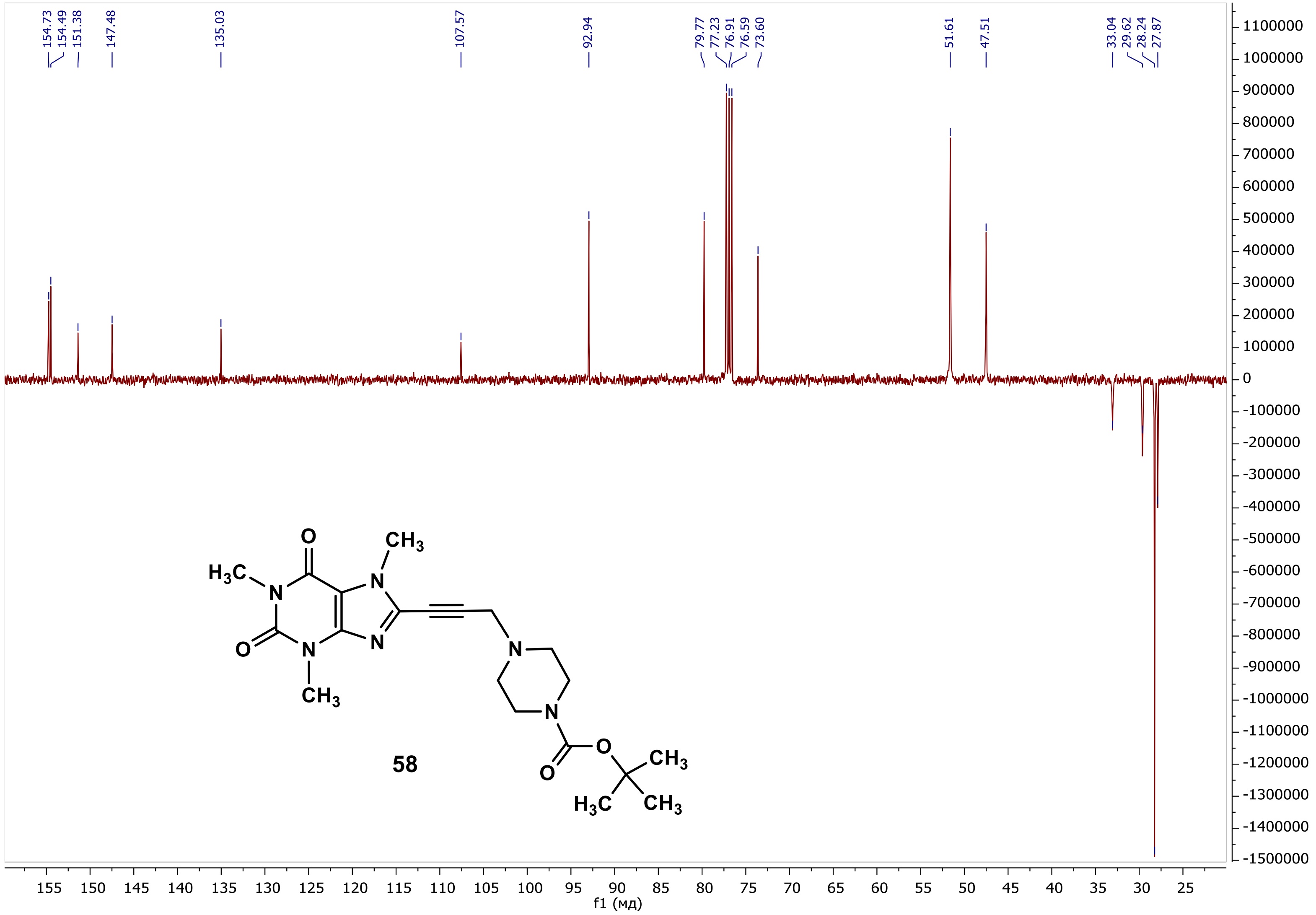

1,3,7-Trimethyl-8-(3-(4-(2-(pyrrolidin-1-yl)ethyl)piperazin-1-yl)prop-1-yn-1-yl)-3,7-dihydro-1*H*-purine-2,6-dione hydrate (**59**) (^{1}H NMR, 300 MHz, $CDCl_3$)

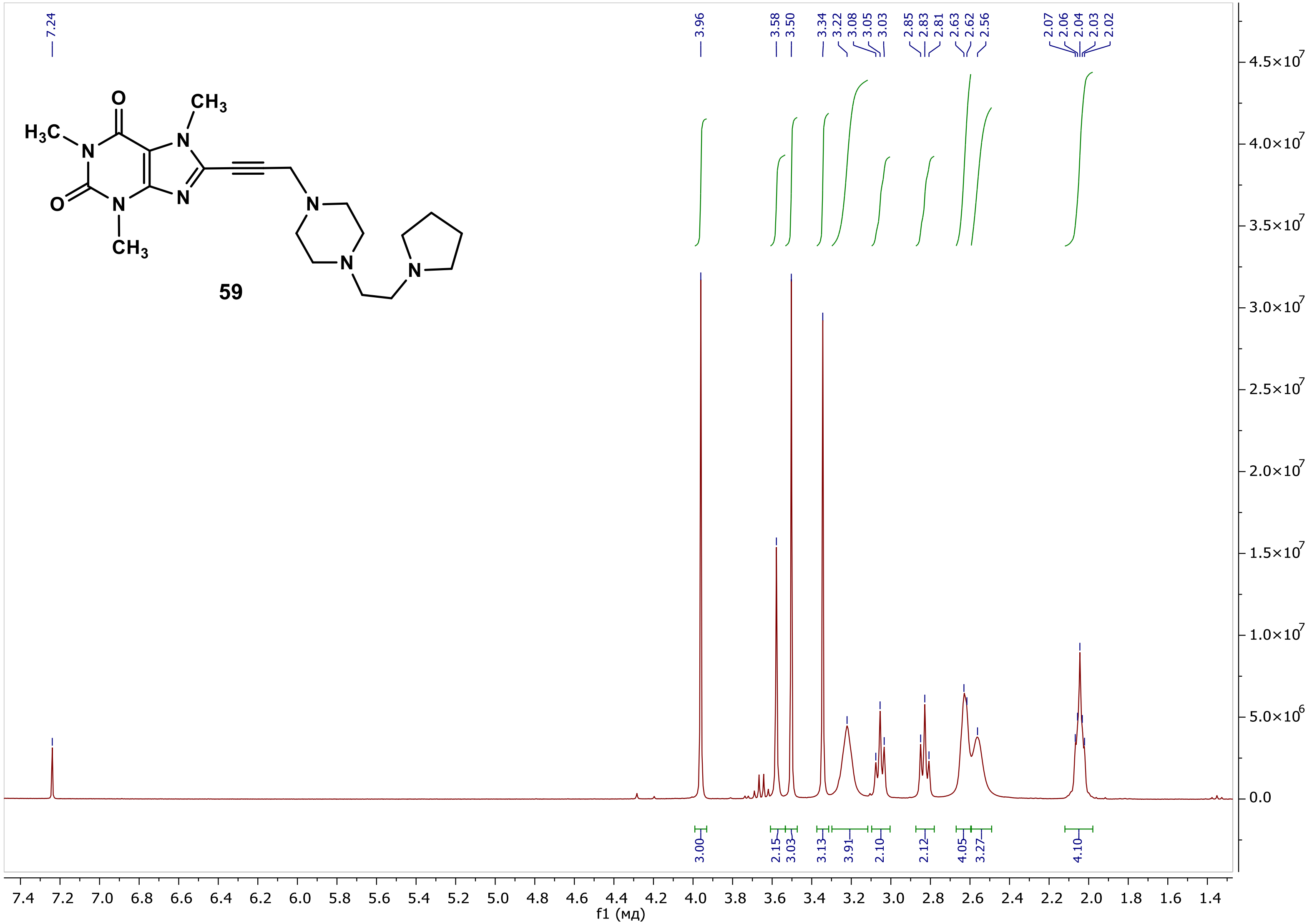

1,3,7-Trimethyl-8-(3-(4-(2-(pyrrolidin-1-yl)ethyl)piperazin-1-yl)prop-1-yn-1-yl)-3,7-dihydro-1*H*-purine-2,6-dione (^{13}C NMR, 101 MHz, $CDCl_3$)

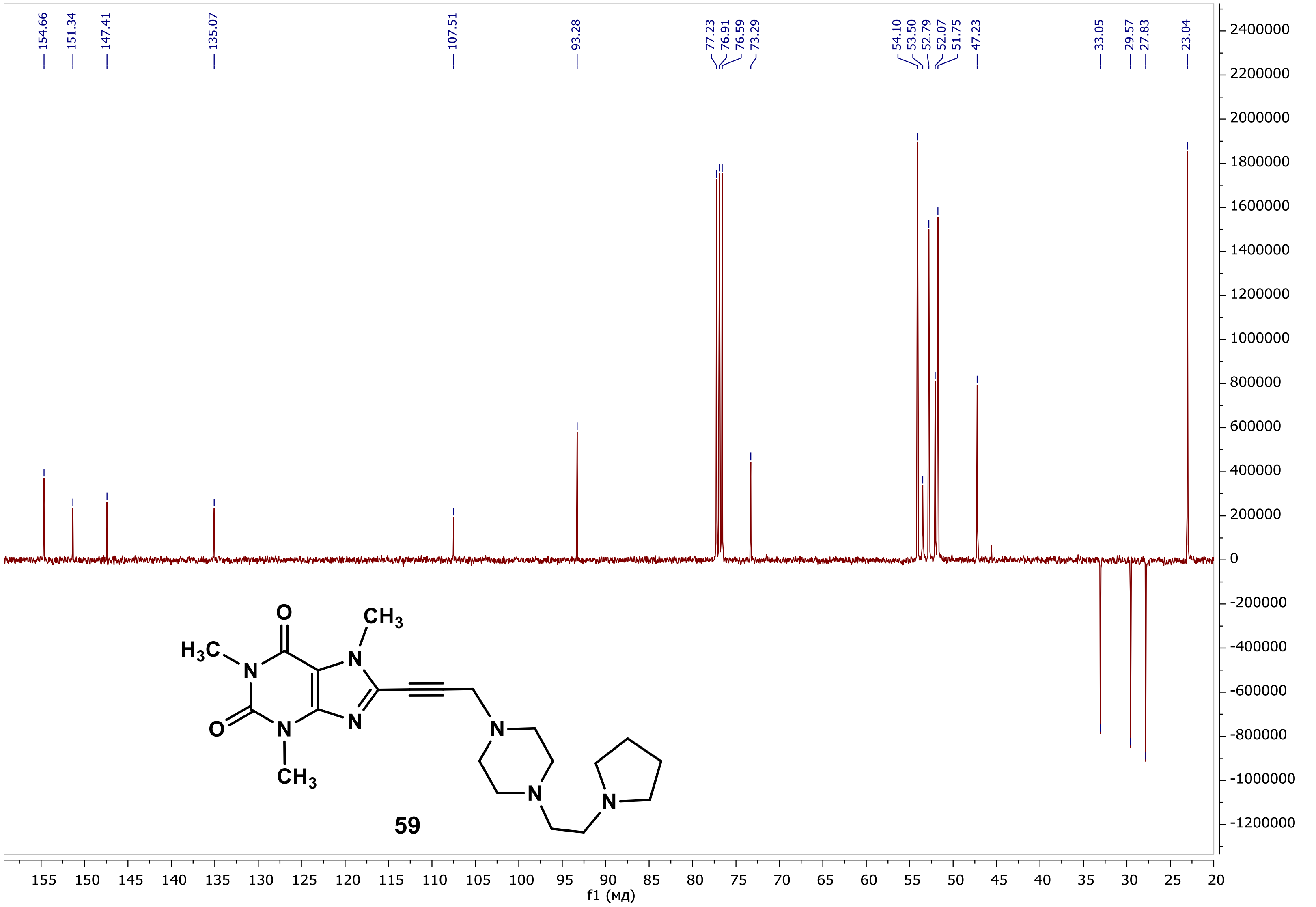

(1,3,7-Trimethyl-8-(4-methylpiperidin-1-yl)-3,7-dihydro-1*H*-purine-2,6-dione (**60**) (^{1}H NMR, 500 MHz, $CDCl_3$)

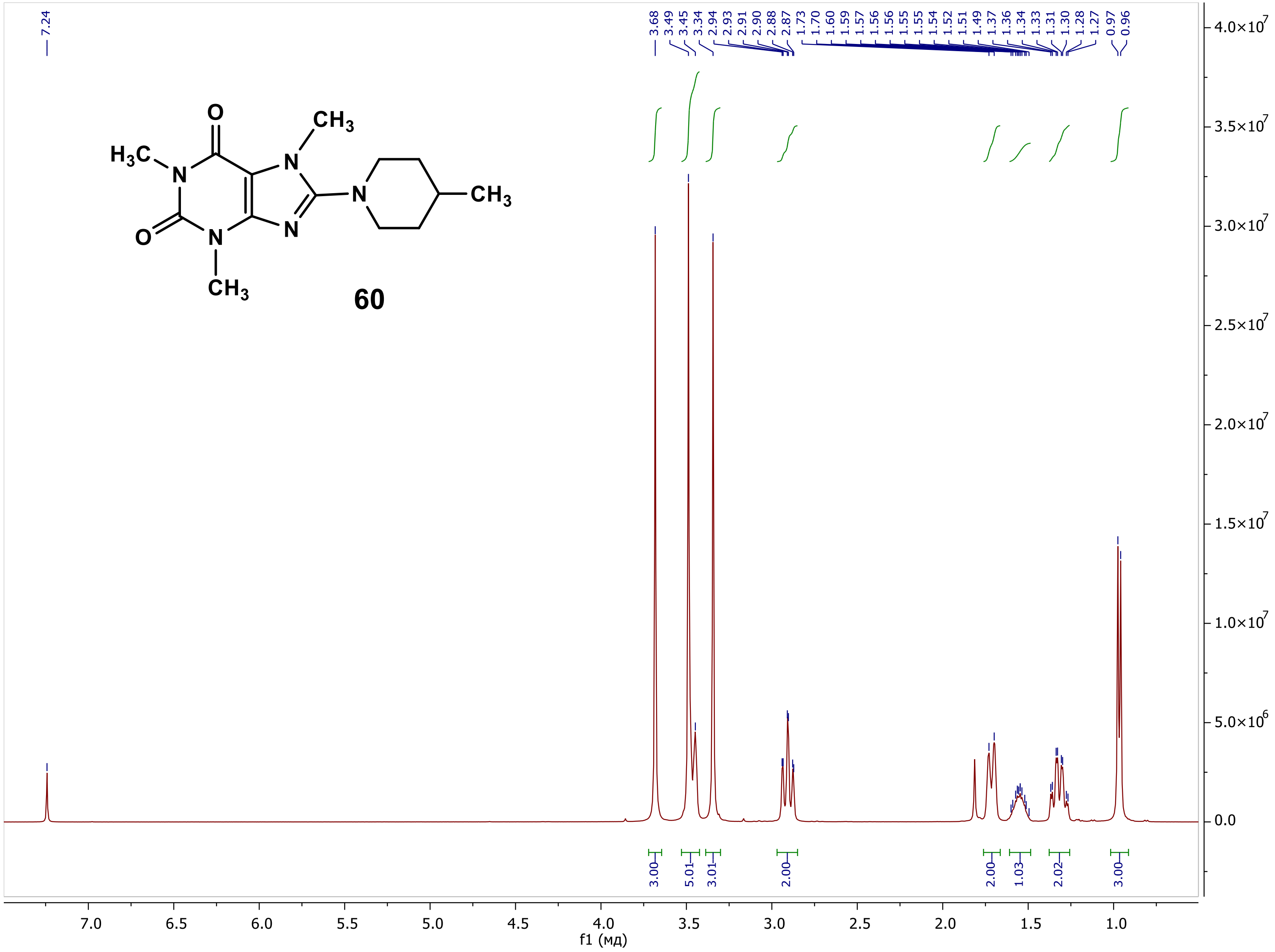

1,3,7-Trimethyl-8-(4-methylpiperidin-1-yl)-3,7-dihydro-1*H*-purine-2,6-dione (**60**) (^{13}C NMR, 126 MHz, $CDCl_3$)

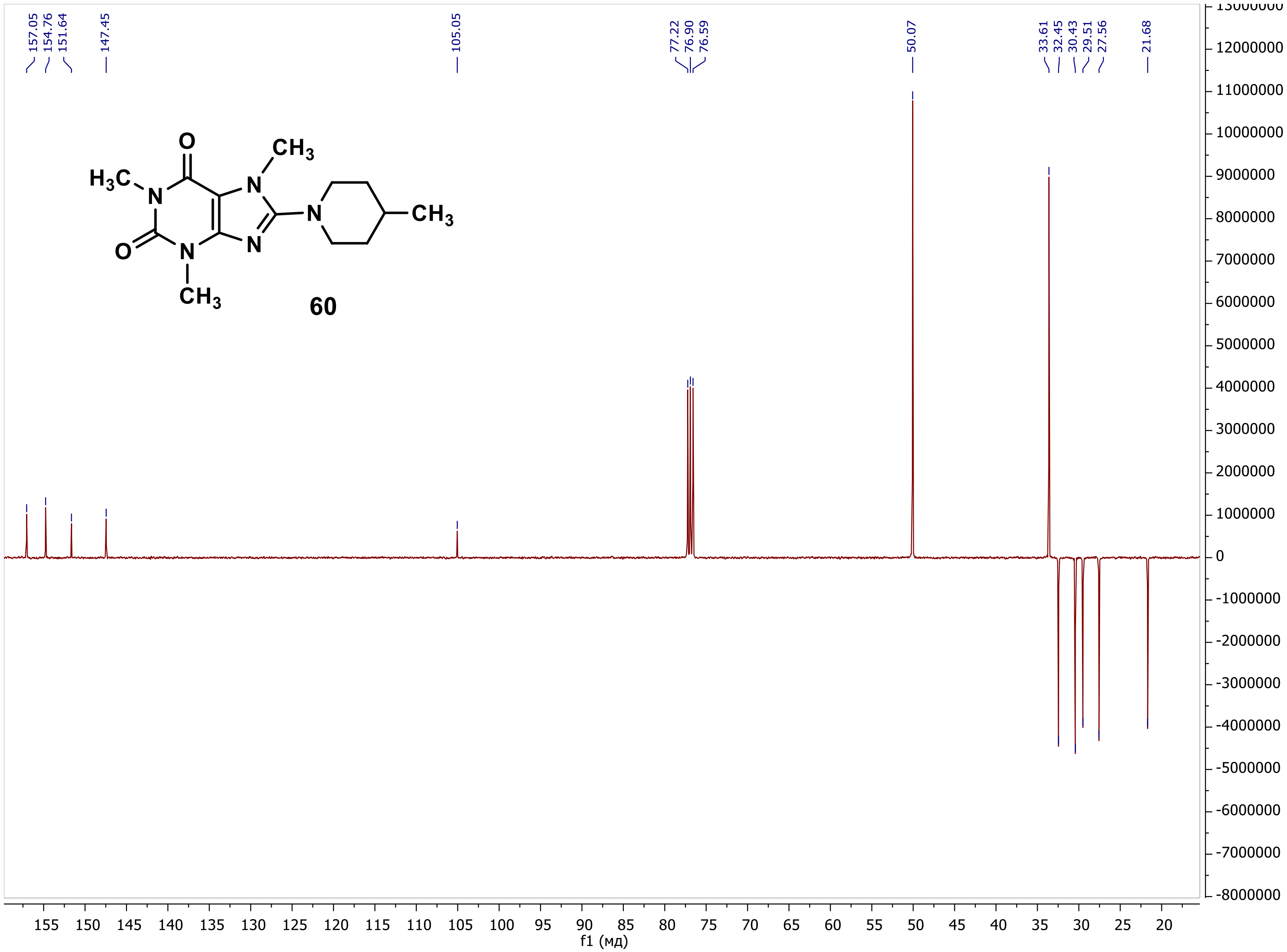

tert-Butyl 4-(1,3,7-trimethyl-2,6-dioxo-3,7-dihydro-1*H*-purin-8-yl)piperazine-1-carboxylate (**61**) (^{1}H NMR, 500 MHz, $CDCl_3$)

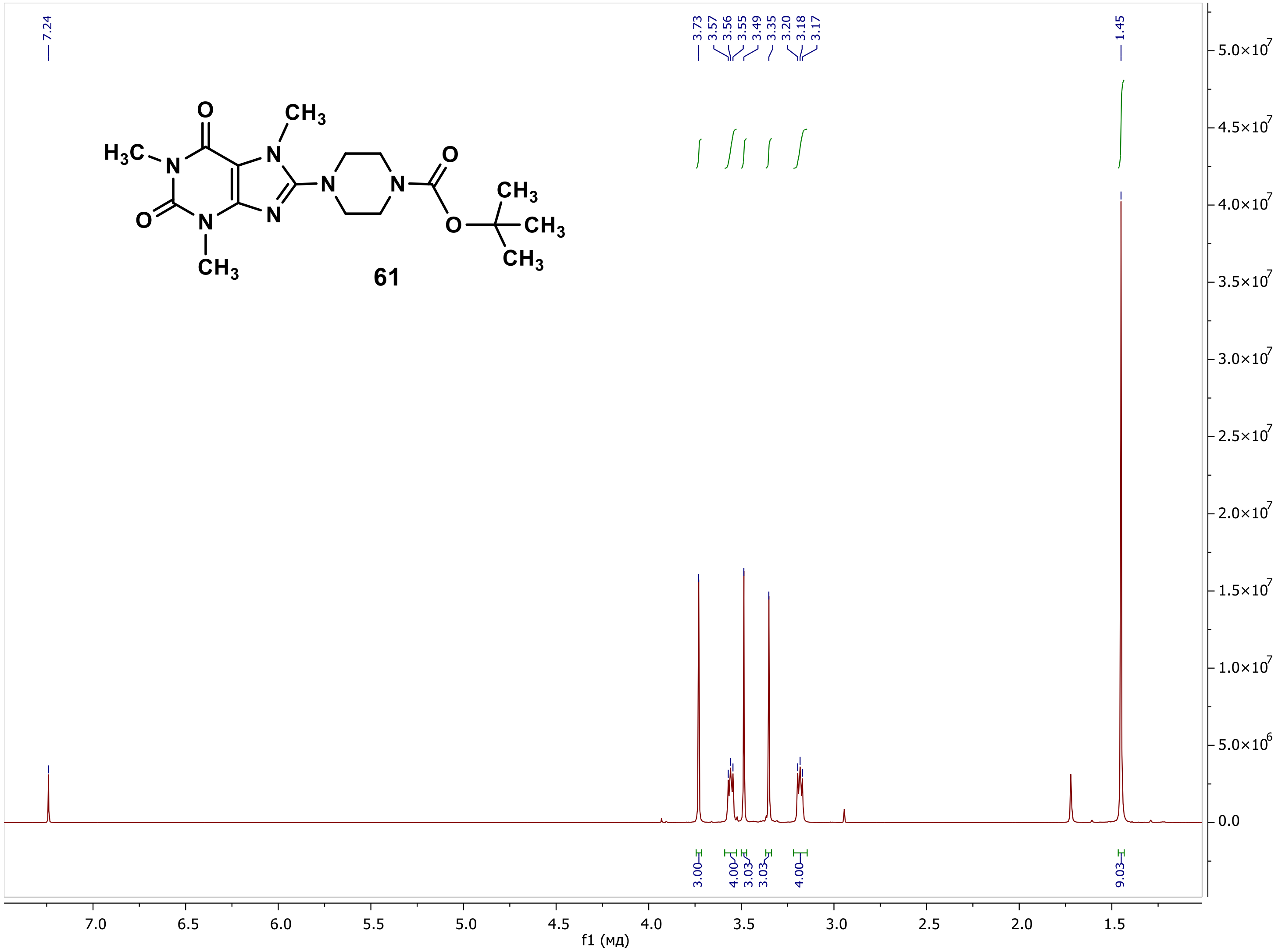

tert-Butyl 4-(1,3,7-trimethyl-2,6-dioxo-3,7-dihydro-1*H*-purin-8-yl)piperazine-1-carboxylate (**61**) (^{13}C NMR, 126 MHz, $CDCl_3$)

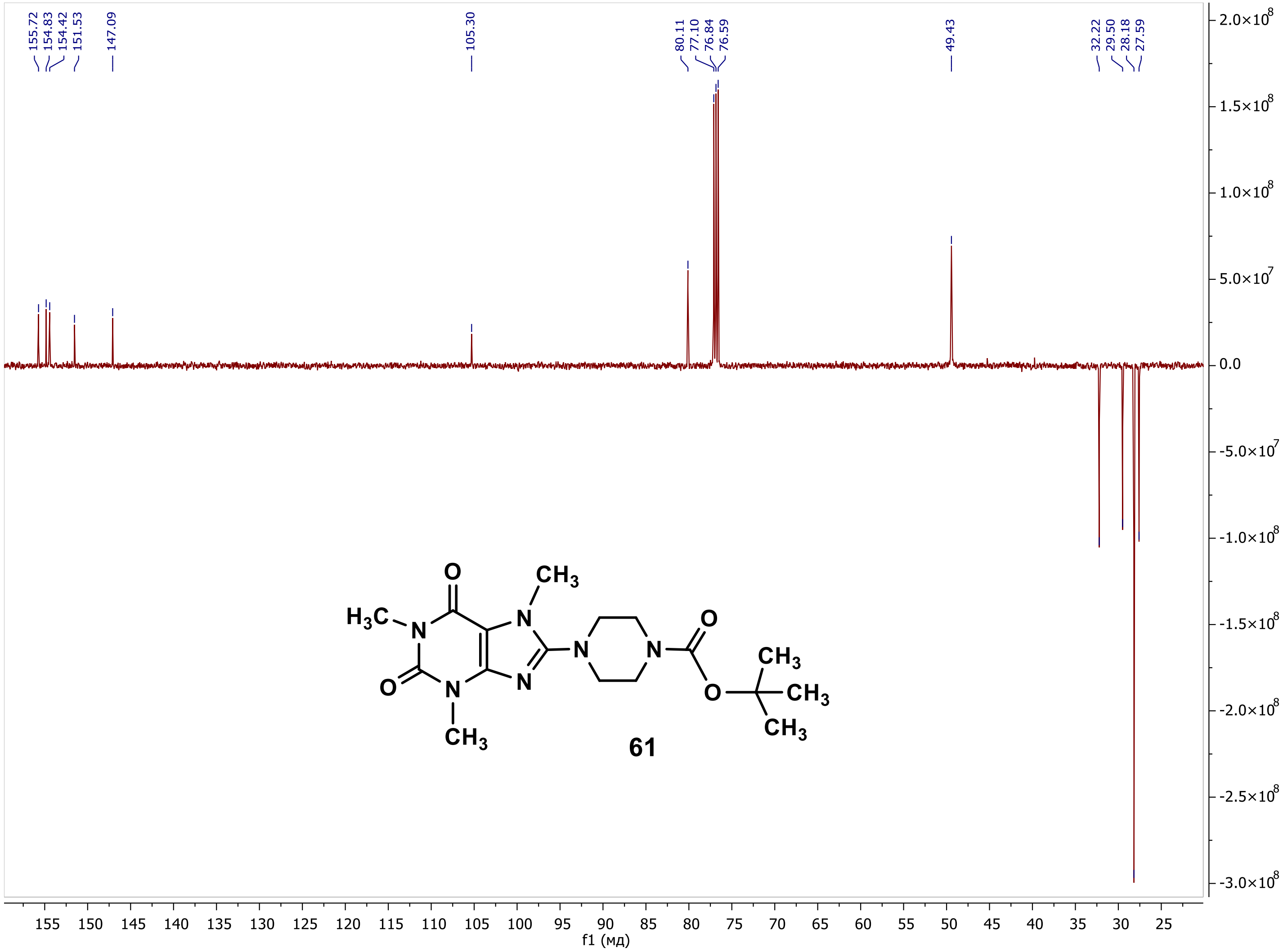

1-(4-(Diisopropylamino)but-2-yn-1-yl)-3,7-dimethyl-3,7-dihydro-1*H*-purine-2,6-dione (**64**) (^{1}H NMR, 400 MHz, $CDCl_3$)

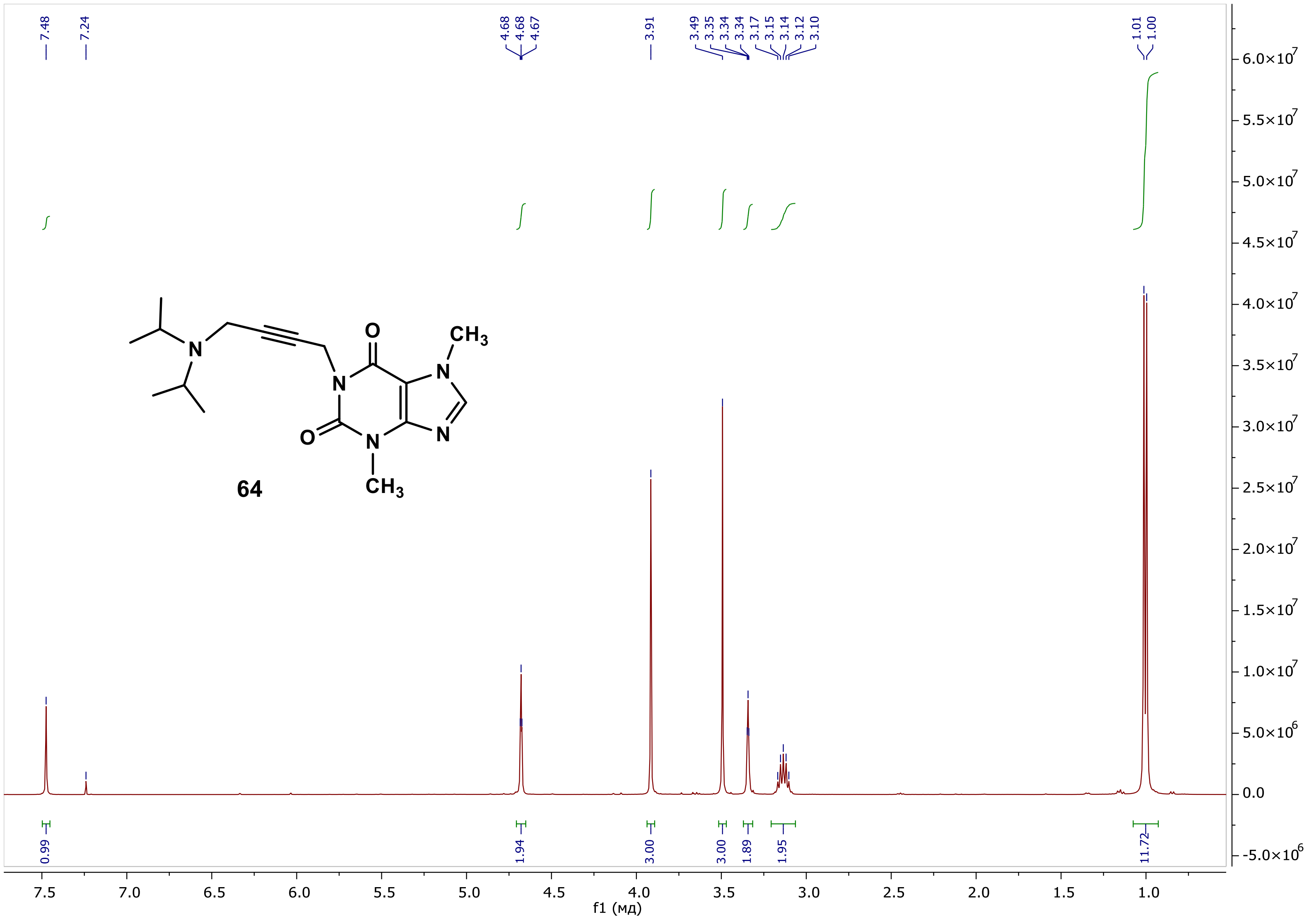

1-(4-(Diisopropylamino)but-2-yn-1-yl)-3,7-dimethyl-3,7-dihydro-1*H*-purine-2,6-dione(**64**) (^{13}C NMR, 101 MHz, $CDCl_3$)

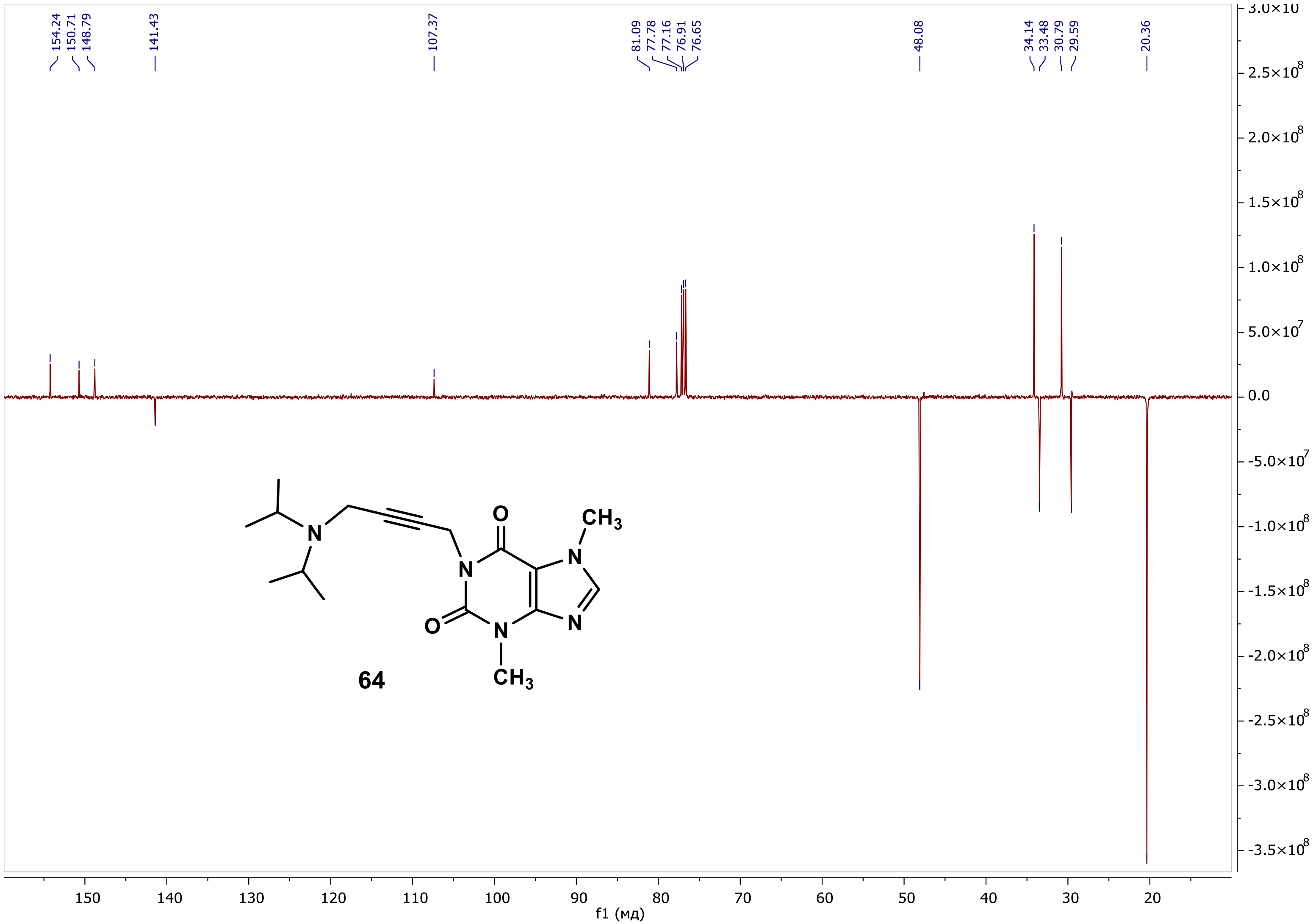

64

1-(4-(Azocan-1-yl)but-2-yn-1-yl)-3,7-dimethyl-3,7-dihydro-1*H*-purine-2,6-dione (**65**) (^{1}H NMR, 400 MHz, $CDCl_3$)

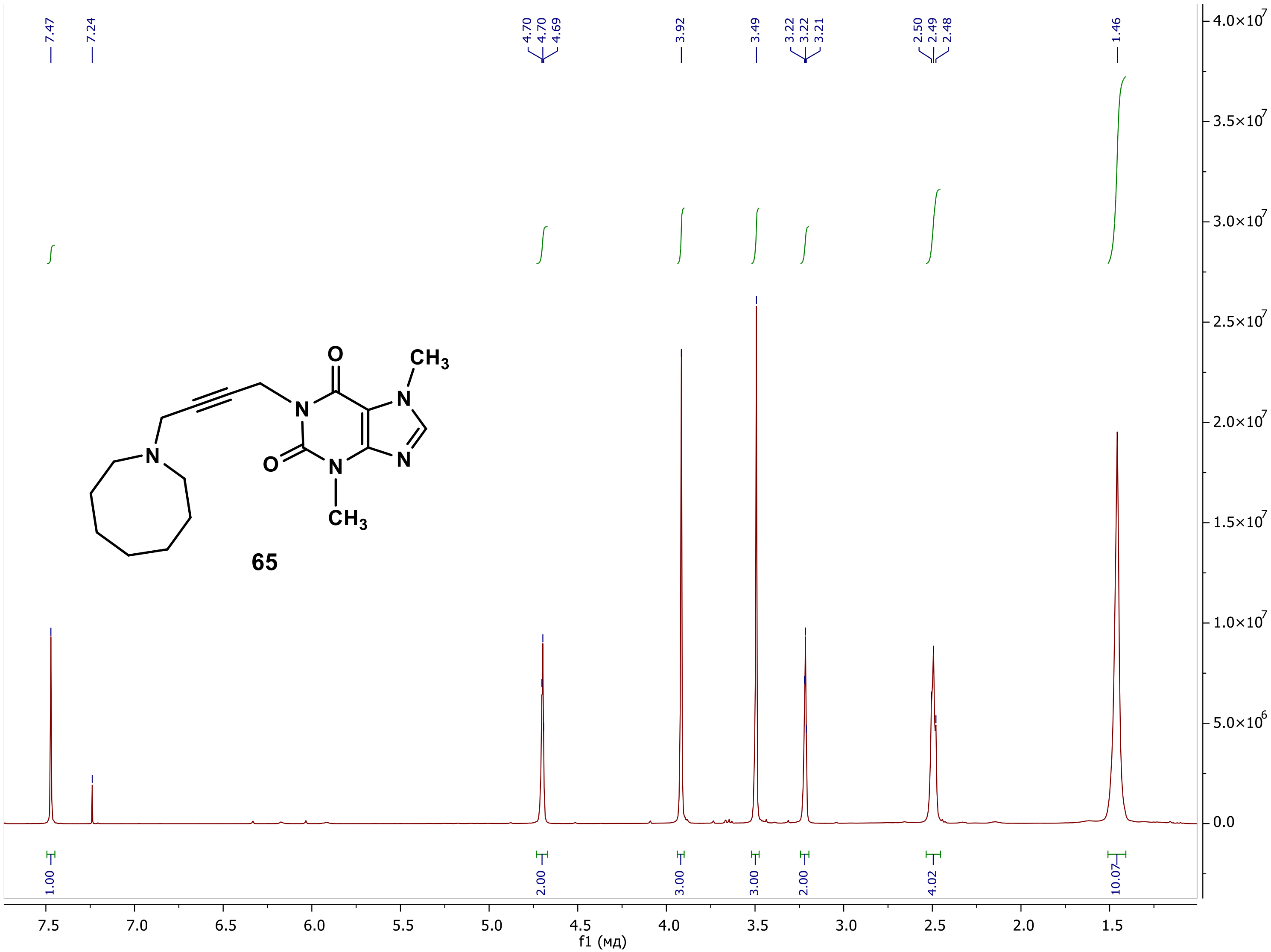

1-(4-(Azocan-1-yl)but-2-yn-1-yl)-3,7-dimethyl-3,7-dihydro-1*H*-purine-2,6-dione (**65**) (^{13}C NMR, 101 MHz, $CDCl_3$)

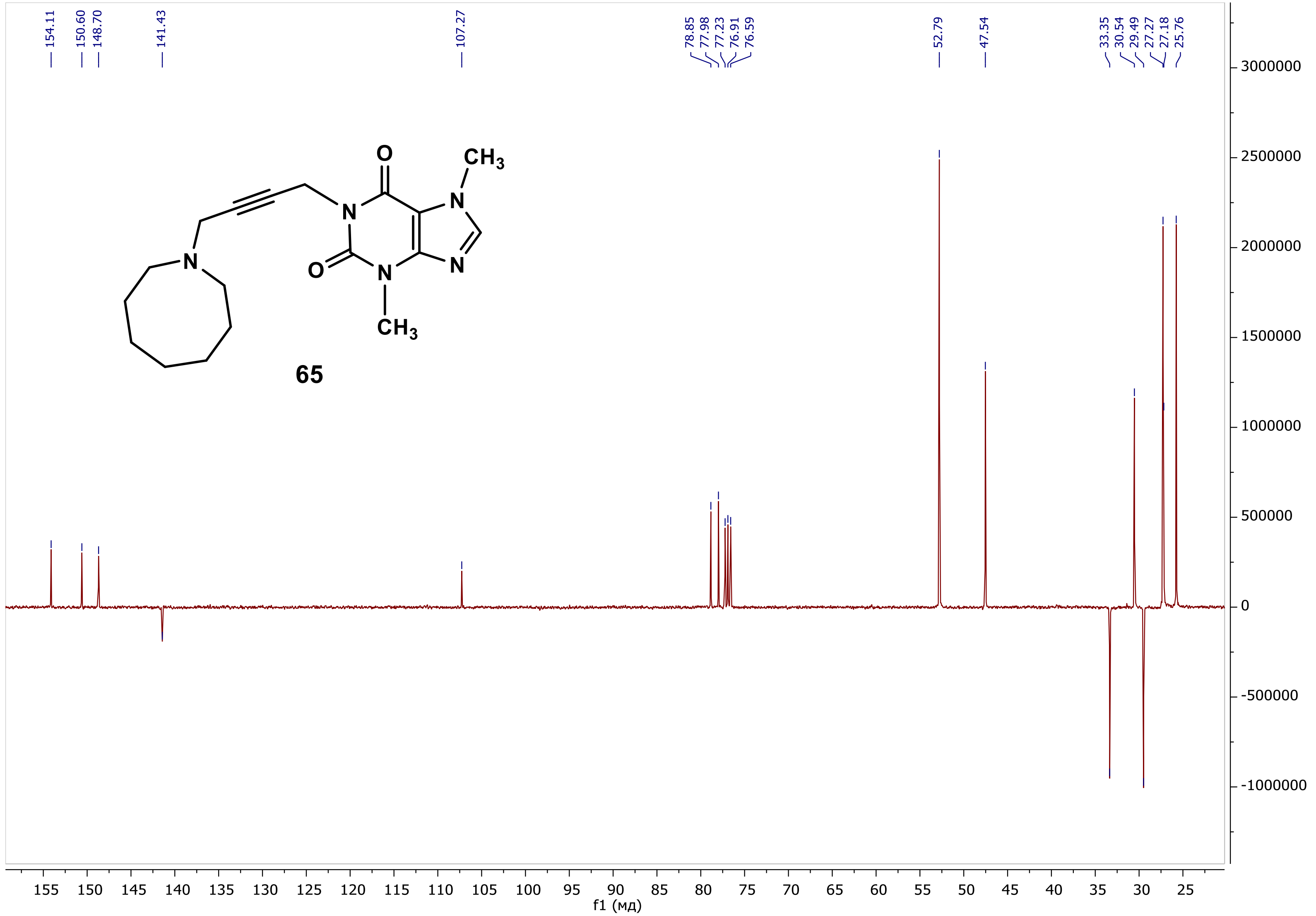

3,7-Dimethyl-1-(4-(4-(2-(pyrrolidin-1-yl)ethyl)piperazin-1-yl)but-2-yn-1-yl)-3,7-dihydro-1*H*-purine-2,6-dione hydrate (**66**) (^{1}H NMR, 400 MHz, $CDCl_3$)

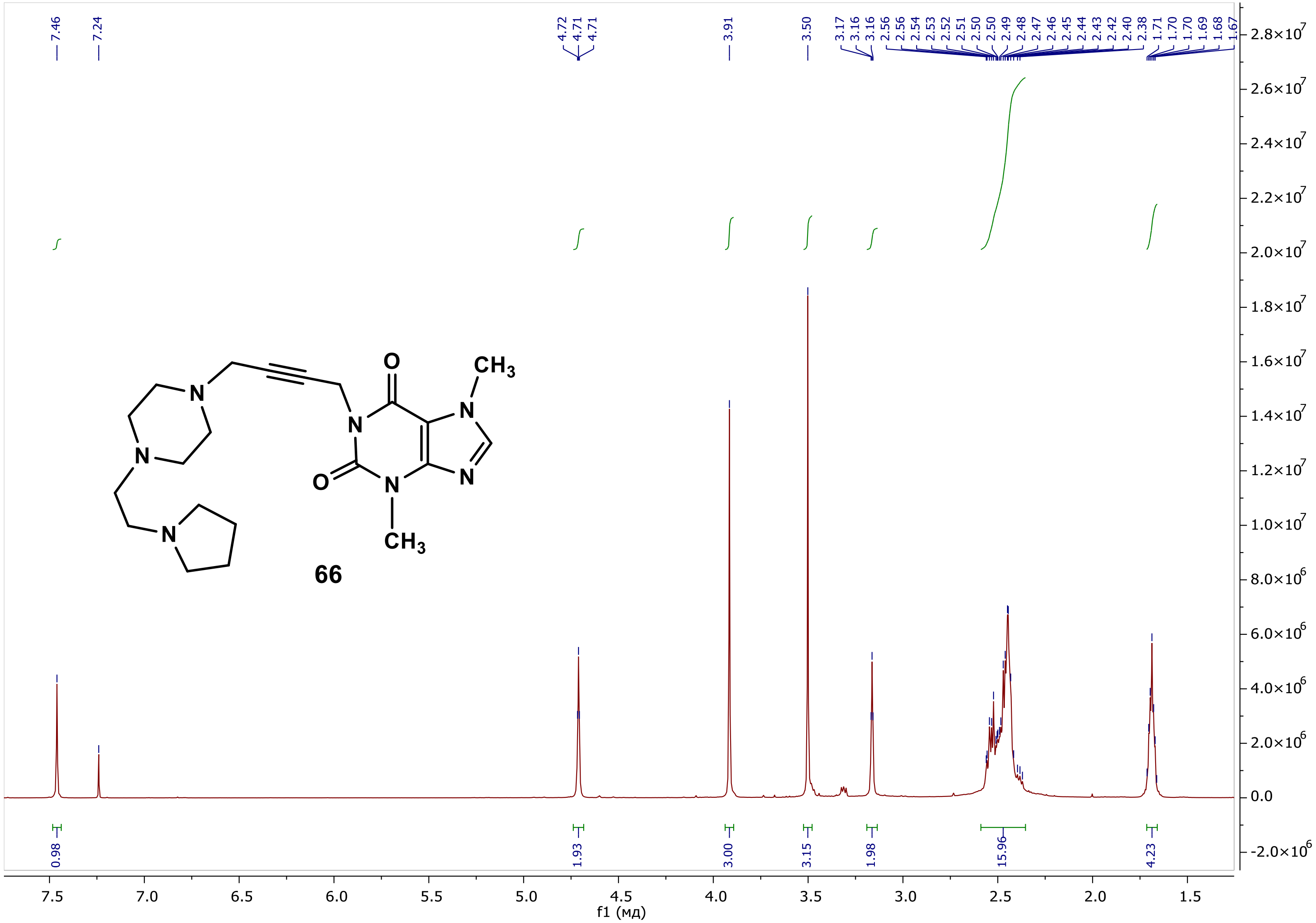

3,7-Dimethyl-1-(4-(4-(2-(pyrrolidin-1-yl)ethyl)piperazin-1-yl)but-2-yn-1-yl)-3,7-dihydro-1*H*-purine-2,6-dione (**66**) (^{13}C NMR, 101 Hz, $CDCl_3$)

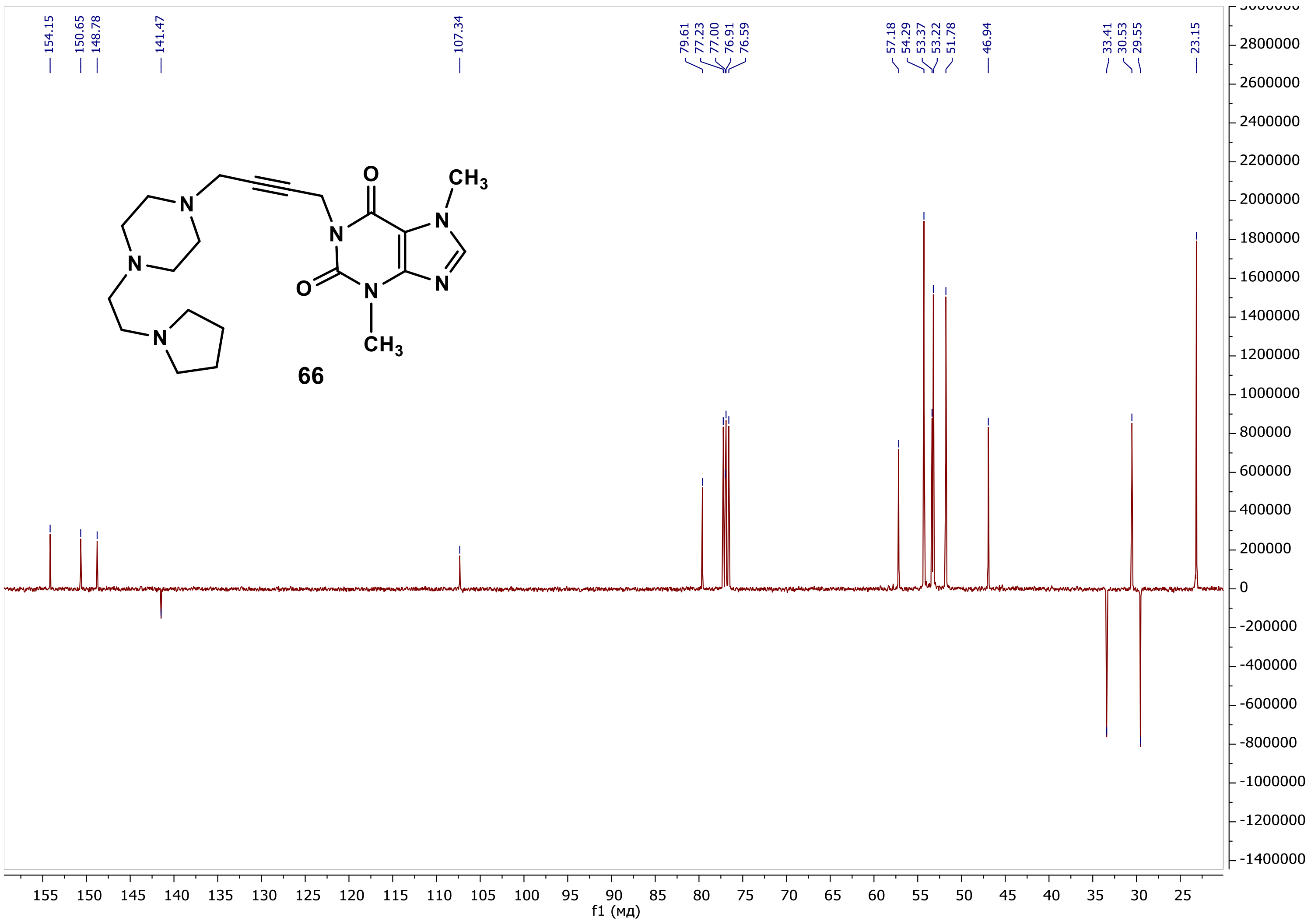

7-(4-(Diisopropylamino)but-2-yn-1-yl)-1,3-dimethyl-3,7-dihydro-1*H*-purine-2,6-dione (**68**) (^{1}H NMR, 400 MHz, CDCl$_3$)

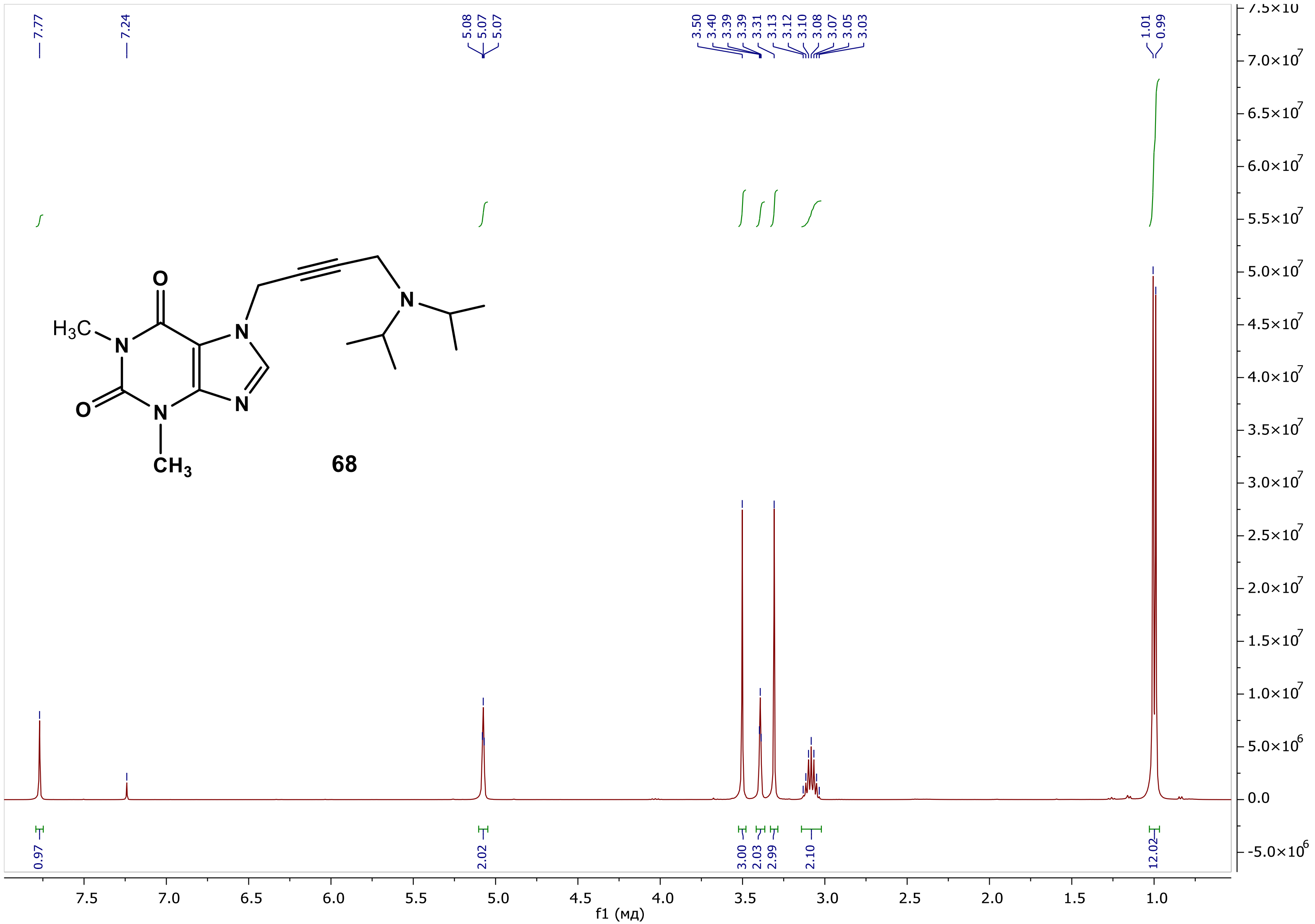

7-(4-(Diisopropylamino)but-2-yn-1-yl)-1,3-dimethyl-3,7-dihydro-1*H*-purine-2,6-dione (**68**) (^{13}C NMR, 126 MHz, $CDCl_3$)

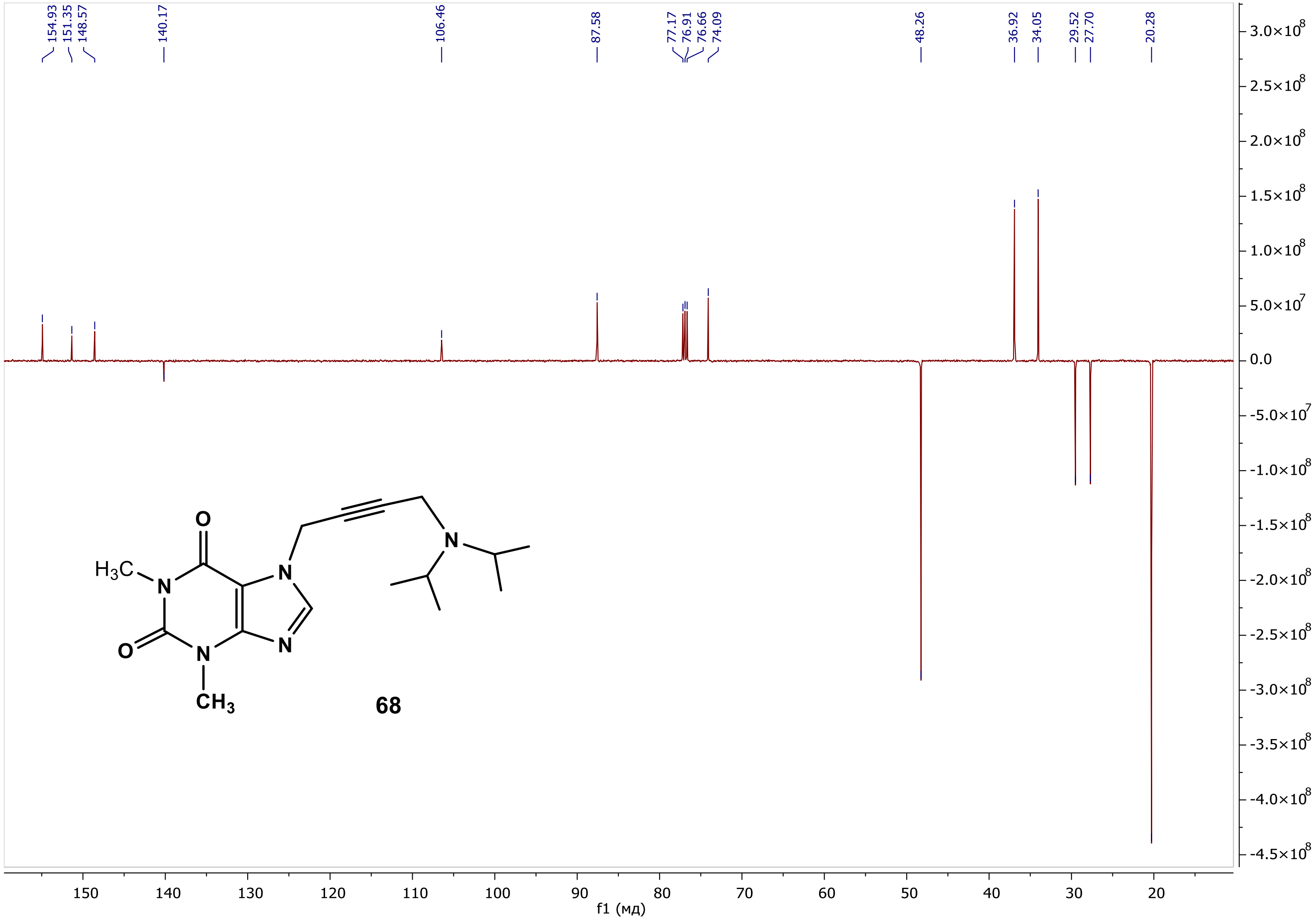

7-(4-(Azocan-1-yl)but-2-yn-1-yl)-1,3-dimethyl-3,7-dihydro-1*H*-purine-2,6-dione (**69**) (^{1}H NMR, 400 MHz, $CDCl_3$)

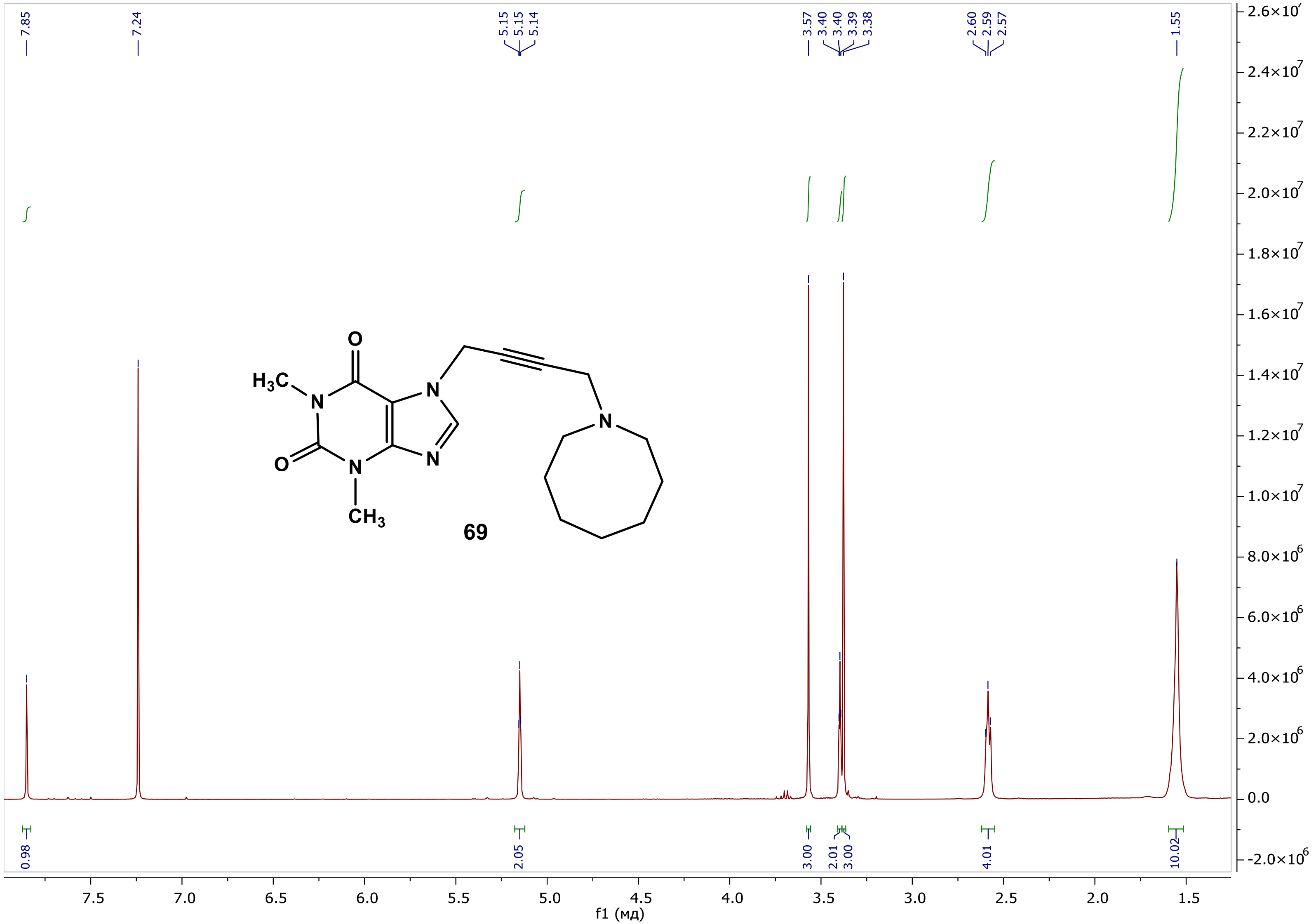

7-(4-(Azocan-1-yl)but-2-yn-1-yl)-1,3-dimethyl-3,7-dihydro-1*H*-purine-2,6-dione (**69**) (^{13}C NMR, 126 MHz, CDCl$_3$)

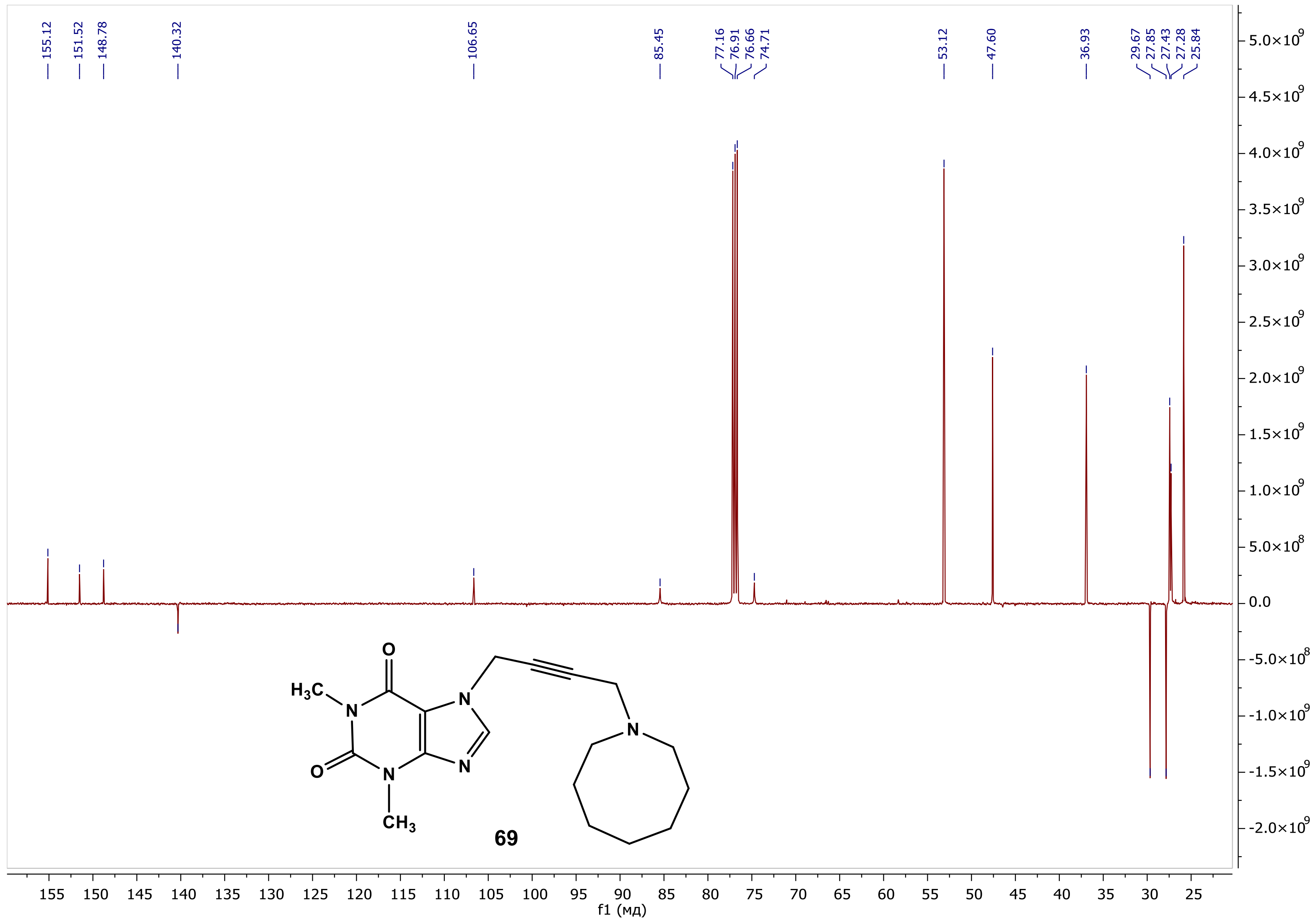

1,3-Dimethyl-7-(4-(4-(2-(pyrrolidin-1-yl)ethyl)piperazin-1-yl)but-2-yn-1-yl)-3,7-dihydro-1*H*-purine-2,6-dione (**70**) (^{1}H NMR, 300 MHz, $CDCl_3$)

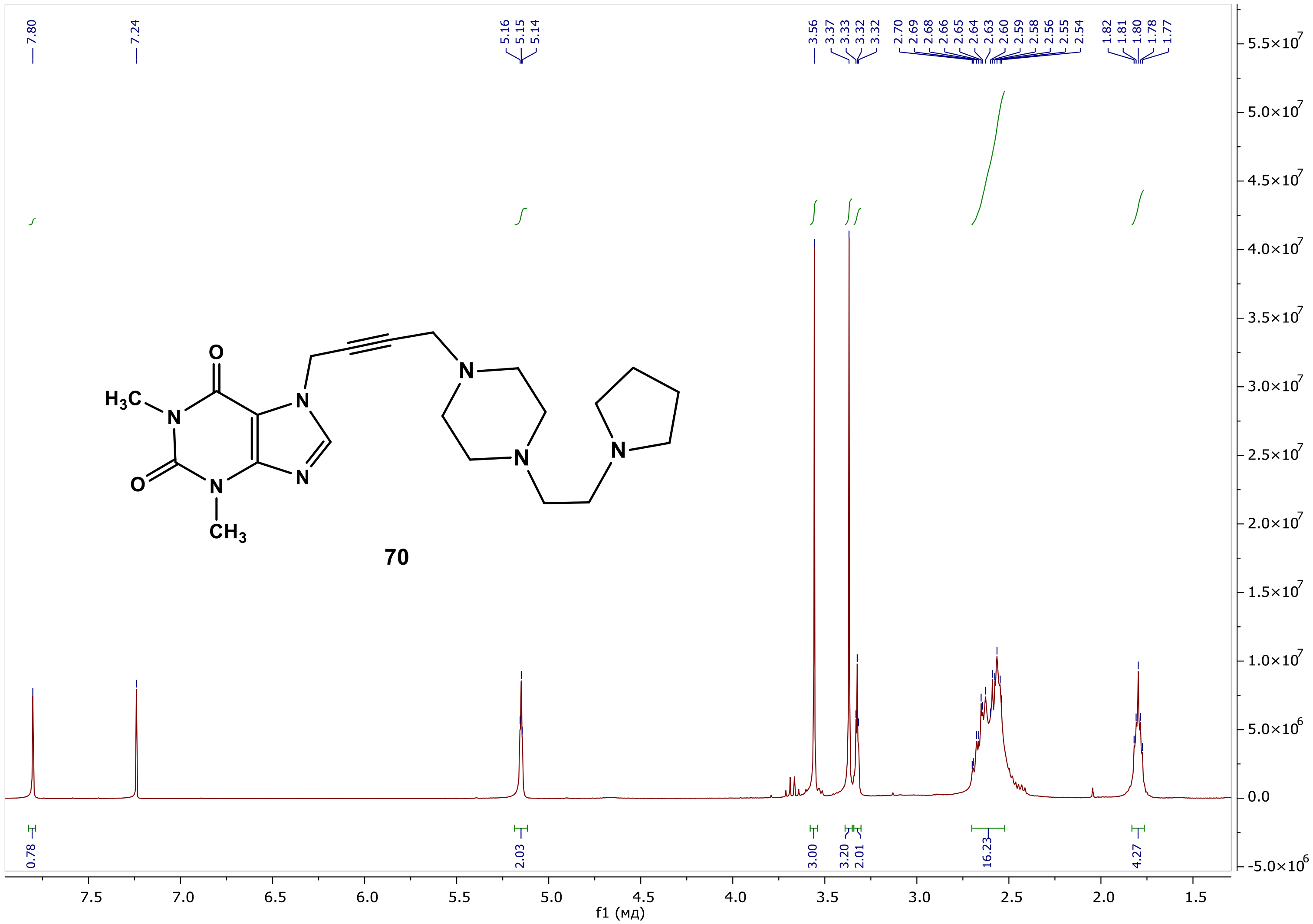

1,3-Dimethyl-7-(4-(4-(2-(pyrrolidin-1-yl)ethyl)piperazin-1-yl)but-2-yn-1-yl)-3,7-dihydro-1*H*-purine-2,6-dione (**70**) (^{13}C NMR, 75 MHz, $CDCl_3$)

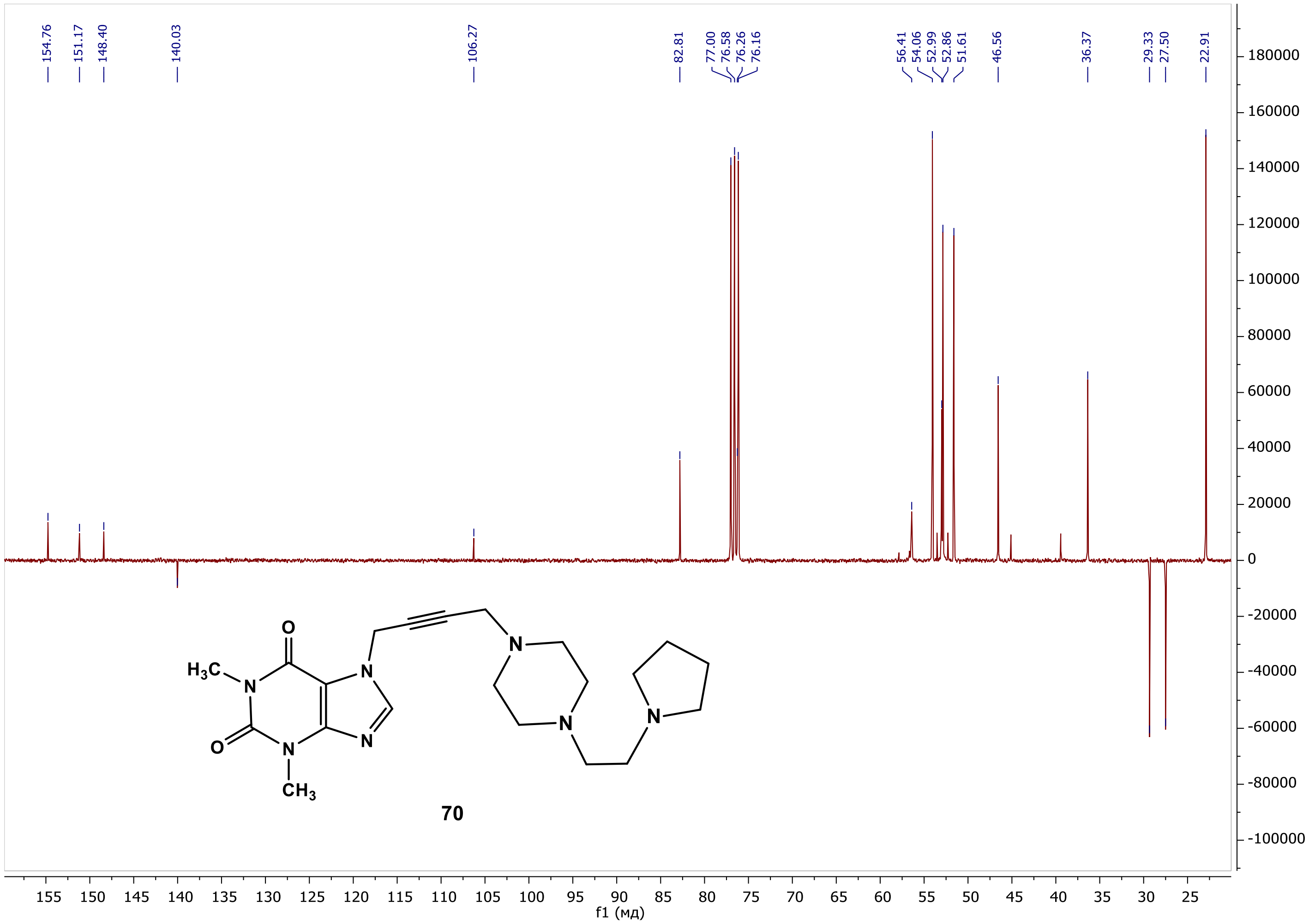

70

S5A) AChE inhibition by compound **28**

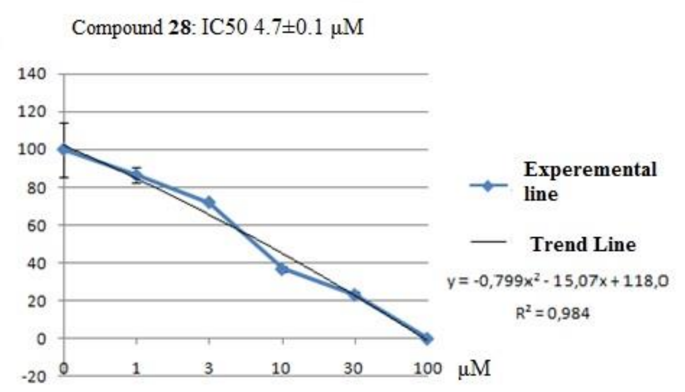

S5B) AChE inhibition by compound **64**

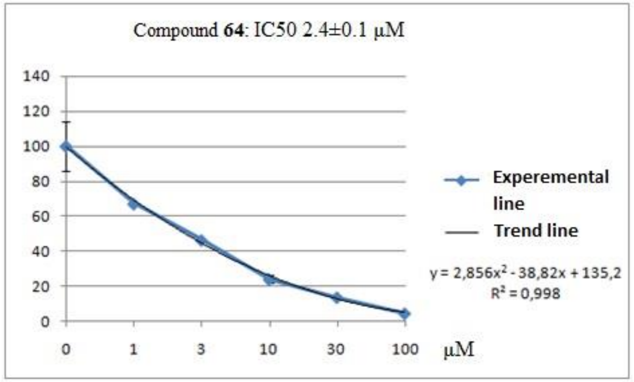

S5C) AChE inhibition by compound **70**

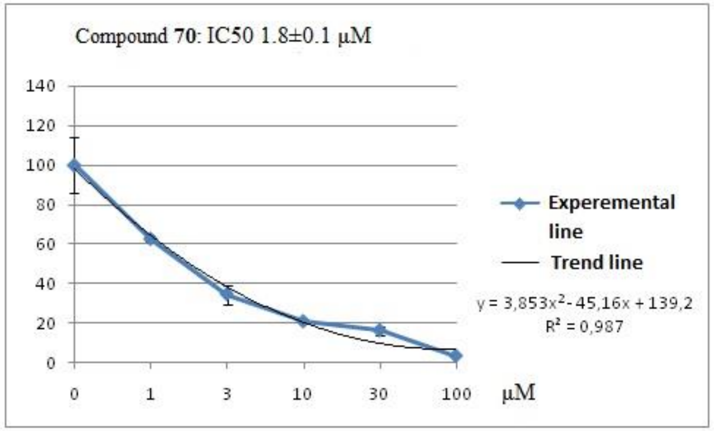

5D) AChE inhibition by compound **65**

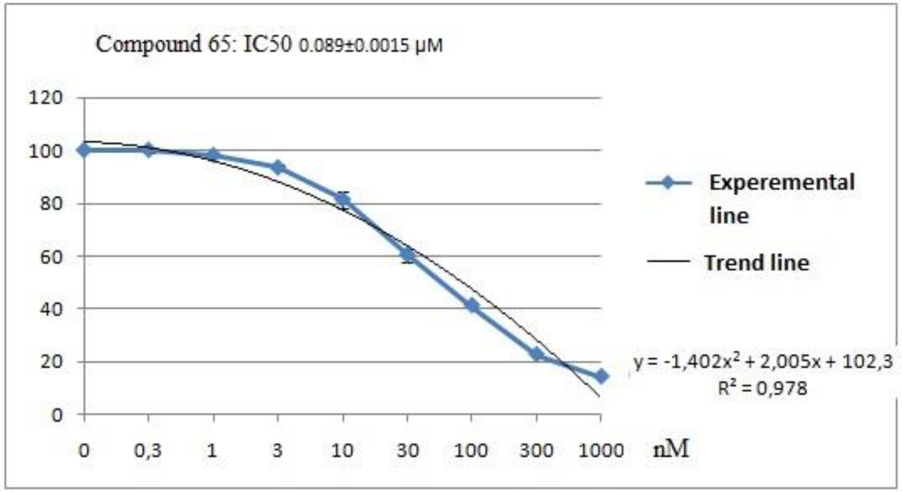

5E) AChE inhibition by compound **66**

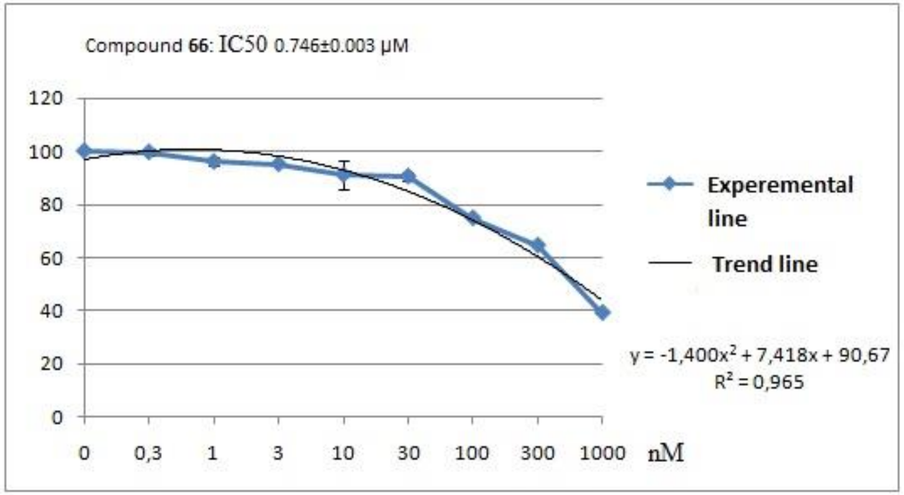

Figure S5A-E. Plots dependency of percent of inhibition – concentration for AChE inhibition by compounds **28**, **64**, **65**, **66**, **70.**

S6A) RMSD for Donepezil in complex with AChE

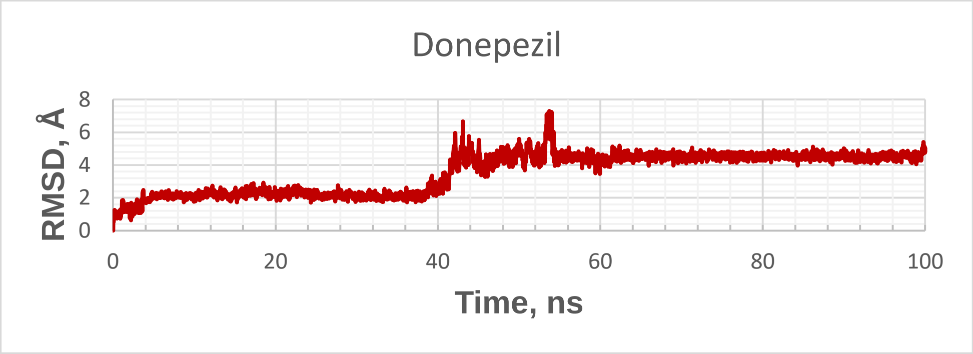

S6B) RMSD for compound **59** in complex with AChE

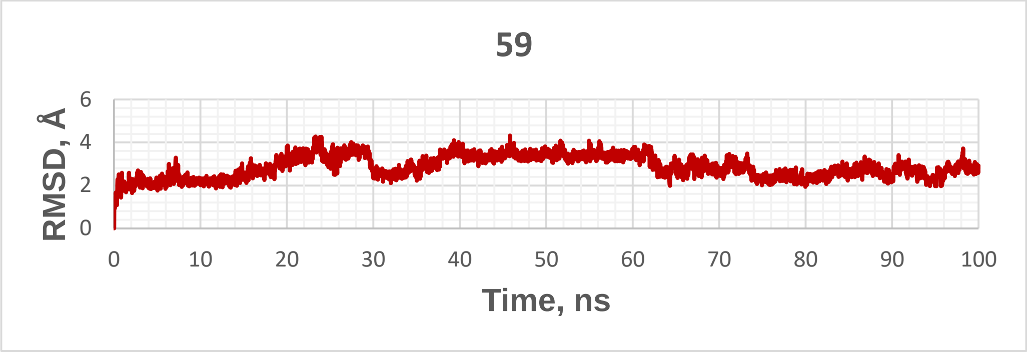

S6C) RMSD for compound **64** in complex with AChE

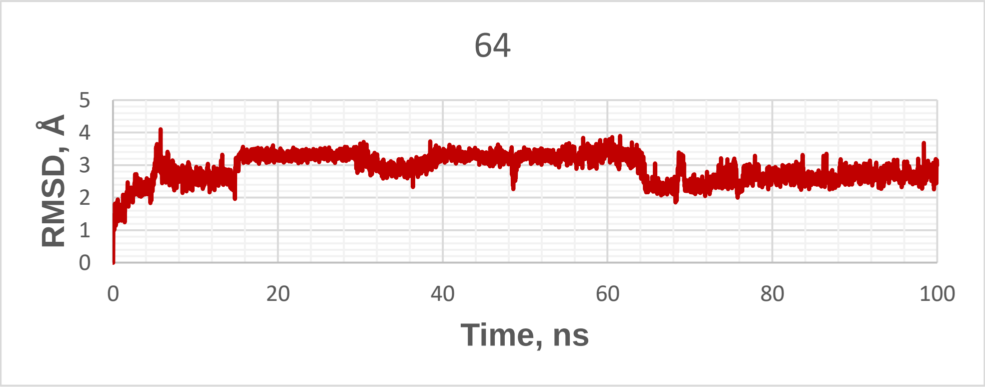

S6D) RMSD for compound **69** in complex with AChE

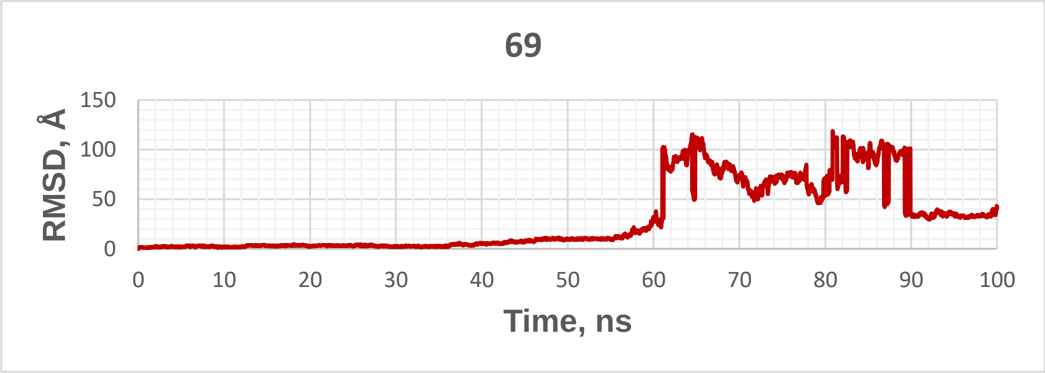

Fig. S6A-D. Calculated RMSD of ligand atomic coordinates over simulation time, obtained as a result of molecular dynamics simulation for protein-ligand complexes.
